# Machine Learning- and AI-Driven QSAR Models for the Discovery of Novel Potential Fungicides: FAPI (Fungicide Targeting Acid Phosphatase Inhibition)

**DOI:** 10.1021/acs.jafc.5c07939

**Published:** 2025-12-24

**Authors:** María Gálvez-Llompart, Riccardo Zanni, Yandira Morales, Álvaro Polonio, Dolores Fernández-Ortuño, Alejandro Pérez-García, Facundo Pérez-Giménez

**Affiliations:** † Department of Preventive Medicine and Public Health, Food Science, Toxicology and Forensic Medicine, Faculty of Pharmacy and Food Science, University of Valencia, Burjassot 46100, Valencia, Spain; ‡ Department of Physical Chemistry, University of Valencia, 46100 Burjassot, Valencia, Spain; § Department of Microbiology, Faculty of Science, Instituto de Hortofruticultura Subtropical y Mediterránea La Mayora, IHSM-UMA-CSIC, 16752 University of Malaga, Málaga 29071, Spain

**Keywords:** QSAR, AI, molecular docking, fungicide, acid phosphatase, *Podosphaera xanthii*, *Botrytis cinerea*

## Abstract

Fungal pathogens like *Podosphaera xanthii* (powdery mildew) and *Botrytis cinerea* (gray mold) cause significant agricultural losses, with fungicide resistance escalating due to the overreliance on conventional treatments. Consequently, the development of sustainable alternatives with novel modes of action is imperative for future crop protection. Acid phosphatases (APs), which play a key role in fungal phosphate metabolism and virulence, have emerged as promising molecular targets. To accelerate the identification of fungicides targeting acid phosphatase inhibition (FAPI), machine learning (ML), and artificial intelligence (AI)-driven quantitative structure–activity relationship (QSAR) models incorporating topological molecular descriptors have been employed to predict fungicidal activity. The experimental validation of the predicted candidates highlights the promising potential of these novel fungicides and underscores the value of ML- and AI-based QSAR methodologies in the development of next-generation, resistance-breaking fungicidal agents.

## Graphical abstract

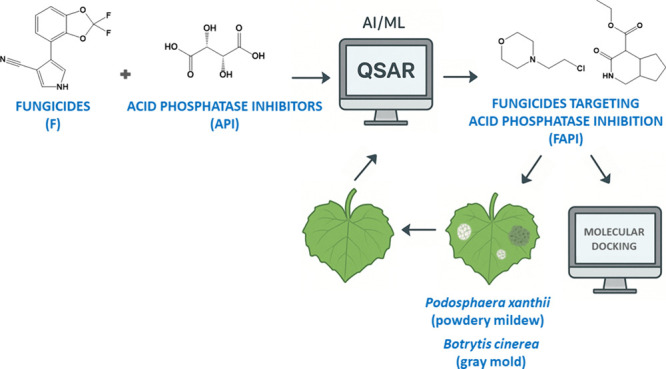

## 1 Introduction

Fungal diseases continue to pose a major threat to global agriculture, causing billions of dollars in crop losses annually. Among the most damaging pathogens highlight *Podosphaera xanthii* and *Botrytis cinerea*, which causes the cucurbit powdery mildew and gray mold diseases, respectively.^1,2^ Traditional fungicides, while initially effective, are increasingly compromised due to the emergence of resistant strains, often driven by the overuse or repeated application of compounds with the same mode of action.^3,4^ As a result, the Fungicide Resistance Action Committee (FRAC) has classified both as a high-risk pathogen for fungicide resistance development (FRAC, 2019).^5^ In addition, according to the “Farm-to-Fork” strategy of the recent European Green Deal, the number of available fungicides will be reduced in the near future (European Commission, 2022).^6^ To ensure long-term crop protection and reduce the environmental burden of overusing chemical treatments, it is critical to develop fungicides with novel mechanisms of action that can break resistance cycles. A fungicide with a new mode of action is less likely to encounter pre-existing resistance and can be integrated into rotation or mixture strategies to delay further resistance evolution. Furthermore, new targets are essential for managing pathogens that have already developed broad-spectrum resistance to existing fungicides.

Acid phosphatases (APs) are enzymes involved in key metabolic functions such as phosphate mobilization, intracellular signaling, and cell wall remodeling. These enzymes are critical for fungal growth and adaptation, especially under nutrient-limited conditions. Inhibiting APs could effectively impair the development, pathogenicity, and survival of fungal pathogens. Recent research has pointed to APs as potential antifungal targets due to their central role in fungal metabolism and their structural differences from human or plant counterparts.^7^ Supporting this hypothesis, recent work has identified two haustorium-specific secreted acid phosphatases, PxSHAP1 and PxSHAP2, in *P. xanthii* that are essential for its development. These enzymes were validated functionally through yeast complementation assays and gene silencing (ATM-HIGS, *Agrobacterium tumefaciens*-mediated host-induced gene silencing), which resulted in up to 80% reduction in fungal growth, demonstrating their pivotal role in phosphorus acquisition during biotrophy.^8^

Furthermore, inhibition assays using known acid phosphatase inhibitors, such as sodium orthovanadate and ammonium molybdate, significantly restricted fungal growth, reinforcing the idea that targeting APs represents a promising route for the development of new fungicides with a novel mode of action.^9,10^ In this study, the inhibition of fungal acid phosphatases was explored as an innovative mode of action for the development of potential fungicidal agents with the aim of addressing the increasing challenge of fungicide resistance.

To achieve this objective, a computational approach integrating machine learning (ML) and artificial intelligence (AI)-driven quantitative structure–activity relationship (QSAR) models was developed. QSAR models are based on a quantitative relation settled between the molecular structure of different chemicals and their biological activity by utilizing various algorithms,^11^ including those based on ML and AI. Chemical structures of compounds can be mathematically defined using various molecular descriptors when developing QSAR models. These descriptors include constitutional, geometrical, thermodynamic, electronic, and topological descriptors. Among these, topological descriptors hold particular importance, as they emphasize the role of molecular topology in defining the relationship between a compound’s chemical structure and its biological activity.

These descriptors represent molecular structures by treating atoms as nodes and chemical bonds as edges, focusing on connectivity and interatomic relationships within a graph, a concept derived from graph theory applied to chemistry. Together with topo-chemical descriptors, they have been extensively applied to the discovery of novel fungicides.^12–15^

The integration of ML and AI into QSAR modeling has revolutionized its application by significantly enhancing its predictive capabilities.^16^ These algorithms can effectively capture both linear and nonlinear relationships between molecular descriptors and biological activity, thereby improving the accuracy and reliability of QSAR models.^17,18^ Once the chemo-mathematical pattern associated with a specific biological activity is identified, QSAR models can predict the activity of novel, untested compounds or facilitate the screening of potential candidates.^19,20^

In a recent investigation^8,21^ acid phosphatase inhibition (API) has been proposed as a promising target for exerting fungicidal activity, representing a novel mode of action. To explore this potential, a computational approach leveraging advanced techniques, including ML and AI-driven QSAR models, has been employed to discover new FAPI (**F**ungicides targeting **A**cid **P**hosphatase **I**nhibition) compounds.

## 2 Materials and Methods

### 2.1 Computational Strategy for the Discovery of Novel Potential Fungicides

Figure 1 presents a schematic overview of the key steps in our computational strategy for the discovery of novel potential fungicides, specifically, FAPI compounds. This strategy integrates data-driven approaches, molecular modeling, and experimental validation to identify and characterize potential fungicidal candidates.

**Figure 1:**
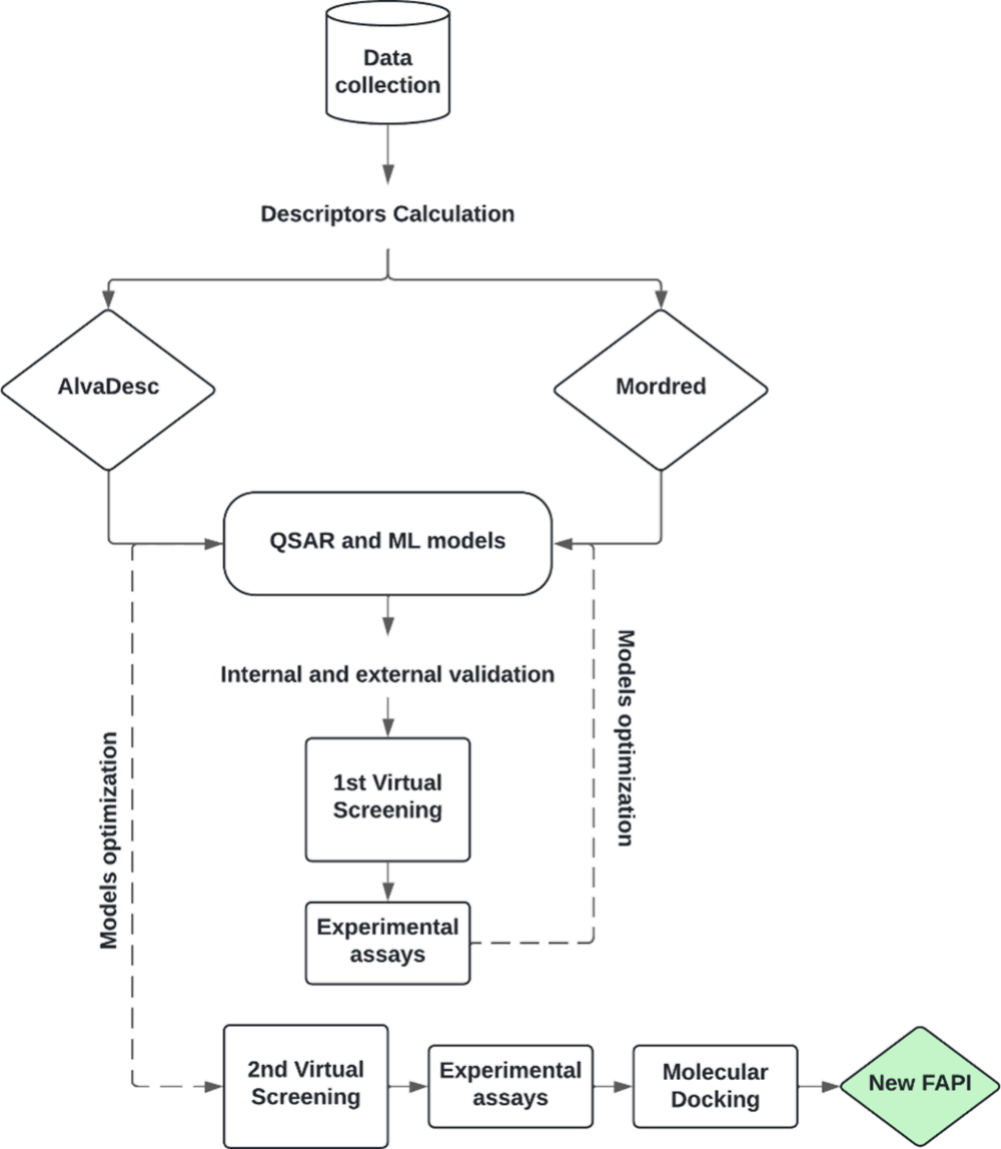
Design of new FAPI using QSAR and ML approaches.

Details of each step in this strategy are elaborated in the following subsections and in the Supporting Information.

#### 2.1.1 Data Collection

A comprehensive database of chemicals with known fungicidal activity was compiled from multiple sources, including the World Intellectual Property Organization (WIPO) database,^22^ the Fungicide Resistance Action Committee (FRAC) database,^23^ and the PubChem database.^24^ For WIPO and PubChem, the Browse PubChem Compound TOC feature was utilized, focusing on the WIPO PATENTSCOPE patent library and the agrochemical library, respectively, to extract relevant information on fungicidal compounds, whereas data from FRAC was consulted directly.

Additionally, a smaller data set of compounds with reported acid phosphatase inhibitory activity was curated from literature sources.^25–35^ Inactive compounds were selected from the literature and from the WIPO database, prioritizing those with chemical similarity to the active compounds within the training set.

#### 2.1.2 Molecular Descriptor Calculation

Two-dimensional (2D) molecular descriptors were calculated for the entire data set using two software tools, AlvaDesc and Mordred, to ensure a comprehensive characterization of the compounds’ physicochemical and topological properties. Mordred, an open-source software, computes over 1800 molecular descriptors derived from 2D and 3D structures.^36^ AlvaDesc, a commercial software, calculates more than 5300 molecular descriptors (2D and 3D).^37^ Together, these tools provide extensive coverage of molecular properties, including constitutional, topological, geometrical, electronic, and pharmacophore-based features, making them invaluable for molecular characterization and the development of robust QSAR models.

#### 2.1.3 ML- and AI-Driven QSAR Modeling

A computational strategy combining ML and AI was employed to identify novel fungicides through QSAR modeling. Two complementary approaches were used: linear discriminant analysis (LDA) for linear classification and artificial neural networks (ANN) for nonlinear pattern recognition classify compounds as active (fungicidal or acid phosphatase inhibitors) or inactive.

Molecular descriptors were selected via a forward stepwise method based on statistical significance (*p*-value), ensuring the inclusion of only the most relevant variables. Model robustness was evaluated through Wilks’ lambda, *F*-value, *p*-value, and classification accuracy derived from confusion matrices.

When data size allowed, external validation (20% test set) was applied to fungicidal activity models, while internal validation was used for API models due to the limited data set.

Eighteen QSAR models were developed using descriptors from AlvaDesc and Mordred, encompassing: 12 models for fungicidal activity (Figure S1) and 6 models for API (Figure S2). The training data sets were compiled from WIPO, FRAC, and PubChem databases, as well as literature sources, integrating active fungicidal compounds and acid phosphatase inhibitors with inactive compounds obtained from WIPO and the literature to ensure chemical diversity. Each data set generated paired LDA and ANN models.

All models were trained and validated using STATISTICA (v12, StatSoft),^38^ ensuring predictive reliability for identifying novel FAPI.

#### 2.1.4 Virtual Screening of Chemical Database

Two virtual screening (VS) campaigns were conducted using commercial databases to identify potential FAPI, fungicides with a novel mechanism of action. The eMolecules database,^39^ containing a comprehensive collection of 8 million commercially available chemical compounds for drug discovery and development, was utilized. Additionally, the Specs database,^40^ known for its extensive range of synthetic and natural compounds, and the Sigma-Aldrich catalog,^41^ a well-established resource offering a wide variety of biochemical and organic molecules, were also screened. These databases provided broad chemical diversity, enhancing the likelihood of identifying promising novel fungicides.

### 2.2 Experimental and Biological Assays for Fungicidal Activity

The fungicidal activity of the compounds identified through virtual screenings using ML- and AI-driven QSAR models was evaluated using four different assays: leaf disc or spore germination assays and plant or fruit in two different phytopathogenic fungus, *P. xanthii* and *B. cinerea*, respectively.

#### 2.2.1 Leaf Disc Assay

For *P. xanthii*, zucchini cotyledon discs and the 2086 isolate were used. The assay was conducted as previously described by Fernández-Ortuño et al.^42^ Briefly, zucchini cotyledon discs (8 mm diameter) from 8-day-old plants were immersed during 1 h in 3 mL of aqueous solution and 0.02% of the surfactant Tween20 with different concentrations (10, 100 μM, and 1 mM) of the acid phosphatase inhibitors. Discs treated with sterile distilled water and the fungicide fluopyram at the same concentrations were used as negative and positive controls, respectively. Then, discs were dried, deposited on plates with Bertrand medium (saccharose 40 g L^–1^, benzimidazole 30 mg L^–1^, agar 10 g L^–1^, pH 7.0) and inoculated in the center with 30 μL of a spore suspension of *P. xanthii* (1 × 10^5^ spores/ml) being, finally, incubated under a 16 h light/8 h dark cycle at 25 °C for 8 days. After incubation, fungal development on the leaf surface was evaluated by an image analysis. For this analysis, pictures were captured with a digital camera at a fixed distance of 20 cm from the discs. The pictures were analyzed using Fiji image-processing software ImageJ to calculate the surface covered by powdery mildew symptoms in each disc. The assay was repeated 3 times.

#### 2.2.2 Spore Germination Assay

For *B. cinerea*, isolate 91 was used. The assay was performed in 24-well plates which contained CZAPEK DOX medium (sodium nitrate 2 g L^–1^, potassium chloride 0.5 g L^–1^, magnesium glycerophosphate 0.5 g L^–1^, ferrous sulfate 0.01 g L^–1^, potassium sulfate 0.35 g L^–1^, sucrose 30 g L^–1^, agar 12 g L^–1^, pH 6.8) and different concentrations (10, 100 μM, and 1 mM) of the acid phosphatase inhibitors. Wells treated with sterile distilled water and the fungicide fludioxonil at the same concentrations were used as negative and positive controls, respectively. The wells were then inoculated with 30 μL drop of a spore suspension (1.5 × 10^5^ spores/ml) of the *B. cinerea* isolate being incubated at 23 °C for 16 h. After that, the germ tubes were measured using the FIJI image program and the corresponding graphs were made based on percentage of inhibition. The assay was repeated three times.

#### 2.2.3 Plant Assays

For *P. xanthii* plant assays, 3-week-old melon plants and the protocols described by Romero et al.,^43^ were used. The plants were inoculated by spreading with the selected acid phosphatase inhibitors at 5 mM and sterile distilled water or the fungicide fluopyram at the recommended field dose were used as negative and positive controls, respectively. 24 h after inoculation, leaves were sprayed with a suspension of *P. xanthii* conidia (1 × 10^4^ conidia/mL) until the point of runoff. Plants were then maintained in a greenhouse under a 16 h light/8 h dark cycle at 25 °C for 12 days. After this incubation, disease symptoms were evaluated by image analysis, as indicated above. Five plants were used per treatment. The assay was repeated three times.

#### 2.2.4 Fruit Assays

For *B. cinerea* fruit assays, commercial apple fruits were used and inoculated with a 30 μL drop of a spore suspension (1 × 10^3^ conidia/ml) of the 91 isolate of *B. cinerea*, following the protocol described by Leisen et al.^44^ The selected compounds were then applied by spreading with the different acid phosphatase inhibitors at the concentrations of 5 mM. The fruits were placed in closed boxes containing moistened filter paper and incubated under a 16 h light/8 h dark cycle at 25 °C for 6 days until the development of symptoms. Disease symptoms (fruit surface covered by fungi) were evaluated by digital analysis, as indicated above. Water and the commercial fungicide fludioxonil at the recommended field dose were used as negative and positive controls, respectively. The assay was repeated three times.

### 2.3 Optimization of the In Silico Strategy with Experimental Assay Results

Using the experimental data on fungicidal activity provided by the UMA laboratory, four new QSAR models were developed following the same methodology described previously. Their goal was to identify the chemometric patterns associated with compounds showing fungicide experimental activity.

These optimized models, together with the previously developed models, were then employed in a second virtual screening campaign to identify potential FAPI.

As shown in Figure S3, the workflow integrates experimental validation into the ML- and AI-driven QSAR modeling strategy. Each computational set included both the LDA and ANN models based on AlvaDesc and Mordred descriptors.

### 2.4 Molecular Docking

To confirm the proposed mechanism of action for the new fungicidal compounds and explore their binding to acid phosphatase (AP), molecular docking simulations were performed using Maestro (Schrödinger Suite, v2022-4).^45^

Two AP models were used: the *Aspergillus niger* enzyme (PDB ID: 1QFX) and a homology model of *P. xanthii* generated with I-TASSER (model ID: 15569).^46^ For 1QFX, the active site was defined by residues Arg62, His63, Arg66, Asp75, Arg156, Glu272, His318, and Asp319.^47^ In the *P. xanthii* model, potential binding sites were identified using SiteMap, selecting the top-ranked pocket for docking simulations.

Protein and ligand structures were prepared with the Protein Preparation Wizard and LigPrep, respectively. Docking grids were generated by using the Glide module, and simulations were conducted under standard precision (SP) conditions with default parameters.

## 3 Results and Discussion

### 3.1 Computational Strategy for the Discovery of Novel Mode-of-Action Fungicides

A computational strategy was implemented to identify fungicides with a novel mechanism of action, based on two complementary blocks of ML and AI-driven QSAR models: one focused on parametrizing fungicidal activity and the other on API (Figures S1 and S2).

Twelve models were developed to predict fungicidal activity (Table 1), using LDA and ANN with molecular descriptors from AlvaDesc and Mordred. Despite moderate Wilks’ lambda values (0.5–0.7), the high *F*-values (≥10), and low *p*-values (≤0.00001) confirmed the strong discriminative ability of the models.

**Table 1:**
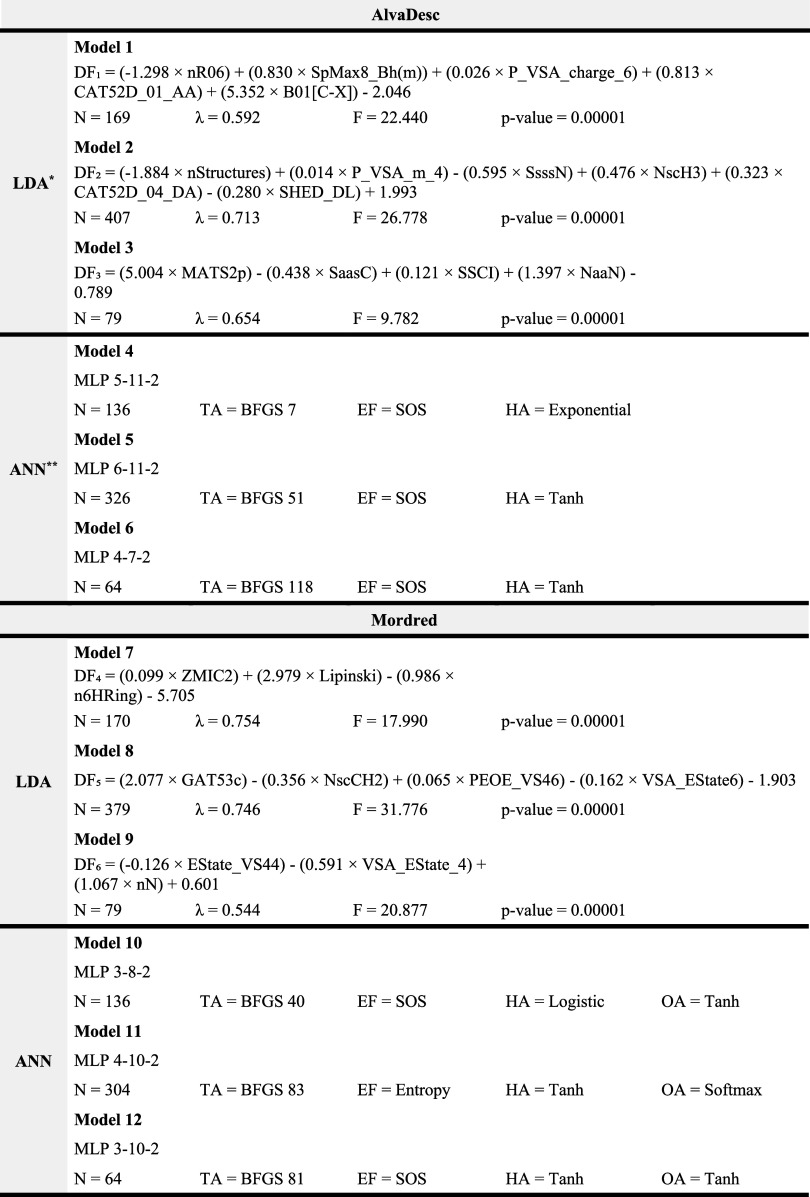
ML- and AI-Driven QSAR Models (LDA and ANN) for Identifying Novel Compounds with Fungicide Activity

* *N*: Number of compounds in the data set; λ (Wilks’ lambda); *F*: Fisher’s F-statistic.

** Each neural network in the system shares the same input dimensionality as the linear discriminant model, utilizing an identical number of input features (descriptors).

The models achieved an average classification accuracy of 81% for the training set in both the LDA and ANN models, demonstrating their capability to distinguish between active and inactive compounds (Table 2). LDA models 2, 7, and 9 showed higher sensitivity, while models 1, 3, and 8 exhibited greater specificity, patterns mirrored in ANN models (5 and 10 with higher sensitivity; 4, 6, 11, and 12 with higher specificity). The internal validation of ANN models yielded an average accuracy of 86%. For further detailed information about the internal validation process of the ANN models, please refer to the Supporting Information (Tables S13–S18). External validation of the models (data not shown) yielded an average correct classification rate of 66% in identifying compounds with fungicidal activity for all QSAR models; the models successfully classified 6 of 10 compounds with fungicidal activity, confirming their usefulness for prioritizing candidates during virtual screening.

**Table 2:**
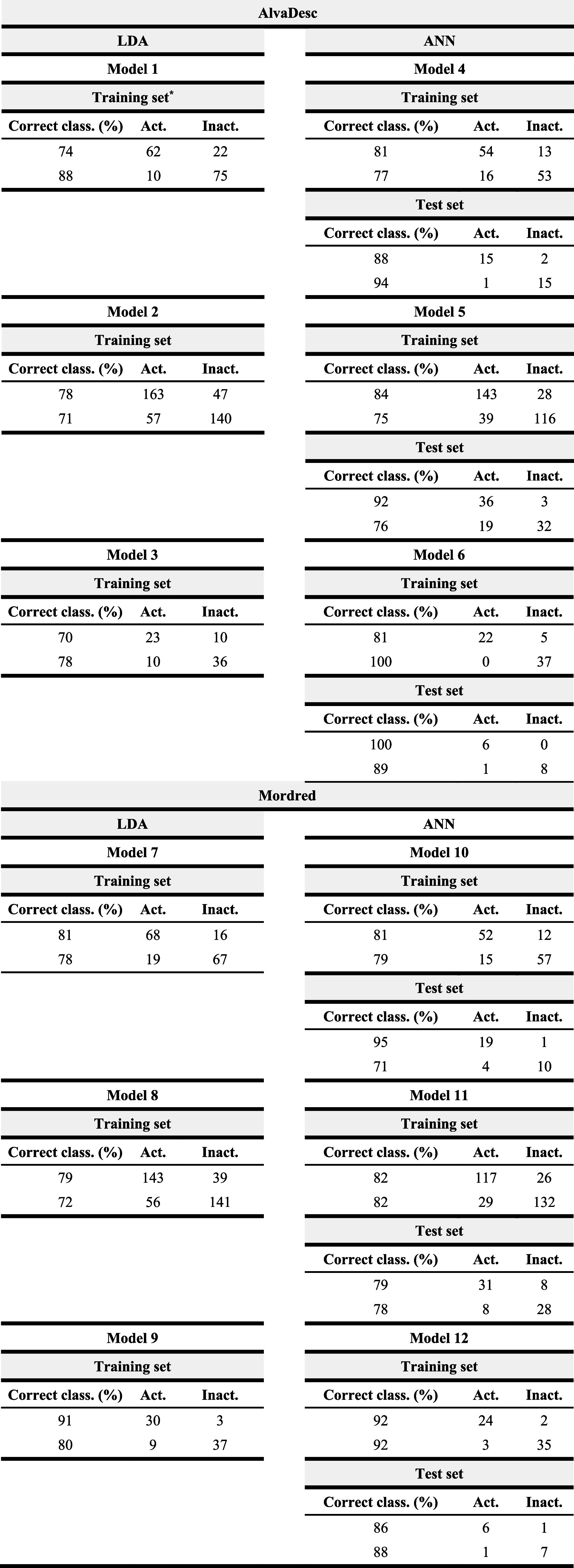
Classification Matrix for ML- and AI-Driven QSAR Models (LDA and ANN): Training and Internal Test Set Performance in Identifying Potential Fungicidal Compounds

* Activ.: active group; Inactiv.: inactive group; Class.: classification.

A second set of ML- and AI-driven QSAR models (Table 3) was developed to parametrize acid phosphatase inhibitory activity (Figure S2). Six models (two LDA and four ANN) were trained using descriptors from AlvaDesc and Mordred. For detailed information about the models, see the Supporting Information (Tables S19–24). Due to the limited data set size, only internal validation was performed for ANN-80 models, where 20% of the available data was reserved for testing (Tables S25–S26).

**Table 3:**
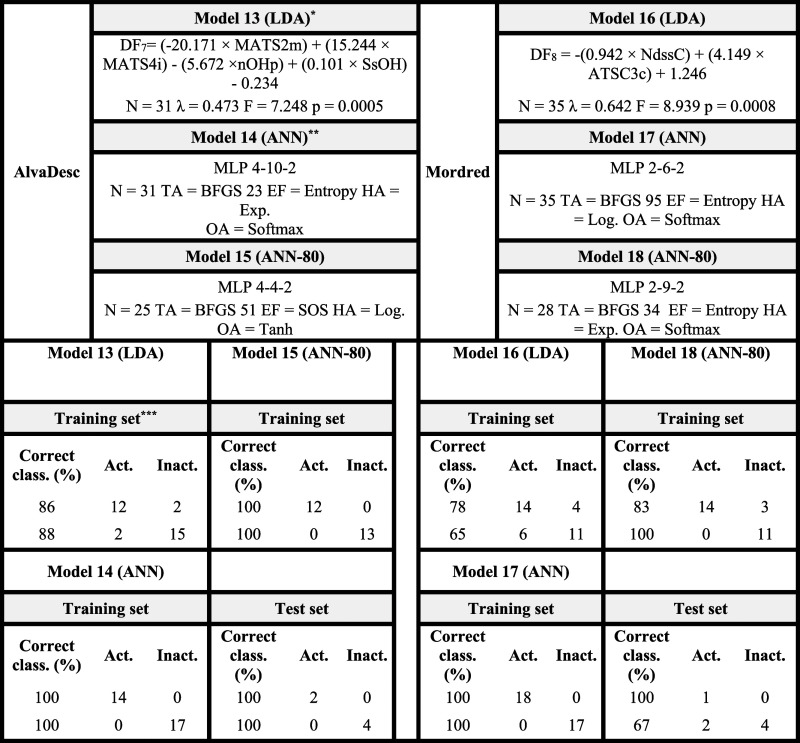
ML- and AI-driven QSAR Models for the Identification of Potential Phosphatase Acid Inhibitors[Table-fn t3fn4]

* *N*: Number of compounds in the data set; λ: Wilks’ lambda; *F*: Fisher’s F-statistic; *p*: *p*-value.

** Exp.: exponential; Log.:logistic.

*** Activ.: active group; Inactiv.: inactive group; Class.: classification.

a Classification matrix for training (LDA and ANN) and internal test set (ANN) results.

The AlvaDesc-based models demonstrated the highest performance, reaching up to 96–100% accuracy, while the Mordred models achieved 84–88%. Most models predicting API activity showed higher specificity than sensitivity, indicating a low probability of false positives in the virtual screening. These results validate the combined ML and AI approach as a robust tool for identifying potential FAPI.

Among the descriptors selected in the LDA models (Table 3), two variables stand out as particularly relevant (highest F values) for characterizing acid phosphatase inhibitory activity.

The first, MATS2m (Moran autocorrelation of lag 2 weighted by atomic mass), measures the correlation of atomic mass between atoms separated by two bonds (topological distance 2). Positive values of MATS2m indicate that atoms at this distance possess similar atomic masses (either high or low) as observed in compounds such as Gossypol and CID 6918295, representative of the active and inactive groups against API (Figure 2). Conversely, negative MATS2m values reflect greater differences in atomic mass between atoms at a topological distance of two, as found in Tunicamycin, L-(+)-tartaric acid, and CID 4671 (Figure 2).

**Figure 2:**
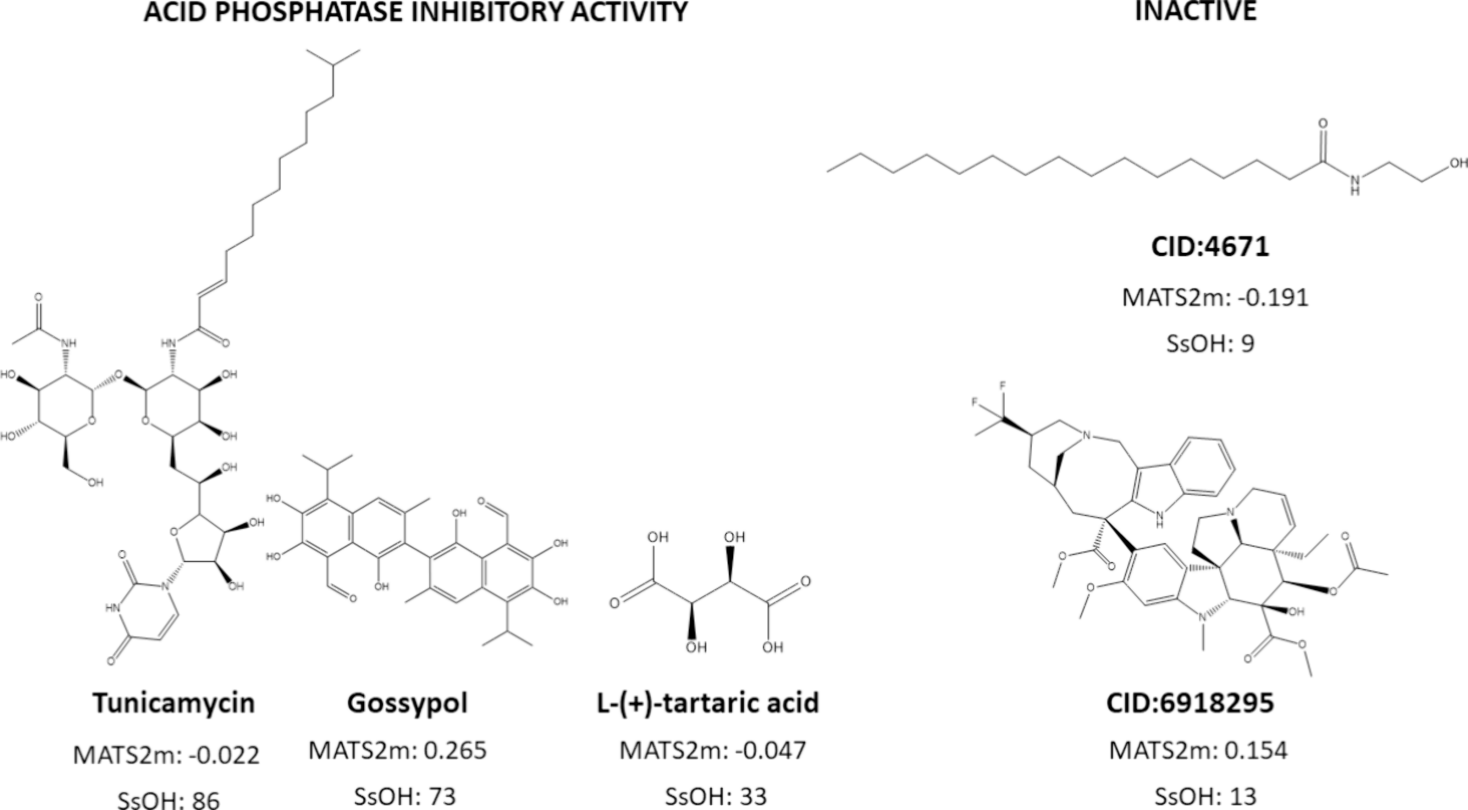
MATS2m and SsOH descriptor values for acid phosphatase inhibitors and inactive compounds in the training set (Model 13—Table S19).

The second relevant descriptor regarding acid phosphatase inhibitory activity, SsOH (Sum of sOH E-states), quantifies the sum of the electrotopological state (E-state) values for all oxygen atoms in hydroxyl groups (−OH) within a molecule. The E-state integrates both electronic contributions (atomic charge and polarization) and topological information (structural environment). High SsOH values denote molecules containing hydroxyl groups that are electronically accessible, structurally favorably positioned for interactions, capable of forming hydrogen bonds, and have minimal steric hindrance. As shown in Figure 2, compounds such as Tunicamycin, Gossypol, and L-tartaric acid exhibit elevated SsOH values (>30), whereas inactive molecules display moderate or low values (<30), indicating reduced presence or accessibility of hydroxyl groups for intermolecular interactions.

Considering the chemometrics-based patterns emerging from these two descriptors, molecules characterized by MATS2*m* < 0 and/or SsOH > 30 display a high probability of exhibiting inhibitory activity against acid phosphatase. Indeed, this pattern is observed in 13 of the 14 active compounds (Table S19). In contrast, only 4 of the 17 inactive compounds follow this pattern, indicating that active molecules are approximately three times more likely than inactive ones to satisfy these structural criteria.

### 3.2 Virtual Screening: First Selection

Three compound databases: eMolecules,^39^ Specs,^40^ and Sigma-Aldrich^41^ were screened to identify potential fungicides with a novel mode of action, specifically targeting API. The selection criteria included: (a) satisfying the activity thresholds of at least one fungicide model; (b) meeting the activity thresholds of at least one phosphatase activity model; (c) ensuring chemical diversity; (d) being commercially available; and (e) being affordably priced or having an acceptable quantity-to-price ratio. Based on these criteria, Table S27 in the Supporting Information presents the list of potential fungicides selected with a novel mode of action, the inhibition of acid phosphatase (FAPI).

Detailed information on the classification results from the machine learning and AI-driven QSAR models employed in the first virtual screening to select potential FAPI compounds is provided in the Supporting Information section (Tables S28–S30).

### 3.3 Experimental Assays: First Selection

#### 3.3.1 Determination of Fungicidal Activity by In Vitro and In Vivo Assays

Compounds identified by ML- and AI-driven QSAR models in the first virtual screening were initially tested for fungicidal activity on zucchini cotyledon discs against an isolate of *P. xanthii* 2086. In Figure 3A, the fungicidal effect of the compounds on cucurbit powdery mildew development is reported. The compounds that showed an efficacy above 50% in reducing disease symptoms compared to the negative control (water) were selected to perform the assays in planta using a dispersed inoculum of the same *P. xanthii* isolate, and only one concentration (5 mM) was determined after performing a battery of concentrations of the selected compounds in plant growth chambers (data not shown). Figure 3B shows the fungicidal effect of the compounds identified by ML- and AI-driven QSAR models on the development of cucurbit powdery mildew. Overall, the compounds showed comparable activity to that previously observed in the leaf disc assays. The highest fungicidal activity was obtained with FAPI-I-16 (cantharidin), with similar disease control that the one observed with the commercial fungicide fluopyram applied at the recommend field dose (100 μg/mL).

**Figure 3:**
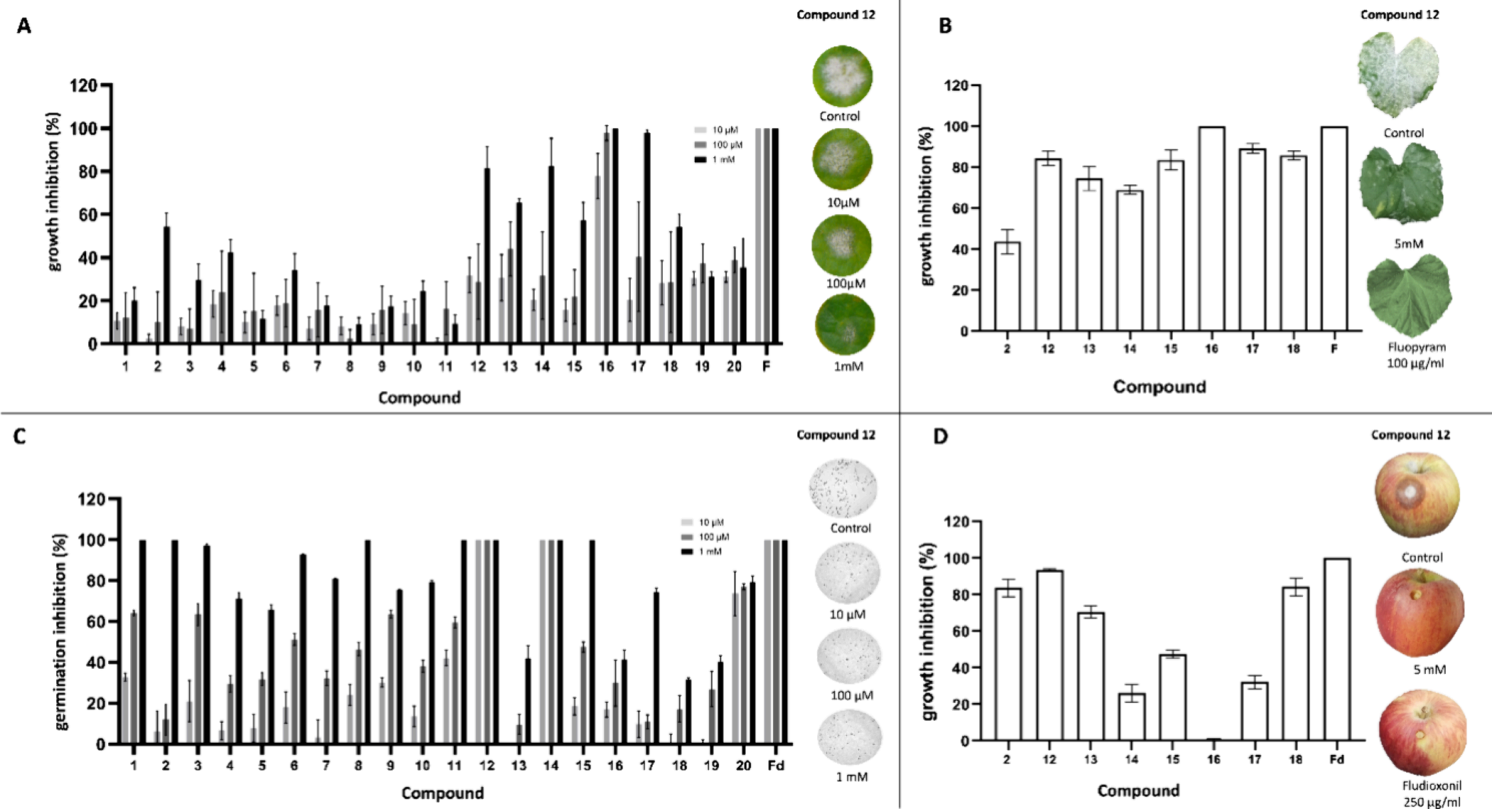
Fungicidal activity of first-screening compounds (FAPI-I) against two fungal pathogens: *P. xanthii*, on zucchini cotyledon discs (a) and leaf discs (b); and *B. cinerea*, via in vitro spore germination assays (c) and fruit assays (d). F: fluopyram, used as reference fungicide in assays (a,b); Fd: fludioxonil, used as reference fungicide in assays (c,d).

On the other hand, the compounds were further tested by spore germination and apple fruit assays to determine their fungicidal potential against the necrotrophic fungus *B. cinerea*. As shown in Figure 3C, most of the compounds showed outstanding spore suppression effects (above 50%) against *B. cinerea*, with FAPI-I-12, FAPI-I-14, and FAPI-I-20 achieving results comparable to the reference fungicide fludioxonil, even at the lowest tested concentration (10 μM). In addition, the same compounds tested in planta with the cucurbit powdery mildew were tested on apple fruit with *B. cinerea*. FAPI-I-2, FAPI-I-12, and FAPI-I-18 exhibited growth inhibition activity against B. *cinerea* (above 80%) comparable to that of the reference fungicide fludioxonil in this in vivo assay (Figure 3D).

From the experimental assays (Figure 3), two promising FAPI compounds: FAPI-I-12, which exhibits fungicidal activity against *B. cinerea*, and FAPI-I-16, which demonstrates fungicidal activity against *P. xanthii*. The latter’s fungicidal activity is further validated by its patent as cantharidin in 2018, part of a collection of traditional Chinese remedies. The patent highlights its fungicidal activity, supporting the experimental results obtained in our study.^48^

### 3.4 Optimizing the ML Strategy with Experimental Fungicidal Data to Identify FAPI

Following the experimental evaluation of compounds selected in the first virtual screening, new QSAR models were developed integrating these data to guide a second screening aimed at identifying potential FAPI. The models were trained by using the same methodological criteria (LDA and ANN with AlvaDesc and Mordred descriptors). These new models aimed to identify the chemo-mathematical patterns of molecules that exhibited fungicidal activity in at least one of the three experimental assays (leaf, plant, or fruit).

Table 4 summarizes the four ML- and AI-driven QSAR models generated (two LDA and two ANN), trained with the experimental data set. For the ANN models, 80% of the data were used for training and 20% for internal validation, while no external validation was possible due to the limited sample size. Internal validation achieved perfect classification accuracy (see Tables S31–S36 for details). All models exhibited higher specificity than sensitivity, indicating a low likelihood of false positives in the second virtual screening.

**Table 4:**
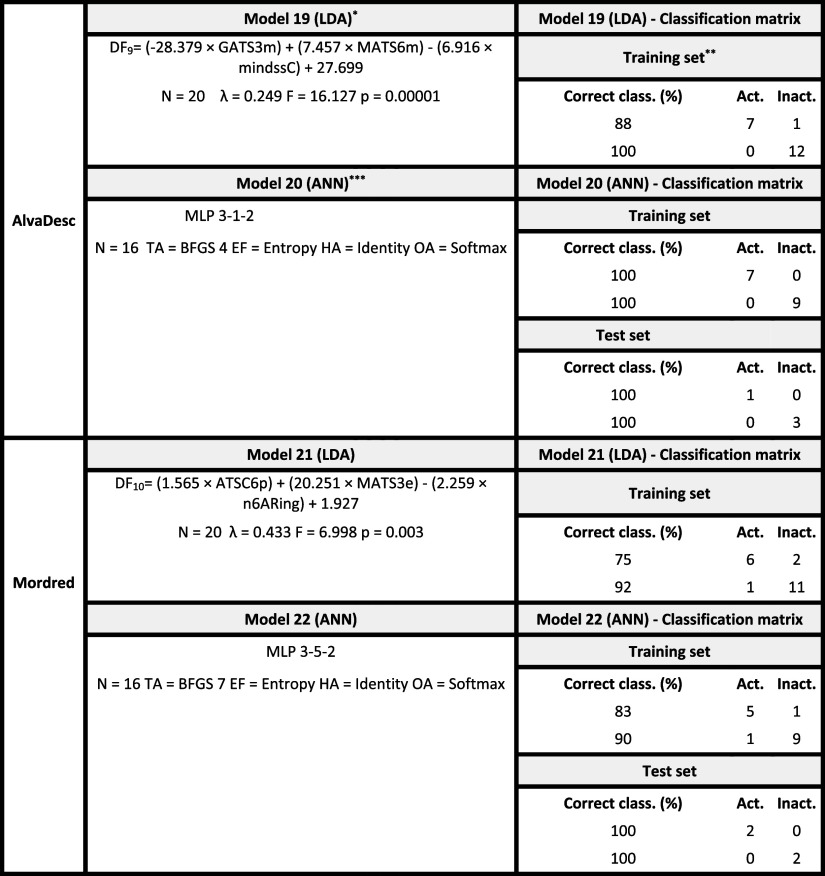
ML- and AI-driven QSAR Models for Identifying Fungicidal Activity Trained on Laboratory Experimental Data

* *N*: Number of compounds in the data set; λ: Wilks’ lambda; *F*: Fisher’s *F*-statistic; *p*: *p*-value.

** Each network (ANN) has the same input dimensionality as the corresponding linear discriminant model (LDA), and the weights for each descriptor are shared between the networks.

*** Activ.: active group; Inactiv.: inactive group; Class.: classification.

Among the most relevant descriptors for predicting the experimental fungicidal activity against *B. cinerea* and *P. xanthii*, GATS3m (AlvaDesc Model 19; *F* = 31.4) and MATS3se (Mordred Model 21; *F* = 14.0) emerged as key variables. Both are 2D autocorrelation descriptors that encode atomic properties at a topological distance of three bonds, emphasizing the critical role of this spatial feature in the fungicidal activity prediction.

Specifically, GATS3m represents the Geary autocorrelation of lag 3 weighted by the atomic mass. This descriptor increases as the relative mass differences between atoms separated by three bonds grow and as the number of such atom pairs rises. As shown in Figure 4, inactive compounds such as FAPI-I-06 and FAPI-I-07 exhibit higher GATS3m values, whereas active compounds such as FAPI-I-12 and FAPI-I-14 display lower values of this descriptor. Because GATS3m contributes to the discriminant function with a negative coefficient (Table 4), lower GATS3m values correspond to less negative DF scores and, consequently, a higher probability of being classified as fungicidal (DF > 0). Therefore, molecules showing smaller mass differences between atoms separated by three bonds and a greater number of such atom pairs tend to present lower GATS3m values and, according to Model 19 (Table 4), an increased likelihood of exhibiting fungicidal activity.

**Figure 4:**
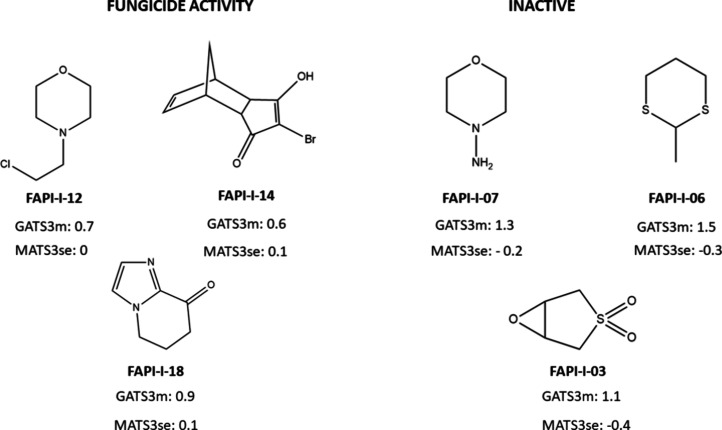
GATS3m and MATS3se descriptor values for active and inactive compounds in the training set (Model 19—Table S30; Model 21—Table S32).

Analyzing the most significant descriptor from Mordred Model 21, MATS3se, 2D Moran coefficient of lag 3 weighted by Sanderson electronegativity contributes to the equation with a significance of 14 (F-value); suggests that the presence of more electronegativity atoms at a topological distance of 3, as indicated by the MATS3se descriptor, is a key factor in the development of fungicidal compounds.

Regarding the most significant descriptor in Mordred Model 21, MATS3se (Moran autocorrelation of lag 3 weighted by Sanderson electronegativity) also highlights the importance of topological features at three-bond distances. This descriptor suggests that the presence of more electronegative atoms at this distance is a key factor underlying the fungicidal behavior. As shown in Figure 4, inactive compounds such as FAPI-I-03, FAPI-I-07, and FAPI-I-06 exhibit MATS3se lower values of this index, whereas active compounds such as FAPI-I-12, FAPI-I-14, and FAPI-I-18 show higher values. These higher values are associated with the presence of more electronegative atoms (e.g., Cl or F) at a topological distance of three, a structural characteristic positively correlated with fungicidal activity, as illustrated by the fluorinated fungicides Fluopyram and Fludioxonil. This trend is consistent with the positive coefficient of MATS3se (Table 4), as the higher the MATS3se value, the more positive the DF score, and consequently, the greater the probability of being classified as a fungicidal compound (DF > 0).

Overall, these findings underscore the relevance of specific atomic arrangements and electronic features at a topological distance of three bonds in determining the fungicidal potential.

### 3.5 Virtual Screening: Second Selection

Three database compounds were screened: Specs, eMolecules, and Sigma-Aldrich. The overall established criteria for the selection of potential FAPI were (a) meeting the activity criteria established by at least two fungicide models, (b) meeting the activity criteria established by at least one phosphatase activity model, (c) guarantee chemical diversity, (d) being commercially available, and (e) being affordably priced or having an acceptable quantity-to-price ratio. Based on these criteria, Table S37 presents the list of potential fungicides selected with a novel mode of action, the inhibition of acid phosphatase. Detailed classification results from the QSAR models for the selected compounds in the second virtual screening campaign are available in the Supporting Information (Tables S38–S41).

### 3.6 Experimental Assays: Second Selection

#### 3.6.1 Determination of Fungicidal Activity by In Vitro and In Vivo Assays

The new compounds identified by a second round of virtual screening strategy (FAPI-II), were tested on zucchini cotyledon discs and in planta against the isolate of *P. xanthii* 2086. In Figure 5A, the fungicidal effect of the compounds on cucurbit powdery mildew development is reported. The compounds that showed an efficacy above 40% in reducing disease symptoms compared to the negative control (water) were selected to perform the assays in planta using a dispersed inoculum of the same *P. xanthii* isolate at a concentration of 5 mM. Figure 5B shows the fungicidal effect of the compounds. Overall, the compounds exhibited comparable fungicidal activity against *P. xanthii* in both plant and leaf disc assays, with FAPI-II-2, FAPI-II-5, FAPI-II-9, and FAPI-II-13 standing out for their fungicidal capacity, achieving approximately 60% fungal growth inhibition.

**Figure 5:**
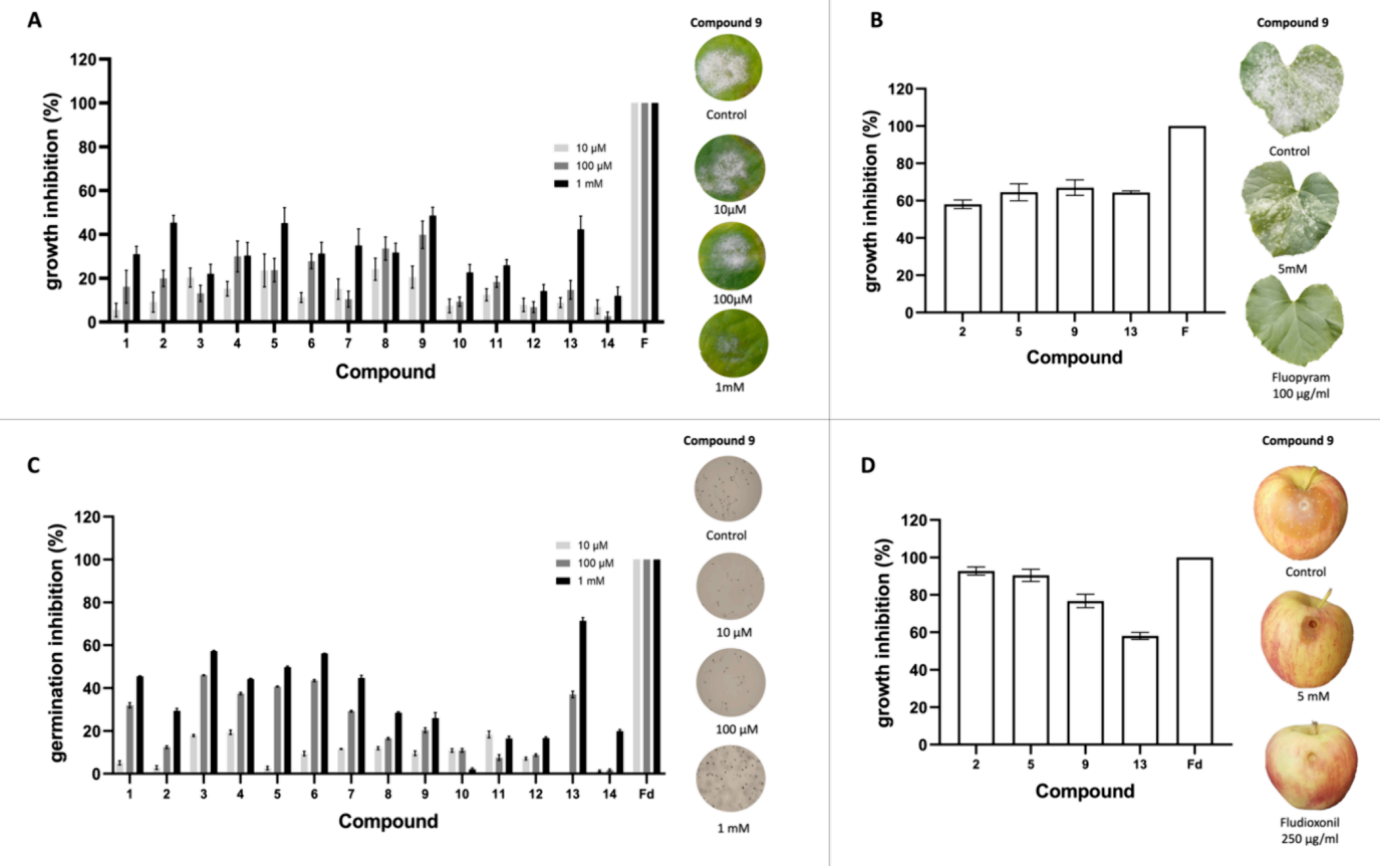
Fungicidal activity of second-screening compounds (FAPI-II) against two fungal pathogens: *P. xanthii*, on zucchini cotyledon discs (a) and leaf discs (b); and *B. cinerea*, via in vitro spore germination assays (c) and fruit assays (d). F: fluopyram, used as reference fungicide in assays (a,b); Fd: fludioxonil, used as reference fungicide in assays (c,d).

Like what was explained above, the compounds were further tested by spore germination and apple fruit assays to determine their fungicidal potential against *B. cinerea*. As shown in Figure 5C, seven compounds showed spore suppression effects of >40% against *B. cinerea*. On the other hand, the same compounds tested in planta with the cucurbit powdery mildew were tested on apple fruit (Figure 5D) with *B. cinerea* obtaining a high level of growth inhibition (more than 70%) with the compounds FAPI-II-02, FAPI-II-05, and FAPI-II-09 showing comparable fungicide effect as that of the reference fungicide fludioxonil.

### 3.7 Molecular Docking

To validate the predicted mechanism of action of the new **FAPI** compounds, molecular docking studies were performed to analyze their interactions with acid phosphatase enzymes. This approach aimed to support the proposed mechanism involving API as a novel fungicidal target.^49^

Docking analyses were carried out using the crystallized structure of *Aspergillus niger* acid phosphatase (PDB: 1QFX) and a homology model for *P. xanthii* (model ID: 15569). Phytic acid, the natural substrate of acid phosphatase, was used as a reference for comparison.

For *A. niger* (1QFX), phytic acid exhibited a docking score of −2.356 kcal/mol (Table S42). Most FAPI compounds showed more favorable scores, interacting with key catalytic residues (Arg62, His63, Arg66, Asp75, Arg156, Glu272, His318, and Asp319). The best-performing compound, FAPI-I-13 (−4.718 kcal/mol), formed stable hydrogen and ionic bonds with Arg62, Arg66, Arg156, and Asp319 (Figure 6). Extended information regarding the molecular docking study can be found in the Supporting Information.

**Figure 6:**
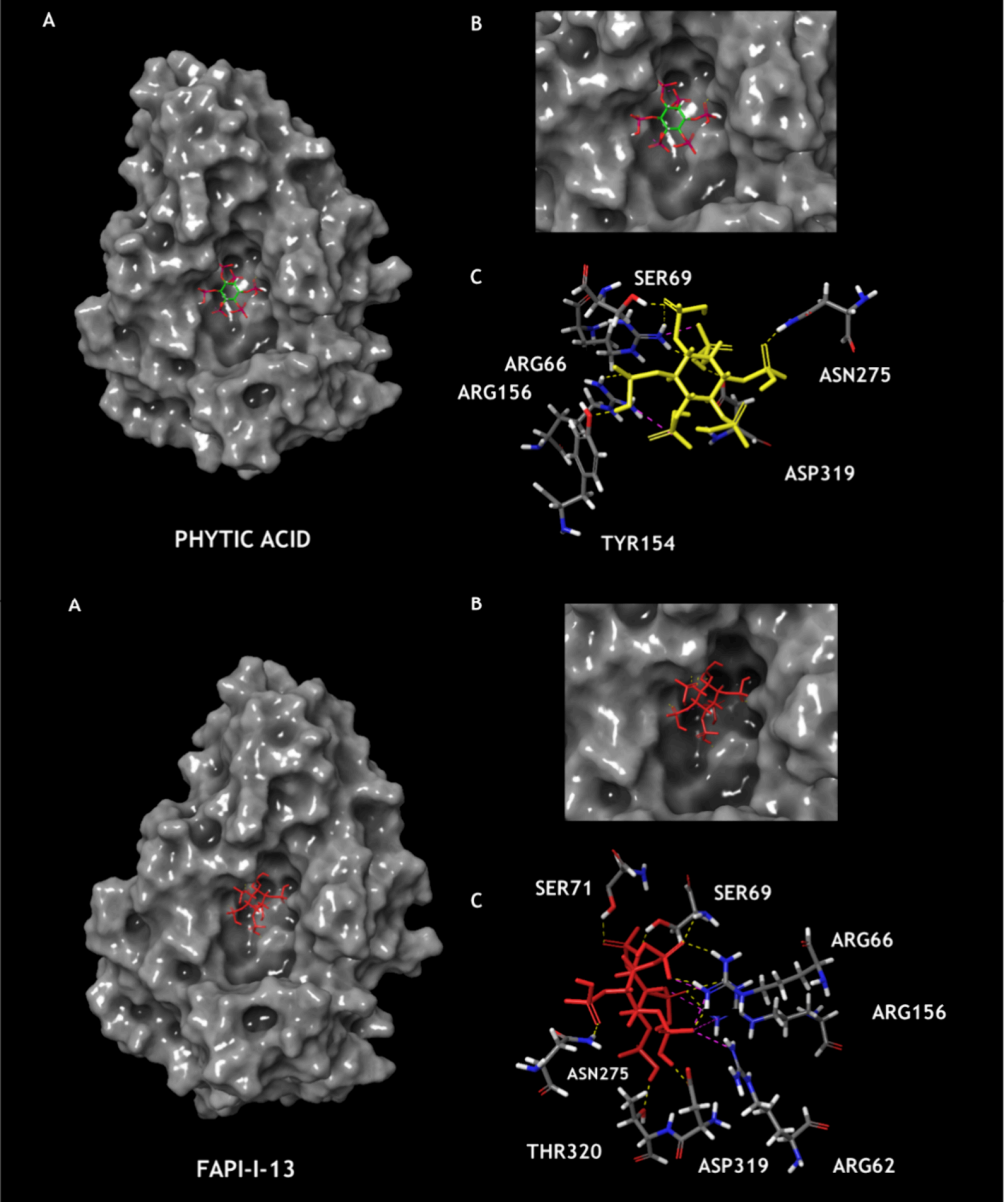
Predicted binding modes of phytic acid (top) and FAPI-I-13 (bottom) within the catalytic site of acid phosphatase (PDB ID: 1QFX). (A) Surface representation of acid phosphatase. (B) Surface representation of the binding pocket. (C) Amino acid residues involved in ligand binding with dashed lines indicating hydrogen bonds (yellow) and ionic interactions (pink).

In this study, a combined computational–experimental strategy was employed to identify novel fungicides acting through acid phosphatase inhibition (FAPI), addressing the growing challenge of fungicide resistance. Compounds such as FAPI-I-12, FAPI-I-16, FAPI-II-5, and FAPI-II-13 have emerged as promising candidates. Computational analyses support API as their potential mechanism of action, although further enzymatic validation is needed.

Biological assays revealed that FAPI-I-12 and FAPI-II-5 were active against *B. cinerea*, while FAPI-I-16, FAPI-II-5, and FAPI-II-13 exhibited activity against *P. xanthii*, indicating their potential to manage resistance and provide durable disease control in crops, such as cucumber, strawberry, grape, and tomato.

The two-step virtual screening strategy, integrating ML- and AI-driven QSAR models with experimental data, proved to be highly effective for prioritizing active candidates. By targeting acid phosphatases, an underexplored enzymatic pathway, this approach offers a framework for developing next-generation fungicides with enhanced specificity and reduced risk of cross-resistance. Future studies should focus on enzymatic validation, SAR-driven optimization, and formulation development to confirm and enhance the FAPI performance under field conditions.

In conclusion, this integrative computational–experimental pipeline accelerates fungicide discovery and underscores the value of targeting novel biochemical pathways for sustainable crop protection.

## Supplementary Material



## Notes

Comprehensive details on the ML- and AI-driven QSAR models employed in this study for the identification of novel FAPI compounds (Tables S1–S42 and Figures S1–S3) and extended and detailed information regarding the Molecular Docking Study (PDF)

## Abbreviations

AI: artificial intelligence
ANN: artificial neural networks
API: acid phosphatase inhibition
Aps: acid phosphatases
ATM-HIGS: *Agrobacterium tumefaciens*-mediated host-induced gene silencing
FAPI: fungicides targeting acid phosphatase inhibition
FRAC: Fungicide Resistance Action Committee
LDA: linear discriminant analysis
ML: machine learning
QSAR: quantitative structure–activity relationship
