# Supplementary material for: Machine Learning- and AI-Driven QSAR Models for the Discovery of Novel Potential Fungicides: FAPI (Fungicide Targeting Acid Phosphatase Inhibition)
Source: J Agric Food Chem. 2025 Dec 24;74(1):247–58. doi: 10.1021/acs.jafc.5c07939 (PMC12814334; doi:10.1021/acs.jafc.5c07939)
Supplement: Supplementary file 1 [file jf5c07939_si_001.pdf]

Supporting Information

Machine Learning and AI-driven QSAR models for the Discovery of Novel Potential Fungicides: FAPI (Fungicide Targeting Acid Phosphatase Inhibition)

Gálvez-Llompart, María;[a] Zanni,Riccardo;[b] Morales, Yandira;[c] Polonio, Álvaro; [c] Fernández-Ortuño, Dolores; [c] Pérez-García, Alejandro; [c] Pérez-Giménez, Facundo[b]

[a] Department of Preventive Medicine and Public Health, Food Science, Toxicology and Forensic Medicine, Faculty of Pharmacy, University of Valencia, 46100 Burjassot, Valencia, Spain

[b] Department of Physical Chemistry, University of Valencia, 46100 Burjassot, Valencia, Spain

[c] Department of Microbiology, Faculty of Science, Instituto de Hortofruticultura Subtropical y Mediterránea La Mayora, IHSM-UMA-CSIC, University of Málaga, 29071, Málaga, Spain

*Correspondence:

Maria Galvez-Llompart: maria.galvez@uv.es

Table of Contents

Table S1. Classification and probability of classification for training set compounds in Model 1 (LDA-AlvaDesc) Trained with WIPO data, focusing on Fungicide activity prediction…………4

Table S2. Classification and probability of classification for training set compounds in Model 2 (LDA-AlvaDesc) Trained with FRAC-WIPO data, focusing on Fungicide activity prediction….9

Table S3. Classification and probability of classification for training set compounds in Model 3 (LDA-AlvaDesc) Trained with Pubchem-WIPO data, focusing on Fungicide activity prediction……………………………………………………………………………..18

Table S4. Classification and probability of classification for training set compounds in Model 4 (ANN-AlvaDesc) Trained with WIPO data, focusing on Fungicide activity prediction…………20

Table S5. Classification and probability of classification for training set compounds in Model 5 (ANN-AlvaDesc) Trained with FRAC-WIPO data, focusing on Fungicide activity prediction…24

Table S6. Classification and probability of classification for training set compounds in Model 6 (ANN-AlvaDesc) Trained with Pubchem-WIPO data, focusing on Fungicide activity prediction……………………………………………………………………………..32

Table S7. Classification and probability of classification for training set compounds in Model 7 (LDA-Mordred) Trained with WIPO data, focusing on Fungicide activity prediction………….34

Table S8. Classification and probability of classification for training set compounds in Model 8 (LDA-Mordred) Trained with FRAC-WIPO data, focusing on Fungicide activity prediction…38

Table S9. Classification and probability of classification for training set compounds in Model 9 (LDA-Mordred) Trained with Pubchem-WIPO data, focusing on Fungicide activity prediction..47

Table S10. Classification and probability of classification for training set compounds in Model 10 (ANN- Mordred) Trained with WIPO data, focusing on Fungicide activity prediction……..49

Table S11. Classification and probability of classification for training set compounds in Model 11 (ANN- Mordred) Trained with FRAC-WIPO data, focusing on Fungicide activity prediction……………………………………………………………………………..53

Table S12. Classification and probability of classification for training set compounds in Model 12 (ANN- Mordred) Trained with Pubchem-WIPO data, focusing on Fungicide activity prediction……………………………………………………………………………..60

Table S13. Classification and probability of classification for internal test set compounds in Model 4 (ANN-AlvaDesc) Trained with WIPO data, focusing on Fungicide activity prediction……………………………………………………………………………..62

Table S14. Classification and probability of classification for internal test set compounds in Model 5 (ANN-AlvaDesc) Trained with FRAC-WIPO data, focusing on Fungicide activity prediction……………………………………………………………………………..63

Table S15. Classification and probability of classification for internal test set compounds in Model 6 (ANN-AlvaDesc) Trained with Pubchem-WIPO data, focusing on Fungicide activity prediction……………………………………………………………………………..65

Table S16. Classification and probability of classification for internal test set compounds in Model 10 (ANN- Mordred) Trained with WIPO data, focusing on Fungicide activity prediction……………………………………………………………………………..66

Table S17. Classification and probability of classification for internal test set compounds in Model 11 (ANN- Mordred) Trained with FRAC-WIPO data, focusing on Fungicide activity prediction……………………………………………………………………………67

Table S18. Classification and probability of classification for internal test set compounds in Model 12 (ANN- Mordred) Trained with Pubchem-WIPO data, focusing on Fungicide activity prediction……………………………………………………………………………………..69

Table S19. Classification and probability of classification for training set compounds in Model 13 (LDA-AlvaDesc), focusing on Acid Phosphatase Inhibitory activity prediction……………70

Table S20. Classification and probability of classification for training set compounds in Model 14 (ANN-AlvaDesc), focusing on Acid Phosphatase Inhibitory activity prediction……………71

Table S21. Classification and probability of classification for training set compounds in Model 15 (ANN80-AlvaDesc), focusing on Acid Phosphatase Inhibitory activity prediction………….72

Table S22. Classification and probability of classification for training set compounds in Model 16 (LDA-Mordred), focusing on Acid Phosphatase Inhibitory activity prediction……………..73

Table S23. Classification and probability of classification for training set compounds in Model 17 (ANN-Mordred), focusing on Acid Phosphatase Inhibitory activity prediction……………74

Table S24. Classification and probability of classification for training set compounds in Model 18 (ANN80-Mordred), focusing on Acid Phosphatase Inhibitory activity prediction………….75

Table S25. Classification and probability of classification for internal test set compounds in Model 15 (ANN80-AlvaDesc), focusing on Acid Phosphatase Inhibitory activity prediction….76

Table S26. Classification and probability of classification for internal test set compounds in Model 18 (ANN80-Mordred), focusing on Acid Phosphatase Inhibitory activity prediction……76

Table S27. First virtual screening selection of potential **F**ungicides targeting **A**cid **P**hosphatase **I**nhibition) (**FAPI**)………………………………………………………………………………77

Table S28. Classification and Probability of Classification for the First Virtual Screening Focused on Fungicide Activity Prediction as Part of the *In Silico* Strategy to Identify Novel FAPI Compounds Using AlvaDesc QSAR Models…………………………………………………80

Table S29. Classification and Probability of Classification for the First Virtual Screening Focused on Fungicide Activity Prediction as Part of the *In Silico* Strategy to Identify Novel FAPI Compounds Using Mordred QSAR Models…………………………………………………..81

Table S30. Classification and Probability of Classification for the First Virtual Screening Focused on Acid Phosphatase Inhibitory activity prediction as Part of the *In Silico* Strategy to Identify Novel FAPI Compounds Using AlvaDesc and Mordred QSAR Models………………………82

Table S31. Classification and probability of classification for training set compounds in Model 19 (LDA-AlvaDesc) Trained with experimental fungicide activity data, focusing on Fungicide activity prediction…………………………………………………………………………………83

Table S32. Classification and probability of classification for training set compounds in Model 20 (ANN-AlvaDesc) Trained with experimental fungicide activity data, focusing on Fungicide activity prediction…………………………………………………………………………………84

Table S33. Classification and probability of classification for training set compounds in Model 21 (LDA-Mordred) Trained with experimental fungicide activity data, focusing on Fungicide activity prediction…………………………………………………………………………………85

Table S34. Classification and probability of classification for training set compounds in Model 22 (ANN-Mordred) Trained with experimental fungicide activity data, focusing on Fungicide activity prediction…………………………………………………………………………………86

Table S35. Classification and probability of classification for internal test set compounds in Model 20 (ANN-AlvaDesc) trained with experimental fungicide activity data, focusing on Fungicide activity prediction……………………………………………………………………87

Table S36. Classification and probability of classification for internal test set compounds in Model 22 (ANN-Mordred) Trained with experimental fungicide activity data, focusing on Fungicide activity prediction……………………………………………………………………87

Table S37. Second screening selection of potential Fungicides targeting Acid Phosphatase Inhibition (FAPI-II)……………………………………………………………………………..88

Table S38. Classification and Probability of Classification for the Second Virtual Screening Focused on Fungicide Activity Prediction as Part of the *In Silico* Strategy to Identify Novel FAPI Compounds Using AlvaDesc QSAR Models…………………………………………………91

Table S39. Classification and Probability of Classification for the Second Virtual Screening Focused on Fungicide Activity Prediction as Part of the *In Silico* Strategy to Identify Novel FAPI Compounds Using Mordred QSAR Models…………………………………………………92

Table S40. Classification and Probability of Classification for the Second Virtual Screening Focused on Acid Phosphatase Inhibitory activity prediction as Part of the *In Silico* Strategy to Identify Novel FAPI Compounds Using AlvaDesc and Mordred QSAR Models………………93

Table S41. Classification and Probability of Classification for the Second Virtual Screening Focused on experimental fungicide activity prediction as Part of the *In Silico* Strategy to Identify Novel FAPI Compounds Using AlvaDesc and Mordred QSAR Models………………………94

Table S42. Docking score values (kcal/mol) and amino acid interactions between acid phosphatase from *Aspergillus niger* (PDB: 1QFX) and *Podosphaera xanthii* (homology model 15569)……………………………………………………………………………………...95

Figure S1. Machine Learning and AI-Driven QSAR Modeling strategy for identifying novel compounds with fungicide activity…………………………………………………………...97

Figure S2. Machine Learning and AI-Driven QSAR Modeling strategy for the discovery of novel acid phosphatase inhibitors……………………………………………………………………...98

Figure S3. Machine Learning and AI-Driven QSAR Modeling strategy for identifying novel compounds with fungicide activity based on UMA's experimental results…………………..…99

Molecular docking……………………………………………………………………………100

Table S1. Classification and probability of classification for training set compounds in Model 1 (LDA-AlvaDesc) trained with WIPO data, focusing on Fungicide activity prediction.

CID	nR06	SpMax8_Bh(m)	P_VSA_charge_6	CATS2D_01_AA	B01[C-X]	DF	Cl.	C.L.
**Active group**								
23690429	1	0.882	0.000	0	0	-2.612	NF	0.932
54682462	1	1.331	0.000	0	0	-2.239	NF	0.904
9257	0	0.000	0.000	0	0	-2.046	NF	0.886
24462	0	0.000	0.000	0	0	-2.046	NF	0.886
30154	0	0.000	0.000	0	0	-2.046	NF	0.886
5366415	0	0.000	0.000	0	0	-2.046	NF	0.886
23663539	1	1.843	0.000	0	0	-1.815	NF	0.860
213032	3	2.693	75.810	0	0	-1.734	NF	0.847
7156993	0	0.826	0.000	0	0	-1.360	NF	0.796
5430	1	1.802	18.952	0	0	-1.356	NF	0.794
18771	3	2.510	102.344	0	0	-1.196	NF	0.762
50367	2	2.518	60.648	0	0	-0.975	NF	0.722
10935908	2	2.689	56.857	0	0	-0.932	NF	0.714
10788	1	0.608	77.412	0	0	-0.827	NF	0.690
11048796	4	2.916	96.453	2	0	-0.684	NF	0.658
3032581	0	1.651	0.000	0	0	-0.676	NF	0.663
3037	2	2.431	78.297	0	0	-0.588	NF	0.637
3032792	1	2.819	18.952	0	0	-0.511	NF	0.624
28780	1	2.660	30.312	0	0	-0.348	NF	0.584
17581	1	1.351	78.297	0	0	-0.187	NF	0.540
5634	0	1.787	19.614	0	0	-0.053	NF	0.512
91699	1	2.501	47.974	0	0	-0.020	NF	0.501
33112	0	2.361	8.825	0	0	0.143	F	0.536
86173	2	2.687	78.297	1	0	0.437	F	0.614
11292824	2	2.989	101.639	0	0	0.481	F	0.626
6112114	2	2.689	85.734	1	0	0.632	F	0.659
6451142	1	2.772	67.577	0	0	0.714	F	0.676
6437379	1	2.530	75.810	0	0	0.727	F	0.680
135083	2	2.562	0.000	4	0	0.736	F	0.676
17110	0	2.438	31.636	0	0	0.800	F	0.692
1493	1	1.331	0.000	4	0	1.013	F	0.733
9492	1	1.331	0.000	4	0	1.013	F	0.733
4921319	1	1.331	0.000	4	0	1.013	F	0.733
6422843	2	2.749	67.577	2	0	1.023	F	0.740
39676	1	2.323	94.064	0	0	1.030	F	0.743
122087	1	2.714	87.122	0	0	1.174	F	0.769
86132	1	2.400	66.926	1	0	1.201	F	0.773
39385	1	2.499	65.624	1	0	1.249	F	0.781
41368	1	2.499	65.624	1	0	1.249	F	0.781
6191	1	1.331	9.476	4	0	1.259	F	0.779
7744	0	2.730	44.309	0	0	1.372	F	0.800
7430	1	1.331	78.297	2	0	1.423	F	0.810
92200	2	2.581	154.823	0	0	1.525	F	0.829

6327657	3	2.554	0.000	0	1	1.532	F	0.822
7223	0	2.932	44.309	0	0	1.540	F	0.826
11159	0	2.933	44.309	0	0	1.541	F	0.826
2730	1	2.333	117.446	0	0	1.646	F	0.844
59649244	2	2.842	124.882	1	0	1.777	F	0.860
22321033	1	1.331	0.000	5	0	1.826	F	0.861
5455	0	1.939	90.384	0	0	1.913	F	0.875
6950	1	1.979	17.650	4	0	2.010	F	0.882
11664966	2	2.838	105.133	2	0	2.073	F	0.892
213016	2	2.838	105.133	2	0	2.073	F	0.892
9578570	2	2.838	105.133	2	0	2.073	F	0.892
66461	1	2.382	101.668	1	0	2.089	F	0.893
15910	1	1.708	156.595	0	0	2.145	F	0.900
8607	1	2.491	136.398	0	0	2.270	F	0.910
7511	1	0.341	0.000	0	1	2.291	F	0.908
8606	1	2.561	136.398	0	0	2.328	F	0.915
17776	1	2.446	17.650	4	0	2.397	F	0.917
5460680	1	0.496	0.000	0	1	2.420	F	0.918
17432	0	1.807	117.446	0	0	2.508	F	0.927
14994	1	2.444	26.476	4	0	2.625	F	0.933
25202562	1	1.039	0.000	0	1	2.870	F	0.946
1730	1	1.039	0.000	0	1	2.870	F	0.946
7627	1	1.039	0.000	0	1	2.870	F	0.946
14309	1	1.039	0.000	0	1	2.870	F	0.946
65258	1	1.039	0.000	0	1	2.870	F	0.946
3647458	1	1.039	0.000	0	1	2.870	F	0.946
10598	2	2.687	0.000	0	1	2.940	F	0.950
11953884	1	3.541	131.365	0	0	3.010	F	0.955
16682983	1	1.286	0.000	0	1	3.076	F	0.956
16682942	0	0.083	0.000	0	1	3.375	F	0.967
12318	0	0.083	0.000	0	1	3.375	F	0.967
56840815	0	0.083	0.000	0	1	3.375	F	0.967
124962	1	3.589	20.185	4	0	3.412	F	0.968
16682936	0	0.820	0.000	0	1	3.987	F	0.982
45380430	2	3.058	173.954	2	0	4.045	F	0.984
6720	1	1.603	195.743	2	0	4.702	F	0.992
16684215	1	1.292	0.000	2	1	4.707	F	0.991
90545	1	1.331	0.000	2	1	4.739	F	0.991
16682924	1	1.039	0.000	3	1	5.309	F	0.995
11486133	1	2.911	115.504	4	0	5.327	F	0.995
72980153	1	2.911	115.504	4	0	5.327	F	0.995
**Inactive group**								
10016922	4	2.581	9.476	0	0	-4.850	NF	0.992
115223	4	2.674	28.390	0	0	-4.280	NF	0.986
1720	3	1.789	18.952	0	0	-3.962	NF	0.981
73801	3	2.660	0.000	0	0	-3.732	NF	0.977
1727	1	0.083	0.000	0	0	-3.275	NF	0.964

100516	3	2.969	18.952	0	0	-2.983	NF	0.952
232487	1	0.445	0.000	0	0	-2.975	NF	0.951
1018	1	0.155	9.476	0	0	-2.969	NF	0.951
457	1	0.620	0.000	0	0	-2.829	NF	0.944
82178	3	2.517	41.091	0	0	-2.783	NF	0.941
14454445	3	2.916	28.429	0	0	-2.780	NF	0.941
154257	3	3.057	24.022	0	0	-2.778	NF	0.941
996	1	0.000	28.429	0	0	-2.605	NF	0.931
216239	3	2.861	39.149	0	0	-2.548	NF	0.927
10047015	3	2.812	9.476	1	0	-2.546	NF	0.927
114681	1	0.710	8.825	0	0	-2.526	NF	0.926
130229	2	2.629	0.000	0	0	-2.460	NF	0.921
219095	3	2.890	48.625	0	0	-2.277	NF	0.906
77139	1	1.331	0.000	0	0	-2.239	NF	0.904
133128	2	2.898	0.000	0	0	-2.236	NF	0.903
1050	1	1.418	0.000	0	0	-2.167	NF	0.897
227	1	0.838	18.952	0	0	-2.156	NF	0.896
168884	3	2.866	54.985	0	0	-2.132	NF	0.892
14777	0	0.000	0.000	0	0	-2.046	NF	0.886
10176082	0	0.000	0.000	0	0	-2.046	NF	0.886
78165	1	1.596	0.000	0	0	-2.019	NF	0.883
127151	3	2.695	66.334	0	0	-1.979	NF	0.876
1646	0	0.083	0.000	0	0	-1.977	NF	0.878
10313100	0	0.083	0.000	0	0	-1.977	NF	0.878
194233	0	0.083	0.000	0	0	-1.977	NF	0.878
10903489	0	0.083	0.000	0	0	-1.977	NF	0.878
11033	1	1.667	0.000	0	0	-1.961	NF	0.877
1140	1	0.083	50.567	0	0	-1.960	NF	0.875
1456	2	1.087	38.352	1	0	-1.929	NF	0.872
101209015	1	1.283	14.243	0	0	-1.908	NF	0.870
10783	0	0.348	0.000	0	0	-1.757	NF	0.853
112056	1	1.368	18.952	0	0	-1.716	NF	0.847
123983	2	2.687	28.429	0	0	-1.673	NF	0.841
72300	2	2.432	37.905	0	0	-1.638	NF	0.836
222284	3	3.049	68.148	0	0	-1.638	NF	0.834
78782	1	0.841	39.149	0	0	-1.628	NF	0.834
170317	3	2.852	77.054	0	0	-1.569	NF	0.824
89124	0	0.083	15.767	0	0	-1.567	NF	0.827
168045	1	2.164	0.000	0	0	-1.548	NF	0.825
1864	1	2.266	0.000	0	0	-1.464	NF	0.812
15706387	2	2.308	49.916	0	0	-1.428	NF	0.804
999	1	0.839	47.381	0	0	-1.416	NF	0.802
10350985	6	3.718	0.000	0	1	-1.396	NF	0.802
93379	2	2.173	67.577	0	0	-1.081	NF	0.743
97663	0	1.192	0.000	0	0	-1.057	NF	0.742
119569	1	2.503	8.825	0	0	-1.037	NF	0.738
10419733	0	1.234	0.000	0	0	-1.022	NF	0.735

76962	0	0.083	8.825	1	0	-0.935	NF	0.717
92411	2	2.838	56.799	0	0	-0.810	NF	0.688
159247	0	1.224	9.476	0	0	-0.783	NF	0.686
2708	1	2.498	18.952	0	0	-0.778	NF	0.684
101815861	0	1.585	0.000	0	0	-0.731	NF	0.675
2789	2	2.613	67.577	0	0	-0.716	NF	0.667
11001318	2	2.337	78.297	0	0	-0.666	NF	0.655
15532	1	1.331	0.000	2	0	-0.613	NF	0.649
151230	1	0.838	47.381	1	0	-0.604	NF	0.643
72064	2	2.758	67.566	0	0	-0.596	NF	0.640
92189	1	0.859	47.381	1	0	-0.587	NF	0.639
110992	1	1.808	48.625	0	0	-0.579	NF	0.637
1893	1	0.847	18.952	2	0	-0.522	NF	0.626
198707	1	2.583	27.778	0	0	-0.478	NF	0.615
121957	1	2.749	22.719	0	0	-0.471	NF	0.614
11425584	1	2.404	35.301	0	0	-0.431	NF	0.603
216249	1	2.344	39.149	0	0	-0.380	NF	0.591
2557	1	2.169	16.663	1	0	-0.298	NF	0.573
10007	1	1.869	58.101	0	0	-0.282	NF	0.565
13985	2	3.151	74.519	0	0	-0.089	NF	0.516
2078	1	2.498	46.079	0	0	-0.072	NF	0.514
73343	0	0.971	45.192	0	0	-0.065	NF	0.513
15402	0	0.550	60.363	0	0	-0.020	NF	0.500
88842	0	2.713	0.000	0	0	0.205	F	0.551
13643	1	0.920	47.381	2	0	0.278	F	0.573
2075	1	1.331	39.149	2	0	0.405	F	0.603
12130	0	0.804	17.650	2	0	0.706	F	0.671
1455	3	3.016	164.827	0	0	0.849	F	0.712
11316914	3	2.999	138.933	1	0	0.974	F	0.735
123805	1	3.500	56.788	0	0	1.037	F	0.742
13676	1	2.121	107.220	0	0	1.204	F	0.775
12620	0	2.886	56.981	0	0	1.831	F	0.864
6	1	1.331	39.149	4	0	2.031	F	0.885

CID: Puchem ID; D.F.: discriminant function; Cl.: classification; F: predicted as Fungicide by the model; NF: predicted as non-Fungicide by the model; C.L.: confidence level of the classification provided by the model; nR06: Topological descriptor (Count descriptor). Number of 6-membered rings in the molecule; SpMax8_Bh(m): The largest eigenvalue of a modified Burden matrix (B_h(m)) weighted by atomic masses (m); P_VSA_charge_6: The portion of the molecular Van der Waals surface area where the partial charge falls within a specific bin (bin 6); CATS2D_01_AA: A correlation vector descriptor that measures the occurrence of specific atom pairs (A-A) at a given topological distance (lag 01); B01[C-X]: Binary fingerprint. Presence of a carbon bonded to a heteroatom (N, O, S, etc.).

Table S2. Classification and probability of classification for training set compounds in Model 2 (LDA-AlvaDesc) trained with FRAC-WIPO data, focusing on Fungicide activity prediction.

CID	nStruct.	P_VSA_m_4	SsssN	NsCH3	CATS2D_04_DA	SHED_DL	DF	Cl.	C.L.
**Active Group**									
dodin	2	0.000	0.000	2	0	12.574	-4.344	NF	0.987
pencycuron	1	39.149	1.962	0	0	5.427	-2.030	NF	0.886
blasticidin_s	1	0.000	2.576	1	6	9.187	-1.582	NF	0.830
diclocymet	1	0.000	0.000	0	0	5.885	-1.539	NF	0.823
tridemorph	1	0.000	0.000	4	0	12.082	-1.370	NF	0.798
amobam	3	167.511	0.000	0	0	0.000	-1.314	NF	0.799
carbendazim	1	0.000	0.000	0	0	4.000	-1.011	NF	0.733
fenamidone	1	32.220	1.535	2	0	5.686	-0.993	NF	0.732
natamycin	1	0.000	0.000	0	0	3.780	-0.949	NF	0.721
vinclozoline	1	0.000	0.000	0	0	3.754	-0.942	NF	0.720
benodanil	1	0.000	0.000	0	0	3.649	-0.913	NF	0.714
fludioxonil	1	0.000	0.000	0	1	4.762	-0.901	NF	0.711
oxolinic_acid	1	0.000	1.710	1	2	3.789	-0.848	NF	0.700
prothiocarb	1	0.000	0.000	0	0	3.000	-0.731	NF	0.675
mandipropamid	1	39.149	0.000	1	0	6.529	-0.695	NF	0.671
ametoctradin	1	0.000	0.000	2	2	8.549	-0.687	NF	0.666
cyclobutrifluram	1	0.000	0.000	0	0	2.828	-0.683	NF	0.665
pyraziflumid	1	0.000	0.000	0	1	3.709	-0.607	NF	0.647
flutriafol	1	0.000	0.000	0	1	3.596	-0.575	NF	0.640
dodemorph	1	0.000	2.741	2	0	0.000	-0.570	NF	0.639
febram	2	91.299	1.713	2	0	0.000	-0.564	NF	0.646
ziram	2	91.299	1.713	2	0	0.000	-0.564	NF	0.646
cyprodinil_met	1	0.000	0.000	1	0	3.789	-0.476	NF	0.617
iodocarb	1	0.000	0.000	1	0	3.789	-0.476	NF	0.617
bitertanol	1	0.000	0.000	3	1	8.320	-0.470	NF	0.616
metominostrobin	1	0.000	0.000	2	1	6.598	-0.463	NF	0.614
benthiavalicarb	1	31.278	0.000	3	0	8.704	-0.462	NF	0.617
bitertanol	1	0.000	0.000	0	0	2.000	-0.451	NF	0.611
tavaborole	1	0.000	0.000	0	0	2.000	-0.451	NF	0.611
tolprocarb	1	0.000	0.000	3	0	6.982	-0.418	NF	0.603
prothioconazole	1	123.489	1.634	0	1	5.663	-0.397	NF	0.610
fluopyram	1	39.149	0.000	0	1	4.745	-0.349	NF	0.590
fluopyram	1	39.149	0.000	0	1	4.745	-0.349	NF	0.590
fenpyrazamine	1	32.220	2.693	3	2	4.883	-0.335	NF	0.586
mandestrobin	1	0.000	0.000	0	2	3.720	-0.287	NF	0.571
thiabendazole	1	31.278	0.000	0	0	2.872	-0.257	NF	0.567
propamocarb	1	0.000	2.082	3	1	3.000	-0.219	NF	0.555
mefentrifluconazole	1	39.149	0.000	1	1	5.858	-0.184	NF	0.550
mepanipyrim_met	1	0.000	0.000	2	0	4.353	-0.158	NF	0.540
prothiocarb	2	84.721	0.000	3	0	3.464	-0.131	NF	0.541
fenhexamid	1	78.297	0.000	1	0	6.406	-0.112	NF	0.536
sedaxane	1	0.000	0.000	1	2	4.690	-0.082	NF	0.521

methasulfocarb	1	39.149	2.469	4	1	5.329	-0.077	NF	0.523
octhilinone	1	31.981	1.833	1	0	0.000	-0.058	NF	0.518
pyraziflumid	1	0.000	0.000	0	2	2.828	-0.037	NF	0.509
tridemorph	1	0.000	2.590	3	0	0.000	-0.004	NF	0.501
aldimorph	1	0.000	2.587	3	0	0.000	-0.002	NF	0.500
flumorph	1	0.000	1.744	2	0	0.000	0.023	F	0.506
hymexazole	1	0.000	0.000	1	0	2.000	0.025	F	0.506
oxathiapiprolin	1	31.278	1.627	1	0	0.000	0.055	F	0.511
fenfuram	1	0.000	0.000	1	1	2.951	0.082	F	0.520
ipflufenoquin	1	0.000	0.000	3	1	6.336	0.086	F	0.521
proquinazid	1	0.000	1.633	2	0	0.000	0.089	F	0.522
benomyl	1	0.000	0.000	2	2	5.742	0.099	F	0.524
tolfenpyrad	1	39.149	0.000	3	1	8.220	0.106	F	0.522
biphenyl	1	0.000	0.000	0	0	0.000	0.109	F	0.527
cinnamaldehyde	1	0.000	0.000	0	0	0.000	0.109	F	0.527
metalaxyl	1	0.000	0.000	0	0	0.000	0.109	F	0.527
penthiopyrad	1	0.000	0.000	0	0	0.000	0.109	F	0.527
pyrazophos	1	0.000	0.000	0	0	0.000	0.109	F	0.527
triflumizole	1	0.000	0.000	0	0	0.000	0.109	F	0.527
mandestrobin	1	0.000	0.000	4	0	6.586	0.169	F	0.542
carbendazim	1	0.000	0.000	1	2	3.747	0.182	F	0.545
probenazole	1	5.807	0.000	0	0	0.000	0.190	F	0.547
fenpiclonil	1	78.297	0.000	0	1	4.757	0.196	F	0.541
cyclobutrifluram	1	78.297	0.000	0	1	4.690	0.215	F	0.545
phenamacril	1	0.000	0.000	1	3	4.757	0.222	F	0.555
phosphorous acid	1	9.204	0.000	0	0	0.000	0.238	F	0.558
dimethirimol	1	0.000	1.788	4	2	4.762	0.262	F	0.565
pyrimethanil_met	1	0.000	0.000	2	0	2.828	0.269	F	0.567
boscalid	1	78.297	0.000	0	1	4.436	0.286	F	0.563
fenpicoxamid	1	0.000	0.000	1	0	1.000	0.305	F	0.576
picarbutrazox	1	0.000	0.000	4	0	6.000	0.333	F	0.582
flutianil	1	64.439	1.862	1	0	0.000	0.379	F	0.588
fluxapyroxad	1	0.000	0.000	1	2	3.000	0.391	F	0.596
diclomezine	1	78.297	0.000	1	0	4.586	0.397	F	0.590
flusulfamide	1	84.105	0.000	0	0	3.155	0.403	F	0.591
fenpropidin	1	0.000	2.650	4	0	0.000	0.436	F	0.607
ferimzone	1	0.000	0.000	4	0	5.586	0.449	F	0.610
pyrisoxazole	1	39.149	1.934	2	0	0.000	0.458	F	0.609
fluquinconazole	1	78.297	1.245	0	0	0.000	0.464	F	0.607
cyproconazole	1	39.149	0.000	1	1	3.538	0.465	F	0.610
ethirimol	1	0.000	0.000	3	2	6.107	0.473	F	0.616
dimoxystrobin	1	0.000	0.000	4	1	6.586	0.492	F	0.620
metconazole	1	39.149	0.000	2	1	5.084	0.509	F	0.621
triticonazole	1	39.149	0.000	2	1	5.084	0.509	F	0.621
carboxin	1	32.220	0.000	1	1	2.942	0.535	F	0.628
dimethachlone	1	78.297	1.123	0	0	0.000	0.537	F	0.624
mepronil	1	0.000	0.000	3	1	4.594	0.574	F	0.639

tiadinil	1	0.000	0.000	0	4	2.828	0.609	F	0.647
terbinafine	1	0.000	2.303	4	0	0.000	0.643	F	0.655
epoxiconazole	1	39.149	0.000	0	0	0.000	0.657	F	0.655
fenarimol	1	78.297	0.000	0	2	4.216	0.671	F	0.654
propineb	2	182.598	0.000	1	0	2.000	0.697	F	0.651
validamycin	1	0.000	0.000	0	10	9.419	0.702	F	0.668
bupirimate	1	7.511	1.015	5	1	5.742	0.705	F	0.668
triforine	1	234.892	3.572	0	0	2.000	0.712	F	0.650
ofurace	1	39.149	1.493	2	0	0.000	0.721	F	0.669
tolfenpyrad	1	44.468	0.000	0	0	0.000	0.732	F	0.671
fluoroimide	1	78.297	0.796	0	0	0.000	0.732	F	0.668
natamycin	1	0.000	0.000	2	11	13.857	0.734	F	0.675
fluoroimide	1	0.000	0.000	0	2	0.000	0.755	F	0.680
furalaxyl	1	0.000	0.000	0	2	0.000	0.755	F	0.680
pyrimethanil_met	1	0.000	0.000	0	2	0.000	0.755	F	0.680
pyrimorph	1	0.000	0.000	0	2	0.000	0.755	F	0.680
zineb	2	182.598	0.000	0	0	0.000	0.781	F	0.670
oxycarboxin	1	6.046	0.000	1	3	3.000	0.799	F	0.689
methasulfocarb	1	39.969	0.000	2	0	2.872	0.816	F	0.690
isopyrazam	1	0.000	0.000	3	2	4.826	0.832	F	0.696
thifluzamide	1	31.278	0.000	1	1	1.569	0.907	F	0.710
tebuconazole	1	39.149	0.000	3	1	5.345	0.911	F	0.710
thiazole	1	77.385	0.000	0	0	1.000	0.912	F	0.707
polyoxin	1	0.000	0.607	0	7	3.789	0.948	F	0.720
pyraclostrobin	1	39.149	1.061	2	0	0.000	0.978	F	0.724
oxpoconazole	1	39.149	1.851	3	0	0.000	0.984	F	0.725
pyrimorph	1	39.149	1.815	3	0	0.000	1.005	F	0.729
spiroxamine	1	0.000	2.480	5	0	0.000	1.013	F	0.734
iprovalicarb	1	0.000	0.000	6	0	6.969	1.014	F	0.733
diethofencarb	1	0.000	0.000	4	1	4.711	1.017	F	0.734
tricyclazole	1	31.278	0.000	1	0	0.000	1.023	F	0.733
simeconazole	1	0.000	0.000	3	1	2.872	1.056	F	0.742
azoxystrobin	1	0.000	0.000	2	0	0.000	1.061	F	0.743
ferimzone	1	0.000	0.000	2	0	0.000	1.061	F	0.743
picoxystrobin	1	0.000	0.000	2	0	0.000	1.061	F	0.743
quinofumelin	1	0.000	0.000	2	0	0.000	1.061	F	0.743
benalaxyl-m	1	0.000	1.559	4	0	0.000	1.085	F	0.748
fluopicolide	1	117.446	0.000	0	0	2.337	1.099	F	0.741
triadimenol	1	39.149	0.000	3	1	4.586	1.124	F	0.752
fluoxastrobin	1	39.149	0.000	1	0	0.000	1.133	F	0.754
myclobutanil	1	39.149	0.000	1	0	0.000	1.133	F	0.754
pyridachlometyl	1	39.149	0.000	1	0	0.000	1.133	F	0.754
triflumizole	1	39.149	0.000	1	0	0.000	1.133	F	0.754
vinclozoline	1	78.297	0.888	1	0	0.000	1.153	F	0.754
furalaxyl	1	0.000	1.426	4	0	0.000	1.165	F	0.762
amisulbrom	1	11.376	0.861	3	0	0.000	1.184	F	0.765
isotianil	1	109.349	0.000	0	2	3.864	1.204	F	0.761

azaconazole	1	78.297	0.000	0	0	0.000	1.205	F	0.764
imazalil	1	78.297	0.000	0	0	0.000	1.205	F	0.764
quinoxyfen	1	78.297	0.000	0	0	0.000	1.205	F	0.764
prochloraz	1	117.446	1.680	1	0	0.000	1.230	F	0.765
tecloftalam	1	234.892	0.000	0	0	7.360	1.337	F	0.776
pyrametostrobin	1	0.000	1.087	4	0	0.000	1.366	F	0.797
procymidone	1	78.297	1.210	2	0	0.000	1.437	F	0.803
kresoxim-methyl	1	3.143	0.000	0	4	0.000	1.445	F	0.809
cymoxanil	1	0.000	0.000	2	3	2.000	1.470	F	0.813
acibenzolar-s-methyl	1	63.272	0.000	1	0	0.000	1.471	F	0.809
chinomethionat	1	64.439	0.000	1	0	0.000	1.487	F	0.812
edifenphos	1	65.623	0.000	1	0	0.000	1.504	F	0.814
fenazaquin	1	0.000	0.000	3	0	0.000	1.537	F	0.823
kresoxim-methyl	1	0.000	0.000	3	0	0.000	1.537	F	0.823
ofurace	1	0.000	0.000	3	0	0.000	1.537	F	0.823
orysastrobin	1	0.000	0.000	3	0	0.000	1.537	F	0.823
thiabendazole	1	0.000	0.000	3	0	0.000	1.537	F	0.823
trifloxystrobin	1	0.000	0.000	3	0	0.000	1.537	F	0.823
fluindapyr	1	0.000	0.000	4	2	3.947	1.554	F	0.825
cyazofamid	1	44.718	1.004	3	0	0.000	1.566	F	0.825
inpyrfluxam	1	0.000	0.000	4	2	3.888	1.570	F	0.828
diniconazole	1	78.297	0.000	3	1	4.924	1.577	F	0.824
captan	1	149.427	0.995	0	0	0.000	1.609	F	0.825
metalaxyl	1	0.000	1.444	5	0	0.000	1.630	F	0.836
metalaxyl_m	1	0.000	1.444	5	0	0.000	1.630	F	0.836
difenoconazole	1	78.297	0.000	1	0	0.000	1.681	F	0.839
etaconazole	1	78.297	0.000	1	0	0.000	1.681	F	0.839
penconazole	1	78.297	0.000	1	0	0.000	1.681	F	0.839
propiconazole	1	78.297	0.000	1	0	0.000	1.681	F	0.839
pyrifenox	1	78.297	0.000	1	0	0.000	1.681	F	0.839
tiadinil	1	70.201	0.000	2	2	3.586	1.686	F	0.840
dichlofluanid	1	115.847	1.666	2	0	0.000	1.691	F	0.838
orysastrobin	1	0.000	0.000	6	1	5.657	1.704	F	0.846
dichlobentiazox	1	115.157	0.000	0	0	0.000	1.721	F	0.842
penthiopyrad	1	31.278	0.000	4	2	4.860	1.736	F	0.848
chlozolinate	1	78.297	0.703	2	0	0.000	1.739	F	0.847
thiophanate	1	90.384	0.000	2	4	6.447	1.813	F	0.855
silthiofam	1	31.278	0.000	5	0	3.864	1.845	F	0.862
penflufen	1	0.000	0.000	5	2	4.594	1.849	F	0.864
thiram	1	150.748	3.788	4	0	0.000	1.869	F	0.859
oxytetracycline	1	0.000	1.293	3	11	8.579	1.919	F	0.872
zoxamide	1	117.446	0.000	3	0	4.477	1.928	F	0.868
valifenalate	1	39.149	0.000	5	2	6.242	1.935	F	0.872
fluazinam	1	78.297	0.000	0	4	2.000	1.937	F	0.870
fenpicoxamid	1	0.000	0.000	6	1	4.703	1.971	F	0.878
binapacryl	1	0.000	0.000	4	0	0.000	2.013	F	0.882
fluopicolide	1	0.000	0.000	4	0	0.000	2.013	F	0.882

isofetamid	1	31.278	0.000	6	0	4.826	2.052	F	0.885
pyraoxystrobin	1	39.149	0.000	3	0	0.000	2.085	F	0.888
triadimefon	1	39.149	0.000	3	0	0.000	2.085	F	0.888
pyributicarb	1	45.192	1.710	5	0	0.000	2.104	F	0.890
triclopyricarb	1	117.446	1.003	2	0	0.000	2.109	F	0.887
captafol	1	188.576	1.000	0	0	0.000	2.154	F	0.889
tolylfluanid	1	115.847	1.686	3	0	0.000	2.155	F	0.892
chloroneb	1	78.297	0.000	2	0	0.000	2.157	F	0.893
imibenconazole	1	149.665	0.000	0	0	0.000	2.204	F	0.895
carpropamid	1	117.446	0.000	3	0	3.454	2.214	F	0.897
fthalide	1	156.595	0.000	0	0	0.000	2.301	F	0.904
tecnazene	1	156.595	0.000	0	0	0.000	2.301	F	0.904
diclocymet	1	78.297	0.000	4	1	3.316	2.504	F	0.922
iprobenfos	1	41.093	0.000	4	0	0.000	2.588	F	0.929
pydiflumetofen	1	117.446	0.990	3	0	0.000	2.592	F	0.927
etridiazole	1	148.498	0.000	1	0	0.000	2.664	F	0.931
fosetyl-al	1	86.082	0.000	3	0	0.000	2.742	F	0.937
pyrazophos	1	52.819	0.000	4	0	0.000	2.752	F	0.939
quintozene	1	195.743	0.000	0	0	0.000	2.849	F	0.941
isoprothiolane	1	64.439	0.000	4	0	0.000	2.915	F	0.947
furametpyr	1	39.149	0.000	5	3	3.827	2.934	F	0.949
metrafenone	1	0.000	0.000	6	0	0.000	2.965	F	0.951
tebufloquin	1	0.000	0.000	6	0	0.000	2.965	F	0.951
laminarin	1	0.000	0.000	0	9	0.000	3.016	F	0.953
streptomycin	1	0.000	0.000	2	10	4.457	3.043	F	0.954
pyriofenone	1	39.149	0.000	6	0	0.000	3.513	F	0.971
tebuconazole	1	391.486	0.000	0	0	0.000	5.590	F	0.996
**Inactive Group**									
177358	5	105.003	0.000	1	0	0.000	-5.481	NF	0.996
117947705	1	39.149	1.907	0	2	15.839	-4.267	NF	0.986
11427553	1	0.000	1.900	0	0	11.452	-4.228	NF	0.986
1930	1	0.000	0.000	1	0	17.000	-4.175	NF	0.985
159947	4	0.000	0.000	3	0	0.000	-4.115	NF	0.984
91614	3	105.003	0.000	0	1	7.237	-3.892	NF	0.981
134819291	2	0.000	3.549	0	0	0.000	-3.887	NF	0.980
11291932	1	0.000	1.690	0	0	10.429	-3.817	NF	0.979
16046068	1	0.000	0.000	0	0	11.909	-3.225	NF	0.962
101673418	2	0.000	4.003	2	0	0.000	-3.205	NF	0.961
13320	2	52.501	0.000	2	0	10.961	-3.157	NF	0.960
10178705	1	39.149	3.574	0	2	8.094	-3.090	NF	0.957
93154	1	0.000	5.369	0	0	0.000	-3.086	NF	0.956
122536283	1	0.000	1.156	0	0	8.911	-3.074	NF	0.956
2448	1	0.000	2.297	0	1	7.585	-3.059	NF	0.955
161240	1	0.000	0.000	0	0	11.283	-3.050	NF	0.955
122737	1	0.000	4.135	2	1	6.727	-2.960	NF	0.951
119593	1	0.000	0.000	0	1	11.863	-2.890	NF	0.947
192706	1	0.000	4.721	3	0	5.744	-2.880	NF	0.947

101744	1	0.000	2.451	0	0	5.321	-2.839	NF	0.945
107896	1	0.000	1.516	1	0	8.742	-2.765	NF	0.941
185236	1	0.000	3.803	1	2	6.107	-2.742	NF	0.940
11689883	1	0.000	3.675	2	0	5.329	-2.618	NF	0.932
12447	2	44.468	0.000	1	0	6.803	-2.581	NF	0.931
100516	1	0.000	0.000	0	3	12.715	-2.482	NF	0.923
119198	1	0.000	4.735	3	0	3.864	-2.362	NF	0.914
11243969	1	0.000	1.804	0	0	4.848	-2.321	NF	0.911
2200	1	0.000	2.354	0	0	3.649	-2.313	NF	0.910
14987	1	0.000	2.380	1	1	6.427	-2.308	NF	0.910
158789	1	0.000	0.000	0	2	10.819	-2.274	NF	0.907
91268	2	0.000	2.369	2	0	0.000	-2.232	NF	0.903
16040217	5	4.992	0.000	0	16	0.000	-2.189	NF	0.900
13770	2	44.468	2.427	1	0	0.000	-2.121	NF	0.895
72076	1	0.000	0.000	0	0	7.855	-2.090	NF	0.890
11494412	1	0.000	0.000	0	0	7.669	-2.038	NF	0.885
151178	3	14.527	0.000	3	0	0.000	-2.028	NF	0.884
160154	1	0.000	2.597	1	0	3.769	-2.016	NF	0.882
10130337	1	0.000	0.000	0	0	7.445	-1.976	NF	0.878
11105	2	44.468	2.050	3	1	4.711	-1.940	NF	0.876
102669	1	0.000	2.314	2	0	5.744	-1.924	NF	0.873
221227	1	0.000	0.000	0	2	9.421	-1.883	NF	0.868
92411	1	39.149	2.795	2	1	7.279	-1.769	NF	0.856
164509	1	0.000	1.540	2	0	6.735	-1.741	NF	0.851
14454445	2	44.468	0.000	3	0	7.159	-1.729	NF	0.852
173250	1	0.000	3.683	3	0	3.789	-1.716	NF	0.848
134898	1	0.000	3.967	2	2	3.789	-1.714	NF	0.847
92965	2	44.468	2.431	2	0	0.000	-1.647	NF	0.841
11622909	1	0.000	1.919	2	0	5.586	-1.645	NF	0.838
83813	2	83.617	2.060	2	1	3.790	-1.616	NF	0.839
11316914	1	117.446	3.774	0	1	5.154	-1.613	NF	0.840
2169	1	5.807	0.000	2	0	9.644	-1.558	NF	0.827
2040	1	0.000	1.317	1	0	4.745	-1.527	NF	0.822
11623906	1	0.000	1.361	0	0	2.872	-1.505	NF	0.818
91452	2	44.468	0.000	2	0	4.586	-1.484	NF	0.818
14683796	1	0.000	2.394	3	0	5.586	-1.451	NF	0.810
2790	1	71.368	0.000	0	0	9.000	-1.412	NF	0.809
216239	1	39.149	0.000	1	0	8.837	-1.341	NF	0.796
159598	2	44.468	0.000	3	0	5.743	-1.333	NF	0.794
2315	1	11.615	0.000	0	2	7.941	-1.306	NF	0.788
2267	1	39.149	4.091	1	0	0.000	-1.301	NF	0.789
129791	2	44.468	2.068	4	0	2.889	-1.287	NF	0.787
15938	3	105.003	0.000	2	0	0.000	-1.237	NF	0.782
78569	1	0.000	0.000	0	0	4.757	-1.223	NF	0.773
10770	1	0.000	0.000	1	0	6.447	-1.220	NF	0.772
10718	1	0.000	0.000	0	3	8.200	-1.218	NF	0.772
132260161	1	62.555	1.107	0	1	6.614	-1.203	NF	0.774

166553	1	39.149	2.633	1	0	2.730	-1.198	NF	0.771
216468	1	50.764	0.000	0	0	7.098	-1.168	NF	0.767
92425	1	0.000	2.138	0	0	0.000	-1.163	NF	0.762
194066	1	0.000	0.000	2	0	7.855	-1.138	NF	0.758
15851	2	83.617	0.000	1	0	3.596	-1.135	NF	0.763
150315	1	0.000	0.000	0	0	4.409	-1.125	NF	0.755
153103	1	0.000	1.018	1	3	7.237	-1.078	NF	0.746
119574	1	39.149	2.484	1	3	5.970	-1.047	NF	0.744
159500	1	0.000	2.724	1	0	0.000	-1.036	NF	0.738
108089	1	0.000	2.445	2	1	3.364	-1.012	NF	0.734
216327	1	0.000	0.000	0	0	3.953	-0.998	NF	0.731
72413	1	0.000	1.057	0	2	4.000	-0.994	NF	0.730
12056759	2	5.807	1.210	3	0	0.000	-0.985	NF	0.729
11465618	1	39.149	5.157	3	0	0.000	-0.983	NF	0.731
126094	1	0.000	0.000	1	8	14.794	-0.973	NF	0.727
10275	1	31.278	0.000	0	0	5.329	-0.945	NF	0.723
152035	2	44.468	0.000	2	7	10.650	-0.922	NF	0.720
72103	1	0.000	0.000	1	3	8.818	-0.915	NF	0.715
2369	1	0.000	0.000	2	1	8.152	-0.899	NF	0.711
208947	1	0.000	0.000	2	0	6.928	-0.879	NF	0.707
15387	1	0.000	2.442	1	0	0.000	-0.868	NF	0.704
11960529	1	0.000	0.000	1	1	6.248	-0.841	NF	0.699
150949	1	39.149	0.000	0	0	5.201	-0.799	NF	0.693
12717441	1	0.000	2.320	1	0	0.000	-0.795	NF	0.689
1883	1	32.220	1.288	1	0	3.780	-0.789	NF	0.690
11326715	1	0.000	0.000	1	1	6.041	-0.784	NF	0.687
94532	1	0.000	2.137	3	0	3.747	-0.783	NF	0.687
15706387	1	0.000	0.000	1	0	4.745	-0.744	NF	0.678
1018	1	0.000	0.000	0	0	3.000	-0.731	NF	0.675
194680	1	78.297	0.000	1	0	8.606	-0.729	NF	0.682
10331863	1	0.000	0.000	2	0	6.336	-0.713	NF	0.671
11210478	1	0.000	0.000	1	1	5.686	-0.684	NF	0.665
172975	2	44.468	0.000	1	0	0.000	-0.676	NF	0.667
157839	1	31.278	0.000	0	0	4.327	-0.665	NF	0.663
100413	1	39.149	0.000	0	0	4.649	-0.645	NF	0.660
214356	1	3.143	0.000	0	0	2.828	-0.639	NF	0.655
98994	1	32.220	0.000	2	2	9.829	-0.594	NF	0.648
127151	1	0.000	0.000	1	1	5.326	-0.583	NF	0.642
73341	2	52.501	0.000	2	2	4.000	-0.562	NF	0.642
11984562	2	39.149	0.000	4	0	4.353	-0.542	NF	0.636
10313100	2	44.468	0.000	2	0	1.000	-0.480	NF	0.622
164867	1	44.956	0.000	0	0	4.353	-0.480	NF	0.622
10124	1	37.324	0.000	0	0	3.920	-0.466	NF	0.618
11683556	1	0.000	0.000	2	0	5.299	-0.423	NF	0.604
119114	1	31.278	0.000	0	1	4.586	-0.414	NF	0.605
10939	2	0.000	0.000	3	0	0.000	-0.347	NF	0.586
80084	2	0.000	0.000	3	0	0.000	-0.347	NF	0.586

13738	1	0.000	2.340	2	0	0.000	-0.331	NF	0.582
160883	1	0.000	0.000	2	2	7.237	-0.319	NF	0.580
2562	1	0.000	2.314	2	0	0.000	-0.316	NF	0.578
10250769	1	32.220	1.439	3	0	5.164	-0.314	NF	0.581
10013505	1	32.220	2.331	2	1	2.586	-0.276	NF	0.572
172309	1	0.000	3.040	3	0	0.000	-0.272	NF	0.567
134391533	1	0.000	0.000	4	0	8.151	-0.269	NF	0.567
93379	1	39.149	0.000	0	1	4.456	-0.268	NF	0.571
98895	1	0.000	0.000	1	0	2.971	-0.247	NF	0.562
13268	1	39.149	1.511	1	1	2.828	-0.235	NF	0.562
127873	2	2.496	0.000	1	8	5.526	-0.227	NF	0.557
196968	1	0.000	0.000	2	0	4.594	-0.225	NF	0.556
1533	1	0.000	0.000	1	0	2.828	-0.207	NF	0.552
460	1	0.000	0.000	1	0	2.828	-0.207	NF	0.552
163797	1	0.000	1.490	2	2	3.568	-0.179	NF	0.545
92266	1	32.220	0.000	0	1	3.789	-0.178	NF	0.548
192737	1	0.000	0.000	0	0	1.000	-0.171	NF	0.543
104901	1	0.000	2.058	2	0	0.000	-0.163	NF	0.541
15907	1	39.149	0.000	0	0	2.872	-0.147	NF	0.541
14343	1	0.000	0.000	1	2	4.757	-0.101	NF	0.526
159599	1	0.000	0.000	3	0	5.743	-0.071	NF	0.518
187888	1	0.000	1.065	1	0	0.000	-0.049	NF	0.512
122067	1	5.807	0.000	0	1	2.000	-0.047	NF	0.512
129228	1	0.000	0.000	0	2	2.828	-0.037	NF	0.509
126970	1	39.149	1.950	1	0	0.000	-0.027	NF	0.511
2712	1	39.149	1.170	1	2	3.947	-0.022	NF	0.510
227260	1	32.220	0.000	1	0	3.709	-0.002	NF	0.504
10445549	1	0.000	0.000	0	3	3.780	0.020	F	0.505
1858	1	39.149	0.000	2	0	5.640	0.030	F	0.503
11236633	1	78.297	0.000	0	0	4.000	0.085	F	0.513
14129	1	0.000	0.000	0	0	0.000	0.109	F	0.527
123920	1	78.297	0.000	0	0	3.864	0.123	F	0.523
121940	1	32.220	1.504	2	0	1.755	0.126	F	0.528
182137	1	39.149	0.000	1	0	3.596	0.126	F	0.527
187238	1	0.000	0.000	3	2	7.242	0.155	F	0.538
2583	1	0.000	0.000	3	2	7.129	0.187	F	0.546
10358610	1	78.297	0.000	1	1	6.334	0.231	F	0.549
10245201	1	0.000	2.158	3	0	0.000	0.253	F	0.563
10090	1	0.000	2.142	3	0	0.000	0.262	F	0.565
2155	1	31.278	0.000	0	0	1.000	0.267	F	0.563
211207	1	12.720	0.000	0	0	0.000	0.287	F	0.570
219069	1	39.149	0.609	0	0	0.000	0.295	F	0.569
91683	1	39.149	0.000	0	2	3.543	0.311	F	0.573
10848	1	78.297	2.222	1	0	0.000	0.359	F	0.581
192251	2	52.501	0.000	3	0	0.000	0.388	F	0.591
160557	1	60.363	0.000	0	0	2.000	0.394	F	0.591
13726	1	78.297	0.000	0	0	2.828	0.413	F	0.594

91741	1	78.297	0.000	0	1	3.930	0.428	F	0.598
216249	1	44.956	0.000	2	1	5.451	0.487	F	0.615
2293	1	0.000	2.521	4	0	0.000	0.513	F	0.626
91739	1	78.297	0.000	0	1	3.586	0.524	F	0.621
2081	1	39.149	0.000	3	0	5.451	0.559	F	0.632
15459	1	0.000	0.000	2	1	2.942	0.560	F	0.636
219095	1	39.149	0.000	3	1	6.586	0.564	F	0.633
12302171	1	0.000	0.000	0	10	9.873	0.575	F	0.639
108137	1	0.000	0.000	1	0	0.000	0.585	F	0.642
2683	1	0.000	0.000	1	0	0.000	0.585	F	0.642
82143	1	0.000	0.000	1	0	0.000	0.585	F	0.642
6	1	39.149	0.000	0	0	0.000	0.657	F	0.655
115239	1	6.046	0.000	1	0	0.000	0.670	F	0.661
72154	1	110.517	0.000	1	0	4.711	0.813	F	0.683
194233	1	38.564	0.000	1	0	1.000	0.845	F	0.696
10199199	1	39.149	0.000	3	0	4.350	0.867	F	0.701
10262683	1	60.363	0.000	1	0	2.000	0.870	F	0.700
127657	1	136.579	0.000	1	0	5.657	0.913	F	0.702
92357	1	38.810	1.076	2	0	0.000	0.964	F	0.721
11339	1	1.339	0.000	4	2	6.089	0.973	F	0.725
15965	1	117.446	0.000	0	1	3.780	1.018	F	0.725
76962	1	0.000	0.000	3	0	1.755	1.046	F	0.740
100580	1	0.000	0.000	2	0	0.000	1.061	F	0.743
15939	1	0.000	0.000	2	0	0.000	1.061	F	0.743
2048	1	0.000	0.000	2	0	0.000	1.061	F	0.743
2391	1	0.000	0.000	2	0	0.000	1.061	F	0.743
10547	2	41.093	0.000	5	0	0.000	1.180	F	0.762
11405965	1	39.149	1.328	3	0	0.000	1.295	F	0.782
219104	1	60.363	0.000	2	0	1.890	1.377	F	0.795
10419733	1	64.325	0.000	1	0	0.000	1.486	F	0.812
1684	1	0.000	0.000	3	0	0.000	1.537	F	0.823
10783	1	70.426	0.000	1	0	0.000	1.571	F	0.824
2451	1	70.426	0.000	1	0	0.000	1.571	F	0.824
130305	1	0.000	0.000	6	2	7.173	1.602	F	0.832
123619	1	45.195	0.000	2	0	0.000	1.694	F	0.842
121596244	1	0.000	0.000	3	8	8.268	1.806	F	0.858
992	1	195.743	0.000	0	0	2.872	2.045	F	0.877

CID: compound ID; nStruct.: number of structures; D.F.: discriminant function; Cl.: Classification; F: predicted as Fungicide by the model; NF: predicted as non-Fungicide by the model; C.L.: confidence level of the classification provided by the model; P_VSA_m_4: Mass-weighted VSA. VdW surface area where atomic mass falls in bin 4; SsssN: E-State descriptor. E-State sum for quaternary nitrogens (N with 4 single bonds); NsCH3: Atom-centered fragment. Count of methyl groups ($-CH_3$) attached to sulfur (S); CATS2D_04_DA: CATS2D correlation vector. Donor-acceptor atom pairs separated by 4 bonds; SHED_DL: Shannon entropy descriptor. Entropy of drug-like atomic property distributions.

Table S3. Classification and probability of classification for training set compounds in Model 3 (LDA-AlvaDesc) trained with Pubchem-WIPO data, focusing on Fungicide activity prediction.

CID	MATS2p	SaasC	SsCl	NaaN	DF	Cl.	C.L.
**Active Group**							
6327657	0.111	4.630	0.000	0.000	-2.260	NF	0.905
11159	-0.233	0.000	0.000	0.000	-1.955	NF	0.876
16682942	-0.219	0.000	0.000	0.000	-1.884	NF	0.868
32518	-0.161	0.000	0.000	0.000	-1.596	NF	0.832
3032792	-0.064	1.097	0.000	0.000	-1.592	NF	0.831
11292824	0.050	3.388	5.913	0.000	-1.308	NF	0.787
50367	0.017	0.926	0.000	0.000	-1.109	NF	0.752
18771	0.061	0.698	0.000	0.000	-0.787	NF	0.687
3032581	0.103	0.000	0.000	0.000	-0.275	NF	0.568
5430	-0.290	1.749	0.000	2.000	-0.214	NF	0.553
9578570	0.223	0.500	0.000	0.000	0.106	F	0.526
30154	0.186	0.000	0.000	0.000	0.141	F	0.535
10788	0.310	0.000	0.000	0.000	0.763	F	0.682
39676	0.124	0.920	11.668	0.000	0.840	F	0.698
7430	0.092	0.173	11.099	0.000	0.936	F	0.718
25429	0.142	0.387	0.000	1.000	1.149	F	0.759
24462	0.390	0.000	0.000	0.000	1.165	F	0.762
5455	0.509	0.000	0.000	0.000	1.757	F	0.853
1810180	-0.019	0.000	0.000	2.000	1.912	F	0.871
15910	-0.018	-0.273	22.812	0.000	2.003	F	0.880
86132	-0.002	1.563	5.950	2.000	2.029	F	0.884
3034285	0.175	1.928	0.000	2.000	2.038	F	0.885
17581	0.495	2.058	11.633	0.000	2.192	F	0.899
8607	0.266	0.656	16.603	0.000	2.262	F	0.905
86173	0.042	3.120	12.475	2.000	2.357	F	0.913
9257	0.076	0.000	0.000	2.000	2.386	F	0.916
91699	0.131	-1.105	5.673	1.000	2.435	F	0.919
41368	0.127	1.240	5.850	2.000	2.806	F	0.943
39385	0.153	1.157	5.833	2.000	2.970	F	0.951
66461	0.073	1.695	12.164	2.000	3.098	F	0.957
2730	0.154	0.551	17.487	1.000	3.256	F	0.963
6720	0.194	-1.669	27.934	0.000	4.291	F	0.986
17432	0.271	0.569	16.587	2.000	5.121	F	0.994
**Inactive Group**							
3036461	-0.020	5.935	0.000	0.000	-3.490	NF	0.970
6371	-0.666	0.000	8.802	0.000	-3.055	NF	0.955
36324	0.266	6.823	0.000	0.000	-2.446	NF	0.920
65856	-0.088	2.615	0.000	0.000	-2.375	NF	0.915
36920	0.040	3.554	0.000	0.000	-2.143	NF	0.895
66368	-0.101	1.914	0.000	0.000	-2.131	NF	0.894
6917740	0.005	3.080	0.000	0.000	-2.113	NF	0.892
5359271	0.126	4.427	0.000	0.000	-2.097	NF	0.890
657310	0.076	5.158	6.198	0.000	-1.919	NF	0.872

9844338	-0.007	2.344	0.000	0.000	-1.851	NF	0.864
7568320	-0.201	0.000	0.000	0.000	-1.796	NF	0.858
66384	-0.073	1.173	0.000	0.000	-1.668	NF	0.841
5387	0.159	3.663	0.000	0.000	-1.599	NF	0.832
9548842	-0.146	0.000	0.000	0.000	-1.520	NF	0.821
15365	-0.140	0.000	0.000	0.000	-1.487	NF	0.816
177335	-0.009	1.458	0.000	0.000	-1.473	NF	0.813
3058751	-0.058	0.651	0.000	0.000	-1.366	NF	0.797
23663941	0.009	1.409	0.000	0.000	-1.360	NF	0.796
16351	0.218	5.358	6.278	0.000	-1.285	NF	0.783
21720	0.049	1.657	0.000	0.000	-1.268	NF	0.780
2955	0.044	1.496	0.000	0.000	-1.226	NF	0.773
10461	-0.336	0.000	10.747	0.000	-1.169	NF	0.764
6917865	-0.004	0.809	0.000	0.000	-1.163	NF	0.762
68889	-0.048	1.605	5.693	0.000	-1.042	NF	0.740
61948	-0.050	0.000	0.000	0.000	-1.037	NF	0.738
5483	-0.049	0.000	0.000	0.000	-1.037	NF	0.738
7083	0.313	4.054	0.000	0.000	-1.000	NF	0.731
64393	-0.040	0.000	0.000	0.000	-0.988	NF	0.729
636970	-0.121	2.262	0.000	1.000	-0.987	NF	0.728
9838802	0.062	1.087	0.000	0.000	-0.957	NF	0.722
171450	-0.022	0.000	0.000	0.000	-0.898	NF	0.711
42510	0.208	2.548	0.000	0.000	-0.865	NF	0.704
65701	-0.079	2.412	0.000	1.000	-0.845	NF	0.699
9813116	-0.008	0.000	0.000	0.000	-0.830	NF	0.696
82238	0.000	0.000	0.000	0.000	-0.789	NF	0.688
5479	-0.102	0.266	0.000	1.000	-0.017	NF	0.504
216236	0.155	1.471	5.990	0.000	0.067	F	0.516
9571001	0.185	0.000	0.000	0.000	0.137	F	0.534
120202	0.077	1.909	0.000	1.000	0.159	F	0.540
7215	0.199	0.000	0.000	0.000	0.208	F	0.552
16367	-0.007	0.404	0.000	1.000	0.396	F	0.598
3034005	0.056	1.090	0.000	1.000	0.411	F	0.601
8590	-0.031	2.792	0.000	2.000	0.625	F	0.651
44158	-0.026	2.940	23.743	0.000	0.668	F	0.659
33557	0.333	0.000	0.000	0.000	0.879	F	0.707
25147683	-0.046	-4.659	0.000	0.000	1.023	F	0.735

CID: Pubchem ID; D.F.: discriminant function; Cl.: classification; F: predicted as Fungicide by the model; NF: predicted as non-Fungicide by the model; C.L.: confidence level of the classification provided by the model; MATS2p: Moran Autocorrelation (2D) descriptor (weighted by atomic polarizabilities); SaasC: E-State Atom-Type Descriptor (Electrotopological State). Sum of E-State values for carbon atoms (C) in the topological environment "aas"; SsCl: E-State Atom-Type Descriptor. Sum of E-State values for chlorine atoms (Cl) in the topological environment "s"; NaaN: Atom-Centered Fragment (ACF) Descriptor. Count of nitrogen atoms (N) in the topological environment "aaN".

Table S4. Classification and probability of classification for training set compounds in Model 4 (ANN-AlvaDesc) trained with WIPO data, focusing on Fungicide activity prediction.

CID	nR06	SpMax8_Bh(m)	P_VSA_charge_6	CATS2D_01_AA	B01[C-X]	Cl.	C.L.
**Active Group**							
50367	2.000	2.518	60.648	0.000	0.000	F	0.505
10935908	2.000	2.689	56.857	0.000	0.000	F	0.508
91699	1.000	2.501	47.974	0.000	0.000	F	0.512
17110	0.000	2.438	31.636	0.000	0.000	F	0.514
3037	2.000	2.431	78.297	0.000	0.000	F	0.519
9492	1.000	1.331	0.000	4.000	0.000	F	0.523
18771	3.000	2.510	102.344	0.000	0.000	F	0.526
6191	1.000	1.331	9.476	4.000	0.000	F	0.535
7744	0.000	2.730	44.309	0.000	0.000	F	0.541
135083	2.000	2.562	0.000	4.000	0.000	F	0.543
6437379	1.000	2.530	75.810	0.000	0.000	F	0.544
6451142	1.000	2.772	67.577	0.000	0.000	F	0.545
7223	0.000	2.932	44.309	0.000	0.000	F	0.550
11159	0.000	2.933	44.309	0.000	0.000	F	0.550
86173	2.000	2.687	78.297	1.000	0.000	F	0.555
39676	1.000	2.323	94.064	0.000	0.000	F	0.555
22321033	1.000	1.331	0.000	5.000	0.000	F	0.555
86132	1.000	2.400	66.926	1.000	0.000	F	0.556
7430	1.000	1.331	78.297	2.000	0.000	F	0.557
41368	1.000	2.499	65.624	1.000	0.000	F	0.558
5455	0.000	1.939	90.384	0.000	0.000	F	0.562
11048796	4.000	2.916	96.453	2.000	0.000	F	0.562
122087	1.000	2.714	87.122	0.000	0.000	F	0.564
11292824	2.000	2.989	101.639	0.000	0.000	F	0.568
6950	1.000	1.979	17.650	4.000	0.000	F	0.570
2730	1.000	2.333	117.446	0.000	0.000	F	0.583
17776	1.000	2.446	17.650	4.000	0.000	F	0.587
17432	0.000	1.807	117.446	0.000	0.000	F	0.590
14994	1.000	2.444	26.476	4.000	0.000	F	0.598
15910	1.000	1.708	156.595	0.000	0.000	F	0.605
92200	2.000	2.581	154.823	0.000	0.000	F	0.610
8607	1.000	2.491	136.398	0.000	0.000	F	0.612
59649244	2.000	2.842	124.882	1.000	0.000	F	0.613
11664966	2.000	2.838	105.133	2.000	0.000	F	0.618
213016	2.000	2.838	105.133	2.000	0.000	F	0.618
124962	1.000	3.589	20.185	4.000	0.000	F	0.631
45380430	2.000	3.058	173.954	2.000	0.000	F	0.705
6720	1.000	1.603	195.743	2.000	0.000	F	0.717
11486133	1.000	2.911	115.504	4.000	0.000	F	0.722
72980153	1.000	2.911	115.504	4.000	0.000	F	0.722
6327657	3.000	2.554	0.000	0.000	1.000	F	0.758
7511	1.000	0.341	0.000	0.000	1.000	F	0.763
5460680	1.000	0.496	0.000	0.000	1.000	F	0.766

25202562	1.000	1.039	0.000	0.000	1.000	F	0.779
7627	1.000	1.039	0.000	0.000	1.000	F	0.779
14309	1.000	1.039	0.000	0.000	1.000	F	0.779
65258	1.000	1.039	0.000	0.000	1.000	F	0.779
3647458	1.000	1.039	0.000	0.000	1.000	F	0.779
16682983	1.000	1.286	0.000	0.000	1.000	F	0.784
10598	2.000	2.687	0.000	0.000	1.000	F	0.786
16682942	0.000	0.083	0.000	0.000	1.000	F	0.787
56840815	0.000	0.083	0.000	0.000	1.000	F	0.787
12318	0.000	0.083	0.000	0.000	1.000	F	0.787
16682936	0.000	0.820	0.000	0.000	1.000	F	0.803
3032792	1.000	2.819	18.952	0.000	0.000	NF	0.504
17581	1.000	1.351	78.297	0.000	0.000	NF	0.505
33112	0.000	2.361	8.825	0.000	0.000	NF	0.514
5634	0.000	1.787	19.614	0.000	0.000	NF	0.528
10788	1.000	0.608	77.412	0.000	0.000	NF	0.540
5430	1.000	1.802	18.952	0.000	0.000	NF	0.550
3032581	0.000	1.651	0.000	0.000	0.000	NF	0.556
23663539	1.000	1.843	0.000	0.000	0.000	NF	0.568
54682462	1.000	1.331	0.000	0.000	0.000	NF	0.591
23690429	1.000	0.882	0.000	0.000	0.000	NF	0.612
9257	0.000	0.000	0.000	0.000	0.000	NF	0.631
30154	0.000	0.000	0.000	0.000	0.000	NF	0.631
5366415	0.000	0.000	0.000	0.000	0.000	NF	0.631
**Inactive Group**							
13643	1.000	0.920	47.381	2.000	0.000	F	0.501
2075	1.000	1.331	39.149	2.000	0.000	F	0.508
2078	1.000	2.498	46.079	0.000	0.000	F	0.510
92411	2.000	2.838	56.799	0.000	0.000	F	0.515
11001318	2.000	2.337	78.297	0.000	0.000	F	0.515
2789	2.000	2.613	67.577	0.000	0.000	F	0.516
170317	3.000	2.852	77.054	0.000	0.000	F	0.516
222284	3.000	3.049	68.148	0.000	0.000	F	0.516
72064	2.000	2.758	67.566	0.000	0.000	F	0.522
12620	0.000	2.886	56.981	0.000	0.000	F	0.562
13676	1.000	2.121	107.220	0.000	0.000	F	0.562
123805	1.000	3.500	56.788	0.000	0.000	F	0.564
6	1.000	1.331	39.149	4.000	0.000	F	0.573
11316914	3.000	2.999	138.933	1.000	0.000	F	0.609
1455	3.000	3.016	164.827	0.000	0.000	F	0.613
10350985	6.000	3.718	0.000	0.000	1.000	F	0.717
127151	3.000	2.695	66.334	0.000	0.000	NF	0.501
121957	1.000	2.749	22.719	0.000	0.000	NF	0.503
93379	2.000	2.173	67.577	0.000	0.000	NF	0.503
168884	3.000	2.866	54.985	0.000	0.000	NF	0.504
216249	1.000	2.344	39.149	0.000	0.000	NF	0.504
10007	1.000	1.869	58.101	0.000	0.000	NF	0.505

198707	1.000	2.583	27.778	0.000	0.000	NF	0.506
11425584	1.000	2.404	35.301	0.000	0.000	NF	0.506
88842	0.000	2.713	0.000	0.000	0.000	NF	0.508
219095	3.000	2.890	48.625	0.000	0.000	NF	0.509
2557	1.000	2.169	16.663	1.000	0.000	NF	0.510
12130	0.000	0.804	17.650	2.000	0.000	NF	0.512
15706387	2.000	2.308	49.916	0.000	0.000	NF	0.516
216239	3.000	2.861	39.149	0.000	0.000	NF	0.519
72300	2.000	2.432	37.905	0.000	0.000	NF	0.522
154257	3.000	3.057	24.022	0.000	0.000	NF	0.523
119569	1.000	2.503	8.825	0.000	0.000	NF	0.528
100516	3.000	2.969	18.952	0.000	0.000	NF	0.532
82178	3.000	2.517	41.091	0.000	0.000	NF	0.533
151230	1.000	0.838	47.381	1.000	0.000	NF	0.534
73343	0.000	0.971	45.192	0.000	0.000	NF	0.536
133128	2.000	2.898	0.000	0.000	0.000	NF	0.536
15402	0.000	0.550	60.363	0.000	0.000	NF	0.536
1893	1.000	0.847	18.952	2.000	0.000	NF	0.537
15532	1.000	1.331	0.000	2.000	0.000	NF	0.538
1864	1.000	2.266	0.000	0.000	0.000	NF	0.548
130229	2.000	2.629	0.000	0.000	0.000	NF	0.549
168045	1.000	2.164	0.000	0.000	0.000	NF	0.553
101815861	0.000	1.585	0.000	0.000	0.000	NF	0.559
73801	3.000	2.660	0.000	0.000	0.000	NF	0.562
999	1.000	0.839	47.381	0.000	0.000	NF	0.563
159247	0.000	1.224	9.476	0.000	0.000	NF	0.565
112056	1.000	1.368	18.952	0.000	0.000	NF	0.570
78782	1.000	0.841	39.149	0.000	0.000	NF	0.572
10419733	0.000	1.234	0.000	0.000	0.000	NF	0.575
11033	1.000	1.667	0.000	0.000	0.000	NF	0.576
101209015	1.000	1.283	14.243	0.000	0.000	NF	0.579
1720	3.000	1.789	18.952	0.000	0.000	NF	0.586
1050	1.000	1.418	0.000	0.000	0.000	NF	0.587
76962	0.000	0.083	8.825	1.000	0.000	NF	0.587
77139	1.000	1.331	0.000	0.000	0.000	NF	0.591
227	1.000	0.838	18.952	0.000	0.000	NF	0.594
1140	1.000	0.083	50.567	0.000	0.000	NF	0.594
89124	0.000	0.083	15.767	0.000	0.000	NF	0.610
114681	1.000	0.710	8.825	0.000	0.000	NF	0.611
10783	0.000	0.348	0.000	0.000	0.000	NF	0.616
457	1.000	0.620	0.000	0.000	0.000	NF	0.624
10903489	0.000	0.083	0.000	0.000	0.000	NF	0.628
10313100	0.000	0.083	0.000	0.000	0.000	NF	0.628
1646	0.000	0.083	0.000	0.000	0.000	NF	0.628
194233	0.000	0.083	0.000	0.000	0.000	NF	0.628
232487	1.000	0.445	0.000	0.000	0.000	NF	0.632
1727	1.000	0.083	0.000	0.000	0.000	NF	0.648

CID: Pubchem ID; Cl.: classification; F: predicted as Fungicide by the model; NF: predicted as non-Fungicide by the model; C.L.: confidence level of the classification provided by the model; nR06: Topological descriptor (Count descriptor). Number of 6-membered rings in the molecule; SpMax8_Bh(m): The largest eigenvalue of a modified Burden matrix (B_h(m)) weighted by atomic masses (m); P_VSA_charge_6: The portion of the molecular Van der Waals surface area where the partial charge falls within a specific bin (bin 6). CATS2D_01_AA: A correlation vector descriptor that measures the occurrence of specific atom pairs (A-A) at a given topological distance (lag 01). B01[C-X]: Binary fingerprint. Presence of a carbon bonded to a heteroatom (N, O, S, etc.).

Table S5. Classification and probability of classification for training set compounds in Model 5 (ANN-AlvaDesc) trained with FRAC-WIPO data, focusing on Fungicide activity prediction.

CID	nStruct.	P_VSA_m_4	SsssN	NsCH3	CATS2D_04_DA	SHED_DL	Class.	C.L.
**Active Group**								
myclobutanil	1	39.149	0.000	1	0	0.000	F	0.502
triflumizole	1	39.149	0.000	1	0	0.000	F	0.502
natamycin	1	0.000	0.000	0	0	3.780	F	0.503
triticonazole	1	39.149	0.000	2	1	5.084	F	0.504
vinclozoline	1	0.000	0.000	0	0	3.754	F	0.505
ethirimol	1	0.000	0.000	3	2	6.107	F	0.510
tolfenpyrad	1	44.468	0.000	0	0	0.000	F	0.521
validamycin	1	0.000	0.000	0	10	9.419	F	0.521
ferimzone	1	0.000	0.000	2	0	0.000	F	0.526
azoxystrobin	1	0.000	0.000	2	0	0.000	F	0.526
picoxystrobin	1	0.000	0.000	2	0	0.000	F	0.526
phosphorous acid	1	9.204	0.000	0	0	0.000	F	0.530
prothiocarb	1	0.000	0.000	0	0	3.000	F	0.534
isopyrazam	1	0.000	0.000	3	2	4.826	F	0.539
probenazole	1	5.807	0.000	0	0	0.000	F	0.540
mandestrobin	1	0.000	0.000	0	2	3.720	F	0.543
oxathiapiprolin	1	31.278	1.627	1	0	0.000	F	0.555
propamocarb	1	0.000	2.082	3	1	3.000	F	0.555
pyraziflumid	1	0.000	0.000	0	1	3.709	F	0.556
prothioconazole	1	123.489	1.634	0	1	5.663	F	0.557
metalaxyl	1	0.000	0.000	0	0	0.000	F	0.559
penthiopyrad	1	0.000	0.000	0	0	0.000	F	0.559
triflumizole	1	0.000	0.000	0	0	0.000	F	0.559
biphenyl	1	0.000	0.000	0	0	0.000	F	0.559
cinnamaldehyde	1	0.000	0.000	0	0	0.000	F	0.559
thiazole	1	77.385	0.000	0	0	1.000	F	0.560
fenpicoxamid	1	0.000	0.000	1	0	1.000	F	0.562
flutriafol	1	0.000	0.000	0	1	3.596	F	0.564
sedaxane	1	0.000	0.000	1	2	4.690	F	0.566
fenarimol	1	78.297	0.000	0	2	4.216	F	0.568
iodocarb	1	0.000	0.000	1	0	3.789	F	0.569
cyproconazole	1	39.149	0.000	1	1	3.538	F	0.577
tolprocarb	1	0.000	0.000	3	0	6.982	F	0.578
hymexazole	1	0.000	0.000	1	0	2.000	F	0.582
pyrisoxazole	1	39.149	1.934	2	0	0.000	F	0.589
tiadinil	1	0.000	0.000	0	4	2.828	F	0.589
ipflufenoquin	1	0.000	0.000	3	1	6.336	F	0.597
fenpropidin	1	0.000	2.650	4	0	0.000	F	0.598
pyraziflumid	1	0.000	0.000	0	2	2.828	F	0.606
pyraclostrobin	1	39.149	1.061	2	0	0.000	F	0.606
acibenzolar-s-methy	1	63.272	0.000	1	0	0.000	F	0.606
carboxin	1	32.220	0.000	1	1	2.942	F	0.608

benthiavalicarb	1	31.278	0.000	3	0	8.704	F	0.611
terbinafine	1	0.000	2.303	4	0	0.000	F	0.615
edifenphos	1	65.623	0.000	1	0	0.000	F	0.618
fluopicolide	1	117.446	0.000	0	0	2.337	F	0.624
bupirimate	1	7.511	1.015	5	1	5.742	F	0.628
oxpoconazole	1	39.149	1.851	3	0	0.000	F	0.632
fosetyl-al	1	86.082	0.000	3	0	0.000	F	0.633
amisulbrom	1	11.376	0.861	3	0	0.000	F	0.634
chloroneb	1	78.297	0.000	2	0	0.000	F	0.634
fenpyrazamine	1	32.220	2.693	3	2	4.883	F	0.636
pyrimorph	1	39.149	1.815	3	0	0.000	F	0.638
carbendazim	1	0.000	0.000	1	2	3.747	F	0.639
ofurace	1	39.149	1.493	2	0	0.000	F	0.641
iprovalicarb	1	0.000	0.000	6	0	6.969	F	0.648
bitertanol	1	0.000	0.000	3	1	8.320	F	0.651
azaconazole	1	78.297	0.000	0	0	0.000	F	0.658
imazalil	1	78.297	0.000	0	0	0.000	F	0.658
oxycarboxin	1	6.046	0.000	1	3	3.000	F	0.659
natamycin	1	0.000	0.000	2	11	13.857	F	0.662
fenfuram	1	0.000	0.000	1	1	2.951	F	0.665
tecloftalam	1	234.892	0.000	0	0	7.360	F	0.666
isotianil	1	109.349	0.000	0	2	3.864	F	0.668
fluxapyroxad	1	0.000	0.000	1	2	3.000	F	0.669
difenoconazole	1	78.297	0.000	1	0	0.000	F	0.669
etaconazole	1	78.297	0.000	1	0	0.000	F	0.669
penconazole	1	78.297	0.000	1	0	0.000	F	0.669
propiconazole	1	78.297	0.000	1	0	0.000	F	0.669
pyrifenox	1	78.297	0.000	1	0	0.000	F	0.669
simeconazole	1	0.000	0.000	3	1	2.872	F	0.673
orysastrobin	1	0.000	0.000	6	1	5.657	F	0.674
fluindapyr	1	0.000	0.000	4	2	3.947	F	0.678
tolfenpyrad	1	39.149	0.000	3	1	8.220	F	0.679
blasticidin_s	1	0.000	2.576	1	6	9.187	F	0.680
triadimenol	1	39.149	0.000	3	1	4.586	F	0.681
fluoroimide	1	78.297	0.796	0	0	0.000	F	0.681
inpyrfluxam	1	0.000	0.000	4	2	3.888	F	0.683
ofurace	1	0.000	0.000	3	0	0.000	F	0.685
orysastrobin	1	0.000	0.000	3	0	0.000	F	0.685
thiabendazole	1	0.000	0.000	3	0	0.000	F	0.685
trifloxystrobin	1	0.000	0.000	3	0	0.000	F	0.685
diniconazole	1	78.297	0.000	3	1	4.924	F	0.690
flutianil	1	64.439	1.862	1	0	0.000	F	0.694
zoxamide	1	117.446	0.000	3	0	4.477	F	0.698
chlozolinate	1	78.297	0.703	2	0	0.000	F	0.699
dimoxystrobin	1	0.000	0.000	4	1	6.586	F	0.701
dimethachlone	1	78.297	1.123	0	0	0.000	F	0.702
diethofencarb	1	0.000	0.000	4	1	4.711	F	0.703

fluoroimide	1	0.000	0.000	0	2	0.000	F	0.704
furalaxyl	1	0.000	0.000	0	2	0.000	F	0.704
pyrimethanil_met	1	0.000	0.000	0	2	0.000	F	0.704
pyrimorph	1	0.000	0.000	0	2	0.000	F	0.704
febram	2	91.299	1.713	2	0	0.000	F	0.706
ziram	2	91.299	1.713	2	0	0.000	F	0.706
vinclozoline	1	78.297	0.888	1	0	0.000	F	0.706
benalaxyl-m	1	0.000	1.559	4	0	0.000	F	0.706
fluquinconazole	1	78.297	1.245	0	0	0.000	F	0.707
oxytetracycline	1	0.000	1.293	3	11	8.579	F	0.710
tiadinil	1	70.201	0.000	2	2	3.586	F	0.710
ferimzone	1	0.000	0.000	4	0	5.586	F	0.715
methasulfocarb	1	39.149	2.469	4	1	5.329	F	0.716
picarbutrazox	1	0.000	0.000	4	0	6.000	F	0.717
carpropamid	1	117.446	0.000	3	0	3.454	F	0.718
valifenalate	1	39.149	0.000	5	2	6.242	F	0.719
procymidone	1	78.297	1.210	2	0	0.000	F	0.719
kresoxim-methyl	1	3.143	0.000	0	4	0.000	F	0.720
isoprothiolane	1	64.439	0.000	4	0	0.000	F	0.720
laminarin	1	0.000	0.000	0	9	0.000	F	0.721
fenpicoxamid	1	0.000	0.000	6	1	4.703	F	0.721
spiroxamine	1	0.000	2.480	5	0	0.000	F	0.724
dichlobentiazox	1	115.157	0.000	0	0	0.000	F	0.725
pyrametostrobin	1	0.000	1.087	4	0	0.000	F	0.726
penthiopyrad	1	31.278	0.000	4	2	4.860	F	0.727
streptomycin	1	0.000	0.000	2	10	4.457	F	0.727
iprobenfos	1	41.093	0.000	4	0	0.000	F	0.727
amobam	3	167.511	0.000	0	0	0.000	F	0.728
etridiazole	1	148.498	0.000	1	0	0.000	F	0.728
dimethirimol	1	0.000	1.788	4	2	4.762	F	0.728
tridemorph	1	0.000	0.000	4	0	12.082	F	0.729
thiophanate	1	90.384	0.000	2	4	6.447	F	0.729
fluopicolide	1	0.000	0.000	4	0	0.000	F	0.730
binapacryl	1	0.000	0.000	4	0	0.000	F	0.730
thiram	1	150.748	3.788	4	0	0.000	F	0.730
triclopyricarb	1	117.446	1.003	2	0	0.000	F	0.730
diclocymet	1	78.297	0.000	4	1	3.316	F	0.730
pydiflumetofen	1	117.446	0.990	3	0	0.000	F	0.730
fluazinam	1	78.297	0.000	0	4	2.000	F	0.730
metalaxyl	1	0.000	1.444	5	0	0.000	F	0.730
metalaxyl_m	1	0.000	1.444	5	0	0.000	F	0.730
propineb	2	182.598	0.000	1	0	2.000	F	0.731
fthalide	1	156.595	0.000	0	0	0.000	F	0.731
tecnazene	1	156.595	0.000	0	0	0.000	F	0.731
dichlofluanid	1	115.847	1.666	2	0	0.000	F	0.731
captan	1	149.427	0.995	0	0	0.000	F	0.731
furametpyr	1	39.149	0.000	5	3	3.827	F	0.731

tolylfluanid	1	115.847	1.686	3	0	0.000	F	0.731
quintozene	1	195.743	0.000	0	0	0.000	F	0.731
pyributicarb	1	45.192	1.710	5	0	0.000	F	0.731
metrafenone	1	0.000	0.000	6	0	0.000	F	0.731
tebufloquin	1	0.000	0.000	6	0	0.000	F	0.731
captafol	1	188.576	1.000	0	0	0.000	F	0.731
tebuconazole	1	391.486	0.000	0	0	0.000	F	0.731
oxolinic_acid	1	0.000	1.710	1	2	3.789	NF	0.505
carbendazim	1	0.000	0.000	0	0	4.000	NF	0.509
tricyclazole	1	31.278	0.000	1	0	0.000	NF	0.513
pyrimethanil_met	1	0.000	0.000	2	0	2.828	NF	0.518
boscalid	1	78.297	0.000	0	1	4.436	NF	0.538
proquinazid	1	0.000	1.633	2	0	0.000	NF	0.544
fluopyram	1	39.149	0.000	0	1	4.745	NF	0.547
cyclobutrifluram	1	78.297	0.000	0	1	4.690	NF	0.550
mepanipyrim_met	1	0.000	0.000	2	0	4.353	NF	0.551
thiabendazole	1	31.278	0.000	0	0	2.872	NF	0.552
fenpiclonil	1	78.297	0.000	0	1	4.757	NF	0.553
flumorph	1	0.000	1.744	2	0	0.000	NF	0.553
aldimorph	1	0.000	2.587	3	0	0.000	NF	0.553
tridemorph	1	0.000	2.590	3	0	0.000	NF	0.553
mefentrifluconazole	1	39.149	0.000	1	1	5.858	NF	0.554
flusulfamide	1	84.105	0.000	0	0	3.155	NF	0.557
benomyl	1	0.000	0.000	2	2	5.742	NF	0.570
metominostrobin	1	0.000	0.000	2	1	6.598	NF	0.606
dodemorph	1	0.000	2.741	2	0	0.000	NF	0.615
diclomezine	1	78.297	0.000	1	0	4.586	NF	0.637
methasulfocarb	1	39.969	0.000	2	0	2.872	NF	0.654
diclocymet	1	0.000	0.000	0	0	5.885	NF	0.655
fenhexamid	1	78.297	0.000	1	0	6.406	NF	0.687
mandipropamid	1	39.149	0.000	1	0	6.529	NF	0.687
ametoctradin	1	0.000	0.000	2	2	8.549	NF	0.688
prothiocarb	2	84.721	0.000	3	0	3.464	NF	0.714
fenamidone	1	32.220	1.535	2	0	5.686	NF	0.730
dodin	2	0.000	0.000	2	0	12.574	NF	0.731
**Inactive Group**								
91739	1	78.297	0.000	0	1	3.586	F	0.503
15706387	1	0.000	0.000	1	0	4.745	F	0.508
6	1	39.149	0.000	0	0	0.000	F	0.509
127151	1	0.000	0.000	1	1	5.326	F	0.511
10445549	1	0.000	0.000	0	3	3.780	F	0.515
211207	1	12.720	0.000	0	0	0.000	F	0.521
126970	1	39.149	1.950	1	0	0.000	F	0.521
115239	1	6.046	0.000	1	0	0.000	F	0.522
11339	1	1.339	0.000	4	2	6.089	F	0.525
2048	1	0.000	0.000	2	0	0.000	F	0.526
159599	1	0.000	0.000	3	0	5.743	F	0.527

214356	1	3.143	0.000	0	0	2.828	F	0.528
2583	1	0.000	0.000	3	2	7.129	F	0.530
1018	1	0.000	0.000	0	0	3.000	F	0.534
187238	1	0.000	0.000	3	2	7.242	F	0.534
2683	1	0.000	0.000	1	0	0.000	F	0.545
108137	1	0.000	0.000	1	0	0.000	F	0.545
219069	1	39.149	0.609	0	0	0.000	F	0.546
123619	1	45.195	0.000	2	0	0.000	F	0.552
14343	1	0.000	0.000	1	2	4.757	F	0.559
192737	1	0.000	0.000	0	0	1.000	F	0.559
14129	1	0.000	0.000	0	0	0.000	F	0.559
163797	1	0.000	1.490	2	2	3.568	F	0.560
91683	1	39.149	0.000	0	2	3.543	F	0.587
98895	1	0.000	0.000	1	0	2.971	F	0.587
460	1	0.000	0.000	1	0	2.828	F	0.588
2293	1	0.000	2.521	4	0	0.000	F	0.602
15965	1	117.446	0.000	0	1	3.780	F	0.605
129228	1	0.000	0.000	0	2	2.828	F	0.606
92357	1	38.810	1.076	2	0	0.000	F	0.607
10419733	1	64.325	0.000	1	0	0.000	F	0.611
15459	1	0.000	0.000	2	1	2.942	F	0.617
122067	1	5.807	0.000	0	1	2.000	F	0.621
2451	1	70.426	0.000	1	0	0.000	F	0.639
219095	1	39.149	0.000	3	1	6.586	F	0.645
11405965	1	39.149	1.328	3	0	0.000	F	0.677
1684	1	0.000	0.000	3	0	0.000	F	0.685
10848	1	78.297	2.222	1	0	0.000	F	0.689
992	1	195.743	0.000	0	0	2.872	F	0.730
91741	1	78.297	0.000	0	1	3.930	NF	0.515
2155	1	31.278	0.000	0	0	1.000	NF	0.527
12302171	1	0.000	0.000	0	10	9.873	NF	0.528
216249	1	44.956	0.000	2	1	5.451	NF	0.530
119114	1	31.278	0.000	0	1	4.586	NF	0.530
93379	1	39.149	0.000	0	1	4.456	NF	0.531
11210478	1	0.000	0.000	1	1	5.686	NF	0.541
10199199	1	39.149	0.000	3	0	4.350	NF	0.548
160557	1	60.363	0.000	0	0	2.000	NF	0.550
10245201	1	0.000	2.158	3	0	0.000	NF	0.554
10090	1	0.000	2.142	3	0	0.000	NF	0.554
13726	1	78.297	0.000	0	0	2.828	NF	0.555
194233	1	38.564	0.000	1	0	1.000	NF	0.560
196968	1	0.000	0.000	2	0	4.594	NF	0.561
78569	1	0.000	0.000	0	0	4.757	NF	0.562
227260	1	32.220	0.000	1	0	3.709	NF	0.564
127657	1	136.579	0.000	1	0	5.657	NF	0.569
104901	1	0.000	2.058	2	0	0.000	NF	0.570
10262683	1	60.363	0.000	1	0	2.000	NF	0.582

121940	1	32.220	1.504	2	0	1.755	NF	0.583
2562	1	0.000	2.314	2	0	0.000	NF	0.583
13738	1	0.000	2.340	2	0	0.000	NF	0.585
123920	1	78.297	0.000	0	0	3.864	NF	0.587
157839	1	31.278	0.000	0	0	4.327	NF	0.588
172309	1	0.000	3.040	3	0	0.000	NF	0.590
11236633	1	78.297	0.000	0	0	4.000	NF	0.591
11326715	1	0.000	0.000	1	1	6.041	NF	0.592
219104	1	60.363	0.000	2	0	1.890	NF	0.594
72154	1	110.517	0.000	1	0	4.711	NF	0.595
2081	1	39.149	0.000	3	0	5.451	NF	0.597
100413	1	39.149	0.000	0	0	4.649	NF	0.607
11960529	1	0.000	0.000	1	1	6.248	NF	0.618
10358610	1	78.297	0.000	1	1	6.334	NF	0.622
150949	1	39.149	0.000	0	0	5.201	NF	0.629
10275	1	31.278	0.000	0	0	5.329	NF	0.631
10331863	1	0.000	0.000	2	0	6.336	NF	0.637
134898	1	0.000	3.967	2	2	3.789	NF	0.637
108089	1	0.000	2.445	2	1	3.364	NF	0.643
2712	1	39.149	1.170	1	2	3.947	NF	0.645
172975	2	44.468	0.000	1	0	0.000	NF	0.646
15387	1	0.000	2.442	1	0	0.000	NF	0.655
10770	1	0.000	0.000	1	0	6.447	NF	0.661
160883	1	0.000	0.000	2	2	7.237	NF	0.662
159500	1	0.000	2.724	1	0	0.000	NF	0.664
2369	1	0.000	0.000	2	1	8.152	NF	0.666
194066	1	0.000	0.000	2	0	7.855	NF	0.667
2169	1	5.807	0.000	2	0	9.644	NF	0.674
10013505	1	32.220	2.331	2	1	2.586	NF	0.677
119574	1	39.149	2.484	1	3	5.970	NF	0.677
126094	1	0.000	0.000	1	8	14.794	NF	0.684
1858	1	39.149	0.000	2	0	5.640	NF	0.686
130305	1	0.000	0.000	6	2	7.173	NF	0.687
92425	1	0.000	2.138	0	0	0.000	NF	0.696
192251	2	52.501	0.000	3	0	0.000	NF	0.697
14683796	1	0.000	2.394	3	0	5.586	NF	0.698
10313100	2	44.468	0.000	2	0	1.000	NF	0.700
216468	1	50.764	0.000	0	0	7.098	NF	0.701
153103	1	0.000	1.018	1	3	7.237	NF	0.708
121596244	1	0.000	0.000	3	8	8.268	NF	0.709
173250	1	0.000	3.683	3	0	3.789	NF	0.711
1883	1	32.220	1.288	1	0	3.780	NF	0.715
10250769	1	32.220	1.439	3	0	5.164	NF	0.717
122737	1	0.000	4.135	2	1	6.727	NF	0.720
10130337	1	0.000	0.000	0	0	7.445	NF	0.720
192706	1	0.000	4.721	3	0	5.744	NF	0.721
11494412	1	0.000	0.000	0	0	7.669	NF	0.723

10718	1	0.000	0.000	0	3	8.200	NF	0.724
10547	2	41.093	0.000	5	0	0.000	NF	0.724
166553	1	39.149	2.633	1	0	2.730	NF	0.724
13770	2	44.468	2.427	1	0	0.000	NF	0.725
72076	1	0.000	0.000	0	0	7.855	NF	0.725
194680	1	78.297	0.000	1	0	8.606	NF	0.725
2790	1	71.368	0.000	0	0	9.000	NF	0.726
11623906	1	0.000	1.361	0	0	2.872	NF	0.726
93154	1	0.000	5.369	0	0	0.000	NF	0.727
92965	2	44.468	2.431	2	0	0.000	NF	0.727
14987	1	0.000	2.380	1	1	6.427	NF	0.728
2200	1	0.000	2.354	0	0	3.649	NF	0.728
160154	1	0.000	2.597	1	0	3.769	NF	0.728
11689883	1	0.000	3.675	2	0	5.329	NF	0.729
132260161	1	62.555	1.107	0	1	6.614	NF	0.729
92411	1	39.149	2.795	2	1	7.279	NF	0.730
73341	2	52.501	0.000	2	2	4.000	NF	0.730
11316914	1	117.446	3.774	0	1	5.154	NF	0.730
102669	1	0.000	2.314	2	0	5.744	NF	0.730
2040	1	0.000	1.317	1	0	4.745	NF	0.730
129791	2	44.468	2.068	4	0	2.889	NF	0.730
221227	1	0.000	0.000	0	2	9.421	NF	0.730
11465618	1	39.149	5.157	3	0	0.000	NF	0.730
11622909	1	0.000	1.919	2	0	5.586	NF	0.730
2448	1	0.000	2.297	0	1	7.585	NF	0.730
11984562	2	39.149	0.000	4	0	4.353	NF	0.731
101744	1	0.000	2.451	0	0	5.321	NF	0.731
11243969	1	0.000	1.804	0	0	4.848	NF	0.731
83813	2	83.617	2.060	2	1	3.790	NF	0.731
134819291	2	0.000	3.549	0	0	0.000	NF	0.731
164509	1	0.000	1.540	2	0	6.735	NF	0.731
14454445	2	44.468	0.000	3	0	7.159	NF	0.731
158789	1	0.000	0.000	0	2	10.819	NF	0.731
100516	1	0.000	0.000	0	3	12.715	NF	0.731
159598	2	44.468	0.000	3	0	5.743	NF	0.731
151178	3	14.527	0.000	3	0	0.000	NF	0.731
127873	2	2.496	0.000	1	8	5.526	NF	0.731
16046068	1	0.000	0.000	0	0	11.909	NF	0.731
12447	2	44.468	0.000	1	0	6.803	NF	0.731
117947705	1	39.149	1.907	0	2	15.839	NF	0.731
91268	2	0.000	2.369	2	0	0.000	NF	0.731
91614	3	105.003	0.000	0	1	7.237	NF	0.731
177358	5	105.003	0.000	1	0	0.000	NF	0.731
101673418	2	0.000	4.003	2	0	0.000	NF	0.731
107896	1	0.000	1.516	1	0	8.742	NF	0.731
152035	2	44.468	0.000	2	7	10.650	NF	0.731
122536283	1	0.000	1.156	0	0	8.911	NF	0.731

11427553	1	0.000	1.900	0	0	11.452	NF	0.731
159947	4	0.000	0.000	3	0	0.000	NF	0.731
16040217	5	4.992	0.000	0	16	0.000	NF	0.731

CID: compound ID; nStruct.: number of structures; Cl.: classification; F: predicted as Fungicide by the model; NF: predicted as non-Fungicide by the model; C.L.: confidence level of the classification provided by the model; P_VSA_m_4: Mass-weighted VSA. VdW surface area where atomic mass falls in bin 4; SsssN: E-State descriptor. E-State sum for quaternary nitrogens (N with 4 single bonds); NsCH3: Atom-centered fragment. Count of methyl groups (–CH_3) attached to sulfur (S); CATS2D_04_DA: CATS2D correlation vector. Donor-acceptor atom pairs separated by 4 bonds; SHED_DL: Shannon entropy descriptor. Entropy of drug-like atomic property distributions.

Table S6. Classification and probability of classification for training set compounds in Model 6 (ANN-AlvaDesc) trained with Pubchem-WIPO data, focusing on Fungicide activity prediction.

CID	MATS2p	SaasC	SsCl	NaaN	Class.	C.L.
**Active Group**						
16682942	-0.219	0.000	0.000	0	F	0.564
18771	0.061	0.698	0.000	0	F	0.688
11292824	0.050	3.388	5.913	0	F	0.696
39676	0.124	0.920	11.668	0	F	0.718
5455	0.509	0.000	0.000	0	F	0.726
9578570	0.223	0.500	0.000	0	F	0.729
24462	0.390	0.000	0.000	0	F	0.729
25429	0.142	0.387	0.000	1	F	0.730
3034285	0.175	1.928	0.000	2	F	0.730
17432	0.271	0.569	16.587	2	F	0.730
91699	0.131	-1.105	5.673	1	F	0.730
66461	0.073	1.695	12.164	2	F	0.730
86173	0.042	3.120	12.475	2	F	0.730
2730	0.154	0.551	17.487	1	F	0.730
8607	0.266	0.656	16.603	0	F	0.730
15910	-0.018	-0.273	22.812	0	F	0.730
39385	0.153	1.157	5.833	2	F	0.730
41368	0.127	1.240	5.850	2	F	0.730
86132	-0.002	1.563	5.950	2	F	0.730
10788	0.310	0.000	0.000	0	F	0.731
1810180	-0.019	0.000	0.000	2	F	0.731
11159	-0.233	0.000	0.000	0	F	0.742
50367	0.017	0.926	0.000	0	NF	0.572
32518	-0.161	0.000	0.000	0	NF	0.574
3032792	-0.064	1.097	0.000	0	NF	0.593
30154	0.186	0.000	0.000	0	NF	0.657
6327657	0.111	4.630	0.000	0	NF	0.708
**Inactive Group**						
7568320	-0.201	0.000	0.000	0	NF	0.552
3058751	-0.058	0.651	0.000	0	NF	0.568
9548842	-0.146	0.000	0.000	0	NF	0.578
15365	-0.140	0.000	0.000	0	NF	0.580
9838802	0.062	1.087	0.000	0	NF	0.584
66384	-0.073	1.173	0.000	0	NF	0.604
9571001	0.185	0.000	0.000	0	NF	0.656
82238	0.000	0.000	0.000	0	NF	0.677
23663941	0.009	1.409	0.000	0	NF	0.677
7215	0.199	0.000	0.000	0	NF	0.678
177335	-0.009	1.458	0.000	0	NF	0.681
61948	-0.050	0.000	0.000	0	NF	0.688
5483	-0.049	0.000	0.000	0	NF	0.688
65701	-0.079	2.412	0.000	1	NF	0.689
2955	0.044	1.496	0.000	0	NF	0.692

42510	0.208	2.548	0.000	0	NF	0.701
21720	0.049	1.657	0.000	0	NF	0.702
66368	-0.101	1.914	0.000	0	NF	0.705
7083	0.313	4.054	0.000	0	NF	0.708
657310	0.076	5.158	6.198	0	NF	0.708
9844338	-0.007	2.344	0.000	0	NF	0.708
65856	-0.088	2.615	0.000	0	NF	0.708
5387	0.159	3.663	0.000	0	NF	0.708
6917740	0.005	3.080	0.000	0	NF	0.708
5359271	0.126	4.427	0.000	0	NF	0.708
3036461	-0.020	5.935	0.000	0	NF	0.708
636970	-0.121	2.262	0.000	1	NF	0.710
64393	-0.040	0.000	0.000	0	NF	0.710
9813116	-0.008	0.000	0.000	0	NF	0.717
44158	-0.026	2.940	23.743	0	NF	0.718
25147683	-0.046	-4.659	0.000	0	NF	0.725
68889	-0.048	1.605	5.693	0	NF	0.726
216236	0.155	1.471	5.990	0	NF	0.726
10461	-0.336	0.000	10.747	0	NF	0.726
3034005	0.056	1.090	0.000	1	NF	0.728
120202	0.077	1.909	0.000	1	NF	0.733
171450	-0.022	0.000	0.000	0	NF	0.736

CID: compound ID; Class.: classification; F: predicted as Fungicide by the model; NF: predicted as non-Fungicide by the model; C.L.: confidence level of the classification provided by the model; MATS2p: Moran Autocorrelation (2D) descriptor (weighted by atomic polarizabilities); SaasC: E-State Atom-Type Descriptor (Electrotopological State). Sum of E-State values for carbon atoms (C) in the topological environment "aas"; SsCl: E-State Atom-Type Descriptor. Sum of E-State values for chlorine atoms (Cl) in the topological environment "s"; NaaN: Atom-Centered Fragment (ACF) Descriptor. Count of nitrogen atoms (N) in the topological environment "aaN".

Table S7. Classification and probability of classification for training set compounds in Model 7 (LDA-Mordred) trained with WIPO data, focusing on Fungicide activity prediction.

CID	ZMIC2	Lipinski	n6HRing	DF	Class.	C.L.
**Active Group**						
59649244	32.780	0	0	-2.460	NF	0.920
10788	21.510	1	1	-1.582	NF	0.829
9257	13.500	1	0	-1.390	NF	0.800
7156993	16.348	1	0	-1.108	NF	0.751
11048796	36.379	1	2	-1.096	NF	0.747
30154	21.738	1	0	-0.574	NF	0.638
5366415	22.491	1	0	-0.499	NF	0.620
33112	22.542	1	0	-0.494	NF	0.619
92200	43.046	1	2	-0.436	NF	0.604
50367	33.733	1	1	-0.372	NF	0.589
5430	24.106	1	0	-0.340	NF	0.582
28780	24.556	1	0	-0.295	NF	0.571
86132	24.719	1	0	-0.279	NF	0.567
23690429	25.000	1	0	-0.251	NF	0.560
11953884	66.107	0	1	-0.146	NF	0.531
135083	36.722	1	1	-0.077	NF	0.516
5634	27.470	1	0	-0.006	F	0.501
6950	27.851	1	0	0.031	F	0.510
22321033	28.169	1	0	0.063	F	0.518
41368	28.265	1	0	0.072	F	0.520
66461	28.841	1	0	0.129	F	0.535
1493	28.853	1	0	0.130	F	0.535
6191	28.853	1	0	0.130	F	0.535
17776	28.916	1	0	0.137	F	0.537
39385	28.950	1	0	0.140	F	0.537
7744	59.130	0	0	0.149	F	0.542
6451142	29.697	1	0	0.214	F	0.556
2730	39.834	1	1	0.232	F	0.561
91699	30.081	1	0	0.252	F	0.565
4921319	30.140	1	0	0.258	F	0.567
7223	60.310	0	0	0.266	F	0.571
9492	30.327	1	0	0.276	F	0.571
11159	60.478	0	0	0.282	F	0.575
122087	30.699	1	0	0.313	F	0.580
7430	30.750	1	0	0.318	F	0.581
6112114	30.832	1	0	0.326	F	0.583
11292824	30.982	1	0	0.341	F	0.587
10935908	31.089	1	0	0.352	F	0.590
6437379	31.323	1	0	0.375	F	0.595
6422843	31.344	1	0	0.377	F	0.596
39676	31.666	1	0	0.409	F	0.603
14994	32.124	1	0	0.454	F	0.614
17581	32.166	1	0	0.458	F	0.615

3037	32.874	1	0	0.528	F	0.632
86173	33.436	1	0	0.584	F	0.645
16682936	33.849	1	0	0.625	F	0.654
45380430	34.653	1	0	0.705	F	0.672
16682983	35.255	1	0	0.764	F	0.685
12318	35.345	1	0	0.773	F	0.687
18771	45.340	1	1	0.777	F	0.688
11664966	36.195	1	0	0.857	F	0.705
213016	36.195	1	0	0.857	F	0.705
9578570	36.195	1	0	0.857	F	0.705
24462	36.293	1	0	0.867	F	0.707
14309	36.688	1	0	0.906	F	0.715
16682942	36.769	1	0	0.914	F	0.716
16684215	37.537	1	0	0.990	F	0.732
3647458	37.795	1	0	1.016	F	0.737
11486133	37.927	1	0	1.029	F	0.739
72980153	37.927	1	0	1.029	F	0.739
124962	69.549	0	0	1.180	F	0.769
5460680	39.595	1	0	1.194	F	0.770
10598	40.253	1	0	1.259	F	0.781
17432	40.924	1	0	1.325	F	0.792
1730	41.000	1	0	1.333	F	0.794
56840815	41.181	1	0	1.351	F	0.797
54682462	41.553	1	0	1.388	F	0.803
213032	41.810	1	0	1.413	F	0.807
7627	42.376	1	0	1.469	F	0.815
90545	42.608	1	0	1.492	F	0.819
17110	42.896	1	0	1.521	F	0.823
16682924	43.023	1	0	1.533	F	0.825
3032792	43.095	1	0	1.540	F	0.826
5455	43.227	1	0	1.553	F	0.828
65258	43.250	1	0	1.556	F	0.828
7511	44.173	1	0	1.647	F	0.841
25202562	44.700	1	0	1.699	F	0.847
3032581	45.752	1	0	1.803	F	0.860
8606	46.703	1	0	1.898	F	0.871
8607	50.322	1	0	2.256	F	0.907
15910	56.881	1	0	2.905	F	0.949
6327657	58.728	1	0	3.088	F	0.957
23663539	64.386	1	0	3.648	F	0.975
6720	64.816	1	0	3.691	F	0.976
**Inactive Group**						
219095	30.438	0	1	-3.678	NF	0.975
133128	32.797	0	1	-3.444	NF	0.969
216239	38.613	0	1	-2.868	NF	0.946
115223	29.677	1	3	-2.746	NF	0.939
11316914	40.950	0	1	-2.637	NF	0.932

1727	14.756	1	1	-2.251	NF	0.904
104781	35.692	1	3	-2.150	NF	0.895
232487	16.128	1	1	-2.115	NF	0.892
154257	36.617	0	0	-2.080	NF	0.888
114681	17.085	1	1	-2.021	NF	0.882
457	17.385	1	1	-1.991	NF	0.879
1018	18.018	1	1	-1.928	NF	0.872
14454445	38.693	0	0	-1.874	NF	0.865
1050	19.209	1	1	-1.810	NF	0.859
13985	40.326	0	0	-1.713	NF	0.845
100516	30.262	1	2	-1.702	NF	0.844
10016922	22.485	1	1	-1.486	NF	0.814
222284	42.620	0	0	-1.486	NF	0.813
168045	22.528	1	1	-1.482	NF	0.814
10047015	32.826	1	2	-1.448	NF	0.808
1456	23.882	1	1	-1.348	NF	0.792
15706387	24.512	1	1	-1.285	NF	0.782
1455	45.912	0	0	-1.160	NF	0.758
78165	26.559	1	1	-1.083	NF	0.745
123983	26.560	1	1	-1.083	NF	0.745
89124	16.608	1	0	-1.082	NF	0.746
1720	26.940	1	1	-1.045	NF	0.738
10313100	16.999	1	0	-1.043	NF	0.738
194233	17.108	1	0	-1.032	NF	0.736
76962	17.207	1	0	-1.022	NF	0.734
97663	18.552	1	0	-0.889	NF	0.707
1140	18.645	1	0	-0.880	NF	0.706
14777	18.806	1	0	-0.864	NF	0.702
130229	28.909	1	1	-0.850	NF	0.698
996	19.124	1	0	-0.833	NF	0.696
10176082	19.358	1	0	-0.810	NF	0.691
92411	29.652	1	1	-0.776	NF	0.683
73801	29.835	1	1	-0.758	NF	0.679
227	20.034	1	0	-0.743	NF	0.676
159247	20.372	1	0	-0.709	NF	0.669
112056	20.410	1	0	-0.705	NF	0.668
1646	20.739	1	0	-0.673	NF	0.661
1893	20.800	1	0	-0.667	NF	0.659
93379	30.870	1	1	-0.656	NF	0.656
1864	21.253	1	0	-0.622	NF	0.649
10783	21.292	1	0	-0.618	NF	0.648
151230	21.375	1	0	-0.610	NF	0.646
92189	21.494	1	0	-0.598	NF	0.644
10419733	21.678	1	0	-0.580	NF	0.639
2557	21.877	1	0	-0.560	NF	0.635
12130	21.903	1	0	-0.558	NF	0.634
999	21.914	1	0	-0.557	NF	0.634

72064	32.095	1	1	-0.535	NF	0.628
10007	22.162	1	0	-0.532	NF	0.628
13643	22.409	1	0	-0.507	NF	0.622
110992	22.595	1	0	-0.489	NF	0.618
78782	22.858	1	0	-0.463	NF	0.612
73343	23.688	1	0	-0.381	NF	0.592
127151	33.814	1	1	-0.364	NF	0.587
77139	24.083	1	0	-0.342	NF	0.583
2075	24.468	1	0	-0.304	NF	0.573
2078	25.696	1	0	-0.182	NF	0.543
11033	25.721	1	0	-0.180	NF	0.543
101209015	25.891	1	0	-0.163	NF	0.538
123805	56.643	0	0	-0.097	NF	0.519
216249	26.883	1	0	-0.065	NF	0.514
15402	27.320	1	0	-0.021	NF	0.503
119569	27.719	1	0	0.018	F	0.507
13676	28.416	1	0	0.087	F	0.524
72300	28.747	1	0	0.120	F	0.532
170317	29.708	1	0	0.215	F	0.556
2708	30.485	1	0	0.292	F	0.575
198707	30.624	1	0	0.306	F	0.578
168884	41.623	1	1	0.409	F	0.604
82178	31.754	1	0	0.418	F	0.606
6	31.858	1	0	0.428	F	0.608
2789	32.147	1	0	0.457	F	0.615
11001318	34.003	1	0	0.640	F	0.657
11425584	34.093	1	0	0.649	F	0.659
15532	34.250	1	0	0.665	F	0.663
101815861	37.601	1	0	0.997	F	0.733
121957	43.015	1	0	1.533	F	0.825
12620	76.665	0	0	1.885	F	0.871
10903489	47.639	1	0	1.990	F	0.881
88842	55.183	1	0	2.737	F	0.940
10350985	100.605	0	0	4.255	F	0.986

CID: compound ID; Cl.: classification; DF: discriminant function; C.L.: confidence level of the classification provided by the model; ZMIC2: Zagreb Index (modified). Topological index emphasizing heteroatoms/bonds.; Lipinski: Drug-likeness rule. Binary flag for Rule of Five compliance; n6HRing: Heterocycle count. Number of 6-membered rings with heteroatoms.

Table S8. Classification and probability of classification for training set compounds in Model 8 (LDA-Mordred) trained with FRAC-WIPO data, focusing on Fungicide activity prediction.

CID	GATS3c	NssCH2	PEOE_VSA6	VSA_EState6	DF	Class.	C.L.
**Active Group**							
flumorph	0.923	4	18.199	11.510	-2.089	NF	0.890
oxathiapiprolin	1.075	6	11.222	4.564	-1.817	NF	0.860
zineb	0.166	2	8.641	0.000	-1.709	NF	0.847
ziram	0.066	0	4.321	0.000	-1.486	NF	0.815
prothiocarb	0.352	4	18.686	0.000	-1.382	NF	0.799
propineb	0.176	1	8.641	0.168	-1.360	NF	0.796
laminarin	0.809	3	0.000	0.000	-1.292	NF	0.785
febram	0.193	0	4.321	0.000	-1.222	NF	0.772
flutianil	0.826	2	23.895	11.431	-1.193	NF	0.767
oxolinic_acid	0.963	2	0.000	3.243	-1.140	NF	0.758
validamycin	0.744	4	6.076	-1.935	-1.075	NF	0.745
pyraclostrobin	0.983	1	29.800	16.283	-0.911	NF	0.713
propamocarb	1.141	5	6.924	0.000	-0.866	NF	0.704
sedaxane	1.054	3	18.199	7.681	-0.841	NF	0.699
mandipropamid	0.881	4	41.641	12.417	-0.798	NF	0.690
fludioxonil	0.680	0	12.133	6.512	-0.753	NF	0.680
tavaborole	0.885	1	6.066	4.241	-0.713	NF	0.671
thiabendazole	0.877	0	12.133	7.971	-0.581	NF	0.641
prothioconazole	0.989	4	29.800	7.482	-0.546	NF	0.633
myclobutanil	0.779	4	43.499	10.009	-0.498	NF	0.622
ofurace	1.027	3	18.199	5.185	-0.493	NF	0.621
methasulfocarb	1.169	0	0.000	6.208	-0.479	NF	0.617
mefentrifluconazole	1.117	1	17.667	9.659	-0.351	NF	0.587
amisulbrom	1.043	0	0.000	3.717	-0.338	NF	0.584
blasticidin_s	1.094	3	6.076	-0.070	-0.294	NF	0.573
metconazole	0.909	4	37.581	7.911	-0.275	NF	0.568
edifenphos	1.335	1	36.398	19.419	-0.257	NF	0.564
cyproconazole	0.926	3	30.657	7.446	-0.257	NF	0.564
proquinazid	1.530	4	13.847	6.125	-0.240	NF	0.560
thifluzamide	0.968	0	0.000	1.826	-0.188	NF	0.547
diethofencarb	1.577	2	0.000	5.227	-0.185	NF	0.546
difenoconazole	1.424	2	23.202	12.535	-0.175	NF	0.544
triflumizole	1.046	3	18.525	3.467	-0.156	NF	0.539
flutriafol	0.944	1	30.332	11.247	-0.144	NF	0.536
furalaxyl	0.914	0	18.199	8.157	-0.139	NF	0.535
pencycuron	1.391	5	54.774	17.617	-0.080	NF	0.520
cyprodinil	1.612	2	18.199	12.122	-0.043	NF	0.511
benodanil	1.266	0	30.332	16.962	-0.042	NF	0.510
mandestrobin	1.004	1	36.398	13.792	-0.036	NF	0.509
picoxystrobin	0.931	1	30.332	10.135	0.009	F	0.502
pyraziflumid	0.877	0	24.265	9.141	0.020	F	0.505
isopyrazam	0.923	2	25.980	5.982	0.025	F	0.506

ethirimol	1.198	4	13.345	0.000	0.028	F	0.507
metalaxyl	0.932	1	18.199	5.011	0.050	F	0.512
metalaxyl	0.932	1	18.199	5.011	0.050	F	0.512
cyclobutrifluram	0.938	2	29.268	7.222	0.068	F	0.517
fluopyram	0.937	2	23.734	4.958	0.074	F	0.518
diclomezine	0.747	0	23.202	6.562	0.097	F	0.524
azoxystrobin	1.357	0	30.332	17.197	0.109	F	0.527
dimethirimol	1.079	3	13.345	0.000	0.138	F	0.534
carboxin	1.496	2	18.199	9.422	0.152	F	0.538
thiophanate	1.496	2	12.133	6.966	0.154	F	0.538
ametoctradin	0.926	8	45.952	0.000	0.159	F	0.540
tolprocarb	0.846	2	31.544	6.365	0.164	F	0.541
tricyclazole	0.753	0	23.470	6.289	0.170	F	0.542
pyrazophos	1.645	3	0.000	1.612	0.184	F	0.546
ipflufenoquin	1.448	0	6.066	8.089	0.191	F	0.548
fluxapyroxad	1.033	0	18.199	7.491	0.215	F	0.554
iodocarb	1.120	4	19.265	0.000	0.252	F	0.563
epoxiconazole	0.986	1	41.933	13.932	0.264	F	0.566
imibenconazole	0.929	2	46.936	12.996	0.267	F	0.566
mattifine	0.732	2	84.948	25.566	0.297	F	0.574
hymexazole	1.170	0	0.000	1.389	0.303	F	0.575
polyoxin	0.964	0	0.000	-1.257	0.304	F	0.575
dodin	0.925	11	64.711	0.000	0.307	F	0.576
isofetamid	1.640	0	0.000	7.307	0.322	F	0.580
spiroxamine	1.531	9	34.619	0.000	0.322	F	0.580
cinnamaldehyde	0.687	0	36.408	9.702	0.325	F	0.580
pyrametostrobin	1.073	1	48.531	17.286	0.332	F	0.582
pyraoxystrobin	1.055	1	47.999	16.737	0.348	F	0.586
metominostrobin	1.257	0	35.488	16.496	0.349	F	0.586
dodemorph	1.578	13	57.787	0.822	0.367	F	0.591
benthiavalicarb	0.910	0	13.847	3.068	0.392	F	0.597
fluoxastrobin	1.202	2	41.022	13.324	0.395	F	0.597
fenpiclonil	0.581	0	35.335	7.437	0.401	F	0.599
pyrisoxazole	1.281	1	29.800	11.909	0.415	F	0.602
penconazole	0.740	3	42.613	5.678	0.419	F	0.603
streptomycin	0.983	1	0.000	-3.953	0.421	F	0.604
simeconazole	0.997	2	31.774	6.724	0.435	F	0.607
thiram	0.362	0	24.436	0.000	0.439	F	0.608
oxytetracycline	0.948	0	12.133	2.537	0.446	F	0.610
dimoxystrobin	1.059	1	41.554	13.568	0.450	F	0.611
triticonazole	0.895	3	43.657	7.669	0.487	F	0.619
pyrifenox	0.769	1	40.490	9.147	0.492	F	0.621
metrafenone	1.075	0	15.930	5.366	0.499	F	0.622
octhilinone	0.911	7	50.560	1.641	0.519	F	0.627
kresoxim-methyl	1.024	1	47.620	15.114	0.523	F	0.628
fenfuram	1.454	0	18.199	10.989	0.524	F	0.628
benomyl	1.422	3	25.477	6.833	0.535	F	0.631

flusulfamide	0.838	0	23.202	4.993	0.539	F	0.632
azaconazole	1.182	3	29.268	5.246	0.540	F	0.632
thiophanate methyl	1.348	0	12.133	6.915	0.569	F	0.639
biphenyl	0.911	0	60.664	20.781	0.576	F	0.640
oxpoconazole	1.746	4	23.734	7.788	0.582	F	0.642
fenpropidin	1.307	7	58.381	9.270	0.616	F	0.649
tolfenpyrad	1.251	2	48.354	15.496	0.622	F	0.651
oxycarboxin	1.669	2	18.199	8.681	0.631	F	0.653
pyrimorph	1.089	4	56.638	12.024	0.674	F	0.662
carbendazim	1.446	0	12.133	7.507	0.677	F	0.663
amobam	0.841	2	24.436	0.000	0.720	F	0.673
trifloxystrobin	0.875	1	46.710	11.545	0.730	F	0.675
phenamacril	1.316	1	30.332	10.608	0.732	F	0.675
binapacryl	0.992	1	19.421	2.017	0.738	F	0.676
fluindapyr	1.014	1	20.771	2.799	0.746	F	0.678
tebuconazole	1.012	3	44.505	7.761	0.772	F	0.684
fenhexamid	0.854	5	49.388	2.987	0.819	F	0.694
quinofumelin	1.250	0	42.465	16.287	0.823	F	0.695
benalaxyl-m	1.109	1	48.531	14.652	0.832	F	0.697
pyriofenone	1.100	0	11.601	1.717	0.860	F	0.703
acibenzolar-s-methyl	1.062	0	22.316	5.490	0.867	F	0.704
bupirimate	1.602	4	13.345	0.000	0.868	F	0.704
aldimorph	1.540	13	64.711	0.000	0.874	F	0.705
fluquinconazole	1.271	0	23.202	8.483	0.874	F	0.706
imazalil	0.998	2	35.344	5.410	0.882	F	0.707
penthiopyrad	1.004	1	20.771	1.721	0.899	F	0.711
tridemorph	1.528	14	71.132	0.000	0.910	F	0.713
mepronil	1.770	0	24.265	14.948	0.936	F	0.718
fenazaquin	1.221	2	57.169	16.673	0.944	F	0.720
terbinafine	1.005	2	60.381	15.073	0.963	F	0.724
thiazole	1.393	0	0.000	0.000	0.991	F	0.729
cymoxanil	1.487	1	5.156	0.799	1.035	F	0.738
inpyrfluxam	1.008	1	32.904	5.793	1.038	F	0.738
probenazole	1.345	1	24.788	6.578	1.083	F	0.747
pyrimethanil	1.797	0	18.199	11.863	1.096	F	0.750
boscalid	1.193	0	53.534	18.295	1.099	F	0.750
pyributicarb	1.280	0	38.970	13.470	1.113	F	0.753
etaconazole	1.250	3	36.192	5.297	1.123	F	0.755
propiconazole	1.231	4	42.613	5.322	1.140	F	0.758
ferimzone	1.493	0	24.265	10.097	1.144	F	0.758
fenarimol	0.943	0	53.534	14.134	1.255	F	0.778
pyridachlometyl	1.033	0	47.999	12.906	1.279	F	0.782
quinoxyfen	1.658	0	23.202	10.793	1.306	F	0.787
fluoroimide	1.198	0	23.202	4.863	1.309	F	0.787
chinomethionat	1.112	0	28.740	5.917	1.320	F	0.789
isotianil	1.135	0	35.335	8.659	1.353	F	0.795
dimethachlone	1.552	2	23.202	4.654	1.364	F	0.796

phosphorous acid	1.574	0	0.000	0.000	1.366	F	0.797
fenamidone	1.203	0	60.293	19.291	1.400	F	0.802
picarbutrazox	1.634	1	41.554	14.654	1.470	F	0.813
fluazinam	0.923	0	23.202	0.301	1.476	F	0.814
chlozolinate	1.406	1	23.202	4.177	1.496	F	0.817
mepanipyrim	1.800	0	24.120	11.701	1.513	F	0.819
triadimenol	1.176	0	32.372	6.973	1.519	F	0.820
zoxamide	1.294	2	30.126	3.054	1.538	F	0.823
prochloraz	1.277	4	41.727	2.969	1.560	F	0.826
dichlofluanid	0.993	0	41.401	7.965	1.565	F	0.827
chloroneb	1.196	0	23.202	3.228	1.569	F	0.828
fosetyl-al	2.202	3	0.000	0.000	1.601	F	0.832
dichlobentiazox	1.264	1	35.335	6.477	1.619	F	0.835
tebufloquin	1.307	0	20.771	3.359	1.621	F	0.835
procymidone	1.524	1	23.202	4.739	1.649	F	0.839
orysastrobin	1.037	1	44.888	7.154	1.658	F	0.840
fluopicolide	1.030	1	40.869	5.311	1.681	F	0.843
bitertanol	1.148	0	63.236	17.981	1.688	F	0.844
isoprothiolane	2.077	2	0.000	0.000	1.698	F	0.845
triadimefon	1.277	0	32.372	6.831	1.751	F	0.852
captafol	1.014	2	35.354	0.000	1.791	F	0.857
fenpicoxamid	1.187	3	58.027	9.032	1.808	F	0.859
tolylfluanid	1.015	0	40.898	6.499	1.815	F	0.860
silthiofam	1.159	1	25.717	0.000	1.822	F	0.861
tiadinil	1.525	0	22.156	5.386	1.836	F	0.862
penflufen	1.363	1	38.970	7.689	1.864	F	0.866
cyazofamid	1.231	0	41.430	8.896	1.911	F	0.871
fenpyrazamine	1.262	1	42.103	7.332	1.916	F	0.872
valifenalate	1.252	1	37.581	5.356	1.920	F	0.872
iprobenfos	1.847	1	30.332	9.897	1.950	F	0.875
triclopyricarb	1.022	1	53.002	8.420	1.950	F	0.875
natamycin	1.127	5	48.608	-1.077	1.992	F	0.880
carpropamid	1.479	1	37.581	7.304	2.076	F	0.889
furametpyr	1.662	0	23.734	5.851	2.148	F	0.895
pydiflumetofen	1.232	1	34.803	2.521	2.156	F	0.896
diniconazole	0.814	0	50.039	5.218	2.200	F	0.900
vinclozoline	1.418	0	29.781	4.425	2.265	F	0.906
etridiazole	1.103	1	34.803	0.000	2.296	F	0.909
fthalide	0.803	1	46.404	0.000	2.428	F	0.919
captan	1.019	2	46.955	0.000	2.556	F	0.928
iprovalicarb	1.368	0	43.676	7.240	2.610	F	0.932
triforine	0.876	4	69.606	0.000	3.020	F	0.953
tecnazene	1.082	0	46.404	1.251	3.161	F	0.959
diclocymet	1.528	0	50.039	6.892	3.413	F	0.968
quintozene	1.066	0	58.005	0.000	4.085	F	0.983
tecloftalam	0.923	0	75.672	4.538	4.204	F	0.985
**Inactive Group**							

134819291	0.144	9	0.000	0.000	-4.813	NF	0.992
101673418	0.213	9	0.000	0.000	-4.669	NF	0.991
2293	1.118	10	24.265	12.671	-3.615	NF	0.974
119593	0.739	7	0.000	2.067	-3.197	NF	0.961
11243969	0.911	8	0.000	1.722	-3.141	NF	0.959
15938	0.092	0	0.000	8.481	-3.085	NF	0.956
91614	0.307	2	5.156	8.756	-3.060	NF	0.955
11623906	0.843	7	0.000	1.673	-2.918	NF	0.949
107896	0.880	3	0.000	10.921	-2.911	NF	0.948
15387	1.050	7	6.924	5.832	-2.710	NF	0.938
194066	0.539	6	12.990	3.858	-2.702	NF	0.937
10939	0.206	3	0.000	0.000	-2.544	NF	0.927
160154	0.609	6	25.123	8.625	-2.538	NF	0.927
10547	0.428	4	0.000	0.000	-2.440	NF	0.920
2448	1.140	7	28.063	13.582	-2.401	NF	0.917
177358	0.041	0	6.066	5.856	-2.370	NF	0.915
73341	0.422	3	12.990	6.105	-2.238	NF	0.904
129791	0.970	3	12.133	12.794	-2.237	NF	0.904
11291932	1.027	6	24.265	11.798	-2.237	NF	0.904
93154	1.371	7	36.398	18.794	-2.221	NF	0.902
12717441	1.517	6	0.000	7.825	-2.155	NF	0.896
100580	0.799	6	12.990	3.768	-2.146	NF	0.895
14683796	1.034	6	6.066	3.936	-2.136	NF	0.894
80084	0.343	4	60.664	20.981	-2.061	NF	0.887
161240	0.770	5	18.199	7.041	-2.040	NF	0.885
122536283	0.875	3	6.066	7.784	-2.019	NF	0.883
92425	0.995	6	5.920	2.367	-1.972	NF	0.878
164509	0.825	2	12.133	11.373	-1.952	NF	0.876
185236	1.101	5	0.000	3.337	-1.939	NF	0.874
115239	0.849	0	0.000	11.091	-1.933	NF	0.874
11326715	1.100	1	0.000	11.963	-1.910	NF	0.871
11689883	1.113	5	12.133	7.861	-1.856	NF	0.865
192251	0.299	11	76.489	9.669	-1.790	NF	0.857
14129	0.679	1	12.133	10.465	-1.751	NF	0.852
119574	1.006	6	11.601	3.409	-1.749	NF	0.852
13320	0.492	2	42.465	17.795	-1.708	NF	0.847
127873	0.699	2	0.000	3.295	-1.697	NF	0.845
100516	0.823	4	12.133	5.350	-1.695	NF	0.845
158789	0.841	7	31.395	6.439	-1.649	NF	0.839
122737	0.803	3	12.133	6.928	-1.635	NF	0.837
134898	1.186	5	0.000	2.404	-1.610	NF	0.833
15939	0.806	0	0.000	8.481	-1.600	NF	0.832
119114	1.221	5	24.265	12.510	-1.592	NF	0.831
101744	0.875	4	24.275	10.270	-1.592	NF	0.831
72076	1.074	6	49.240	18.297	-1.565	NF	0.827
151178	0.351	0	0.000	2.207	-1.532	NF	0.822
2267	1.128	6	41.933	15.831	-1.530	NF	0.822

2369	1.223	7	25.980	8.414	-1.527	NF	0.822
11960529	0.933	3	6.066	5.436	-1.518	NF	0.820
11427553	0.972	4	36.408	15.790	-1.495	NF	0.817
2169	0.891	3	24.265	12.058	-1.493	NF	0.816
152035	1.007	5	12.133	4.168	-1.477	NF	0.814
1684	0.811	3	10.313	5.228	-1.462	NF	0.812
12302171	0.829	5	0.000	-3.291	-1.432	NF	0.807
14987	0.732	3	18.209	6.922	-1.387	NF	0.800
10178705	0.981	7	18.022	1.132	-1.370	NF	0.797
72103	0.644	3	13.345	3.485	-1.330	NF	0.791
16046068	0.937	3	30.332	14.067	-1.326	NF	0.790
2200	1.100	4	48.531	21.077	-1.294	NF	0.785
159500	1.175	5	18.209	7.599	-1.289	NF	0.784
119198	0.876	2	6.924	5.743	-1.275	NF	0.782
11105	1.078	0	0.000	9.962	-1.275	NF	0.782
132260161	0.908	2	0.000	3.286	-1.262	NF	0.779
2583	1.256	4	6.066	5.594	-1.230	NF	0.774
117947705	0.919	6	47.999	13.588	-1.208	NF	0.770
221227	0.867	0	36.398	21.419	-1.198	NF	0.768
78569	0.744	1	12.133	7.693	-1.169	NF	0.763
130305	0.911	2	0.000	2.465	-1.121	NF	0.754
159599	1.169	3	0.000	3.513	-1.112	NF	0.753
159598	1.174	3	0.000	3.513	-1.102	NF	0.751
14454445	0.777	0	18.209	12.211	-1.079	NF	0.746
157839	0.777	2	18.199	7.782	-1.076	NF	0.746
192706	1.144	2	25.123	15.260	-1.073	NF	0.745
1018	0.602	0	6.066	4.755	-1.027	NF	0.736
72413	0.949	3	0.000	0.000	-1.001	NF	0.731
98895	0.935	0	0.000	6.286	-0.977	NF	0.727
10419733	1.135	4	0.000	0.000	-0.972	NF	0.726
1883	0.580	1	0.000	-0.539	-0.968	NF	0.725
187238	1.213	2	6.066	7.758	-0.956	NF	0.722
2562	1.175	10	57.021	10.083	-0.947	NF	0.721
172309	1.163	3	6.066	4.660	-0.915	NF	0.714
108137	1.117	2	30.332	16.009	-0.910	NF	0.713
126970	1.133	4	17.667	6.603	-0.894	NF	0.710
127151	1.245	4	42.465	17.991	-0.889	NF	0.709
11494412	0.976	3	36.398	14.227	-0.878	NF	0.706
134391533	1.101	1	11.223	10.097	-0.875	NF	0.706
13268	1.183	4	0.000	0.000	-0.872	NF	0.705
15459	1.156	2	6.066	6.026	-0.794	NF	0.689
2683	0.795	15	90.040	6.315	-0.760	NF	0.681
12447	0.828	0	30.341	15.680	-0.744	NF	0.678
150949	0.788	1	11.601	5.375	-0.738	NF	0.677
208947	1.145	3	18.199	8.197	-0.735	NF	0.676
10770	1.180	6	37.462	9.757	-0.731	NF	0.675
10718	0.966	0	18.199	12.429	-0.721	NF	0.673

15907	1.096	2	11.601	7.012	-0.719	NF	0.672
10013505	1.219	5	6.924	0.000	-0.702	NF	0.669
10445549	0.680	1	0.000	-1.084	-0.673	NF	0.662
92266	0.873	0	0.000	3.510	-0.658	NF	0.659
192737	0.948	2	0.000	0.000	-0.647	NF	0.656
1930	0.805	14	70.442	0.000	-0.638	NF	0.654
460	0.768	0	12.133	6.838	-0.624	NF	0.651
163797	1.398	2	0.000	5.645	-0.624	NF	0.651
2040	1.168	1	18.199	12.165	-0.617	NF	0.650
159947	0.138	15	97.821	0.000	-0.597	NF	0.645
2391	1.292	0	30.332	20.485	-0.559	NF	0.636
219095	1.271	1	17.667	12.887	-0.553	NF	0.635
214356	1.009	2	6.066	2.600	-0.545	NF	0.633
160883	1.123	6	43.676	10.285	-0.530	NF	0.630
11683556	1.231	0	0.000	7.291	-0.525	NF	0.628
108089	1.215	3	17.696	7.472	-0.505	NF	0.624
172975	1.418	3	12.133	7.774	-0.494	NF	0.621
10331863	0.935	0	11.987	8.037	-0.481	NF	0.618
12056759	0.714	2	19.056	3.480	-0.456	NF	0.612
216239	1.246	0	11.601	11.597	-0.435	NF	0.607
194680	0.926	3	23.202	5.460	-0.423	NF	0.604
92357	0.898	1	0.000	0.000	-0.394	NF	0.597
2790	0.898	4	35.496	7.643	-0.389	NF	0.596
104901	0.891	0	24.778	11.899	-0.365	NF	0.590
82143	1.074	0	36.398	18.908	-0.361	NF	0.589
100413	0.792	1	17.667	5.446	-0.345	NF	0.585
11622909	1.279	4	30.332	10.087	-0.330	NF	0.582
10130337	0.899	2	30.332	9.571	-0.322	NF	0.580
216249	1.175	3	11.601	3.307	-0.312	NF	0.577
10783	1.118	2	0.000	0.000	-0.294	NF	0.573
11316914	1.132	6	53.356	12.683	-0.269	NF	0.567
15851	0.932	4	36.221	7.616	-0.268	NF	0.567
13770	0.771	4	66.256	17.615	-0.265	NF	0.566
1858	1.074	2	11.601	3.759	-0.237	NF	0.559
216468	0.813	0	23.734	9.627	-0.227	NF	0.556
15706387	1.156	4	36.753	10.217	-0.189	NF	0.547
10124	0.937	0	11.337	5.996	-0.189	NF	0.547
92965	1.128	6	43.173	7.927	-0.171	NF	0.543
129228	0.937	1	11.280	3.555	-0.154	NF	0.539
6	0.725	0	11.601	3.051	-0.135	NF	0.534
182137	1.007	4	36.221	7.616	-0.112	NF	0.528
102669	1.279	6	44.179	9.860	-0.105	NF	0.526
153103	0.993	2	6.924	0.000	-0.102	NF	0.525
1533	1.159	0	0.000	3.746	-0.100	NF	0.525
219069	0.766	1	41.933	13.141	-0.065	NF	0.516
123619	1.114	0	23.734	12.378	-0.047	NF	0.512
150315	0.726	1	24.265	5.355	-0.037	NF	0.509

187888	0.824	1	30.332	8.809	0.000	F	0.500
122067	1.076	0	0.000	1.948	0.018	F	0.504
2048	1.095	0	30.332	14.332	0.028	F	0.507
123920	0.834	3	29.268	3.929	0.028	F	0.507
76962	0.888	1	6.924	0.000	0.035	F	0.509
92411	1.217	4	25.448	5.065	0.037	F	0.509
10848	1.576	5	6.924	0.000	0.039	F	0.510
11465618	1.517	4	47.999	17.889	0.052	F	0.513
164867	1.031	0	11.601	5.812	0.053	F	0.513
166553	1.239	2	18.525	6.825	0.060	F	0.515
10199199	0.964	4	44.002	9.059	0.073	F	0.518
194233	1.320	2	0.000	0.000	0.126	F	0.531
2712	1.032	1	41.933	15.107	0.169	F	0.542
10245201	1.384	4	25.980	6.555	0.176	F	0.544
91452	1.027	2	25.123	5.684	0.233	F	0.558
11405965	1.179	4	35.866	7.528	0.236	F	0.559
91268	1.046	0	0.000	0.000	0.269	F	0.567
98994	0.806	1	30.332	6.879	0.275	F	0.568
216327	0.910	0	54.597	20.164	0.279	F	0.569
2315	1.021	1	30.332	9.573	0.288	F	0.571
10250769	1.053	2	30.332	7.765	0.289	F	0.572
173250	1.030	1	6.579	0.097	0.294	F	0.573
10090	1.058	3	67.587	20.399	0.324	F	0.580
160557	1.104	4	21.588	0.000	0.369	F	0.591
16040217	1.457	2	0.000	0.000	0.410	F	0.601
11210478	1.283	0	36.398	16.629	0.442	F	0.609
126094	0.853	3	43.844	7.123	0.501	F	0.623
227260	1.085	2	37.256	9.620	0.506	F	0.624
72154	0.844	0	47.096	14.404	0.585	F	0.642
196968	0.983	0	38.113	12.487	0.601	F	0.646
83813	1.031	2	60.132	17.208	0.656	F	0.658
2155	1.238	0	0.000	0.000	0.668	F	0.661
91683	0.922	0	41.933	12.588	0.704	F	0.669
121596244	0.895	0	17.892	2.523	0.711	F	0.671
219104	0.689	1	24.641	0.000	0.776	F	0.685
94532	1.082	1	37.256	9.955	0.802	F	0.690
2451	1.386	1	29.800	9.858	0.964	F	0.724
11339	1.196	11	66.220	0.000	0.970	F	0.725
13738	1.329	4	49.336	9.894	1.043	F	0.739
13726	0.926	0	23.202	2.988	1.047	F	0.740
11236633	1.029	1	23.202	2.053	1.055	F	0.742
91739	0.907	0	29.268	4.848	1.101	F	0.750
93379	1.456	0	29.800	12.100	1.105	F	0.751
10275	0.987	0	41.669	9.867	1.262	F	0.779
14343	1.136	0	35.488	9.098	1.294	F	0.785
121940	0.782	0	22.591	-0.670	1.299	F	0.786
2081	1.526	1	23.734	6.883	1.343	F	0.793

10262683	1.073	0	20.681	0.000	1.672	F	0.842
10358610	0.851	1	47.467	5.279	1.744	F	0.851
91741	1.130	0	29.268	2.923	1.876	F	0.867
15965	0.731	0	34.803	0.000	1.880	F	0.868
11984562	1.678	0	23.734	6.831	2.022	F	0.883
10313100	1.960	0	0.000	0.000	2.169	F	0.897
127657	1.334	0	40.869	8.391	2.170	F	0.897
211207	2.032	0	0.000	0.000	2.317	F	0.910
992	0.882	0	58.005	0.000	3.704	F	0.976

CID: compound ID; Class.: classification; DF: discriminant function; C.L.: confidence level of the classification provided by the model; GATS3c: 2D Geary Autocorrelation Index. Charge similarity at 3-bond distance; NssCH2: Atom-Centered Fragment Count Index. Number of sp3 methylene groups; PEOE_VSA6: Partial Charge Surface Area Descriptor. Surface area with moderate positive charges; VSA_EState6: E-State/Van der Waals Surface Area Hybrid Descriptor. Surface area of intermediate E-State atoms.

Table S9. Classification and probability of classification for training set compounds in Model 9 (LDA-Mordred) trained with Pubchem-WIPO data, focusing on Fungicide activity prediction.

CID	EState_VSA4	VSA_EState4	nN	DF	Class..	P.C.
**Active Group**						
7430	12.133	5.353	2	-1.958	NF	0.876
5430	22.552	4.750	3	-1.847	NF	0.863
11292824	5.563	1.654	1	-0.011	NF	0.502
32518	13.090	0.000	1	0.019	F	0.505
18771	22.674	-0.533	2	0.193	F	0.549
11159	20.013	0.000	2	0.213	F	0.554
17581	0.000	0.000	0	0.601	F	0.646
24462	0.000	0.000	0	0.601	F	0.646
30154	0.000	0.000	0	0.601	F	0.646
6327657	0.000	0.000	0	0.601	F	0.646
10788	16.866	0.000	2	0.610	F	0.649
8607	4.305	0.656	1	0.738	F	0.677
9578570	12.133	0.722	2	0.779	F	0.686
17432	11.532	0.000	2	1.282	F	0.783
25429	11.033	1.679	3	1.420	F	0.806
5455	8.641	0.000	2	1.646	F	0.839
2730	0.000	0.000	1	1.668	F	0.841
39676	4.900	-1.084	1	1.691	F	0.844
16682942	0.000	5.167	4	1.816	F	0.860
6720	0.000	-0.548	1	1.992	F	0.880
50367	10.855	0.737	3	1.999	F	0.881
66461	12.842	-0.404	3	2.423	F	0.919
3032581	0.000	0.000	2	2.735	F	0.939
15910	0.000	-0.085	2	2.785	F	0.942
91699	12.487	-1.108	3	2.883	F	0.947
86132	5.563	-0.059	3	3.136	F	0.958
3034285	0.000	1.016	3	3.201	F	0.961
86173	0.000	0.715	3	3.379	F	0.967
1810180	0.000	0.000	3	3.802	F	0.978
9257	0.000	0.000	3	3.802	F	0.978
41368	0.000	-0.358	3	4.014	F	0.982
39385	0.000	-0.553	3	4.129	F	0.984
3032792	0.000	1.097	4	4.221	F	0.986
**Inactive Group**						
7083	27.161	9.936	3	-5.493	NF	0.996
9844338	30.583	6.724	2	-5.093	NF	0.994
2955	0.000	12.092	2	-4.411	NF	0.988
120202	54.940	2.047	3	-4.330	NF	0.987
33557	0.000	13.500	3	-4.177	NF	0.985
3058751	17.696	5.830	1	-4.007	NF	0.982
5359271	30.885	2.619	1	-3.772	NF	0.977
6917740	34.079	1.827	1	-3.706	NF	0.976
16351	48.288	2.182	3	-3.572	NF	0.972

25147683	49.030	0.206	2	-3.565	NF	0.972
177335	49.383	1.812	3	-3.491	NF	0.970
657310	24.657	2.723	1	-3.048	NF	0.955
65856	30.152	1.104	1	-2.784	NF	0.942
23663941	0.000	7.267	1	-2.627	NF	0.932
9813116	0.000	9.019	2	-2.595	NF	0.930
6917865	29.433	0.809	1	-2.519	NF	0.925
8590	44.191	1.246	3	-2.503	NF	0.924
10461	23.266	0.000	0	-2.330	NF	0.911
9838802	31.371	0.065	1	-2.323	NF	0.910
5387	42.431	2.692	4	-2.068	NF	0.887
64393	44.057	0.362	3	-1.963	NF	0.876
44158	0.000	6.058	1	-1.912	NF	0.871
171450	18.228	0.000	0	-1.696	NF	0.845
21720	18.653	1.692	1	-1.682	NF	0.843
36324	11.375	6.823	3	-1.664	NF	0.840
7215	34.820	0.000	2	-1.652	NF	0.839
216236	12.966	6.464	3	-1.652	NF	0.838
66384	19.634	1.173	1	-1.499	NF	0.817
36920	17.923	1.393	1	-1.413	NF	0.804
66368	17.734	1.094	1	-1.213	NF	0.770
3036461	0.000	4.586	1	-1.043	NF	0.739
65701	25.328	2.412	3	-0.815	NF	0.692
9548842	18.718	0.000	1	-0.691	NF	0.665
68889	11.275	4.931	3	-0.533	NF	0.629
9571001	19.510	1.237	2	-0.454	NF	0.611
42510	13.090	3.800	3	-0.093	NF	0.522
3034005	17.103	1.090	2	-0.064	NF	0.515
636970	10.524	2.355	2	0.017	F	0.505
16367	0.000	8.001	4	0.140	F	0.536
82238	0.000	0.000	0	0.601	F	0.646
6371	0.000	0.000	0	0.601	F	0.646
7568320	6.545	0.000	1	0.843	F	0.699
15365	0.000	0.000	1	1.668	F	0.841
5483	0.000	0.000	1	1.668	F	0.841
61948	0.000	0.000	2	2.735	F	0.939
5479	6.197	0.000	3	3.021	F	0.954

CID: compound ID; Class.: classification; DF: discriminant function; P.C.: probability of classification; EState_VSA4: Electrotopological State (E-State) + Van der Waals Surface Area (VSA) Hybrid Descriptor. Portion of molecular Van der Waals surface area where atoms have E-State values in bin 4; VSA_EState4: absolute Van der Waals surface area (Å^2) of atoms with E-State values in bin 4; nN: Total number of nitrogen atoms in the molecule.

Table S10. Classification and probability of classification for training set compounds in Model 10 (ANN- Mordred) trained with WIPO data, focusing on Fungicide activity prediction.

CID	ZMIC2	Lipinski	n6HRing	Class.	C.L.
**Active Group**					
5634	27.470	1	0	F	0.513
7223	60.310	0	0	F	0.518
11159	60.478	0	0	F	0.519
6950	27.851	1	0	F	0.526
22321033	28.169	1	0	F	0.536
41368	28.265	1	0	F	0.539
124962	69.549	0	0	F	0.549
66461	28.841	1	0	F	0.557
1493	28.853	1	0	F	0.557
6191	28.853	1	0	F	0.557
17776	28.916	1	0	F	0.559
6451142	29.697	1	0	F	0.580
91699	30.081	1	0	F	0.590
122087	30.699	1	0	F	0.604
6112114	30.832	1	0	F	0.606
11292824	30.982	1	0	F	0.609
10935908	31.089	1	0	F	0.611
6437379	31.323	1	0	F	0.616
6422843	31.344	1	0	F	0.616
17581	32.166	1	0	F	0.629
8606	46.703	1	0	F	0.630
3032581	45.752	1	0	F	0.632
15910	56.881	1	0	F	0.633
25202562	44.700	1	0	F	0.634
3037	32.874	1	0	F	0.637
65258	43.250	1	0	F	0.638
5455	43.227	1	0	F	0.638
3032792	43.095	1	0	F	0.639
16682924	43.023	1	0	F	0.639
17110	42.896	1	0	F	0.639
56840815	41.181	1	0	F	0.645
1730	41.000	1	0	F	0.645
23663539	64.386	1	0	F	0.646
16682936	33.849	1	0	F	0.646
6720	64.816	1	0	F	0.646
10598	40.253	1	0	F	0.648
5460680	39.595	1	0	F	0.649
45380430	34.653	1	0	F	0.650
16682983	35.255	1	0	F	0.653
12318	35.345	1	0	F	0.653
11486133	37.927	1	0	F	0.653
72980153	37.927	1	0	F	0.653
3647458	37.795	1	0	F	0.654

16684215	37.537	1	0	F	0.654
213016	36.195	1	0	F	0.654
9578570	36.195	1	0	F	0.654
24462	36.293	1	0	F	0.654
16682942	36.769	1	0	F	0.654
14309	36.688	1	0	F	0.654
11953884	66.107	0	1	F	0.680
2730	39.834	1	1	F	0.683
18771	45.340	1	1	F	0.741
**Inactive Group**					
15402	27.32	1	0	F	0.508
119569	27.719	1	0	F	0.522
13676	28.416	1	0	F	0.544
12620	76.665	0	0	F	0.551
72300	28.747	1	0	F	0.554
198707	30.624	1	0	F	0.602
82178	31.754	1	0	F	0.623
2789	32.147	1	0	F	0.628
10903489	47.639	1	0	F	0.629
88842	55.183	1	0	F	0.631
121957	43.015	1	0	F	0.639
11001318	34.003	1	0	F	0.647
11425584	34.093	1	0	F	0.647
15532	34.25	1	0	F	0.648
101815861	37.601	1	0	F	0.654
123805	56.643	0	0	NF	0.504
127151	33.814	1	1	NF	0.505
216249	26.883	1	0	NF	0.507
50367	33.733	1	1	NF	0.509
101209015	25.891	1	0	NF	0.541
11048796	36.379	1	2	NF	0.543
11033	25.721	1	0	NF	0.547
2078	25.696	1	0	NF	0.548
23690429	25	1	0	NF	0.571
72064	32.095	1	1	NF	0.578
86132	24.719	1	0	NF	0.579
28780	24.556	1	0	NF	0.584
2075	24.468	1	0	NF	0.587
1455	45.912	0	0	NF	0.593
9257	13.5	1	0	NF	0.597
5430	24.106	1	0	NF	0.597
77139	24.083	1	0	NF	0.598
222284	42.62	0	0	NF	0.618
93379	30.87	1	1	NF	0.627
13985	40.326	0	0	NF	0.631
110992	22.595	1	0	NF	0.633
33112	22.542	1	0	NF	0.634

5366415	22.491	1	0	NF	0.635
1727	14.756	1	1	NF	0.635
13643	22.409	1	0	NF	0.637
14454445	38.693	0	0	NF	0.639
10007	22.162	1	0	NF	0.641
11316914	40.95	0	1	NF	0.644
999	21.914	1	0	NF	0.645
12130	21.903	1	0	NF	0.645
2557	21.877	1	0	NF	0.646
30154	21.738	1	0	NF	0.648
10419733	21.678	1	0	NF	0.649
59649244	32.78	0	0	NF	0.65
89124	16.608	1	0	NF	0.65
151230	21.375	1	0	NF	0.653
10783	21.292	1	0	NF	0.654
10313100	16.999	1	0	NF	0.654
1864	21.253	1	0	NF	0.654
194233	17.108	1	0	NF	0.655
76962	17.207	1	0	NF	0.656
10047015	32.826	1	2	NF	0.659
1893	20.8	1	0	NF	0.659
1646	20.739	1	0	NF	0.659
112056	20.41	1	0	NF	0.662
159247	20.372	1	0	NF	0.662
73801	29.835	1	1	NF	0.662
232487	16.128	1	1	NF	0.663
227	20.034	1	0	NF	0.664
1140	18.645	1	0	NF	0.665
14777	18.806	1	0	NF	0.665
10176082	19.358	1	0	NF	0.666
996	19.124	1	0	NF	0.666
92411	29.652	1	1	NF	0.668
114681	17.085	1	1	NF	0.681
104781	35.692	1	3	NF	0.691
133128	32.797	0	1	NF	0.692
1018	18.018	1	1	NF	0.696
100516	30.262	1	2	NF	0.698
1050	19.209	1	1	NF	0.713
219095	30.438	0	1	NF	0.717
115223	29.677	1	3	NF	0.718
1720	26.94	1	1	NF	0.726
123983	26.56	1	1	NF	0.731
78165	26.559	1	1	NF	0.731
10788	21.51	1	1	NF	0.736
10016922	22.485	1	1	NF	0.742
168045	22.528	1	1	NF	0.742
15706387	24.512	1	1	NF	0.744

CID: compound classification; Class.: classification; C.L.: confidence level of the classification provided by the model; ZMIC2: Zagreb Index (modified). Topological index emphasizing heteroatoms/bonds.; Lipinski: Drug-likeness rule. Binary flag for Rule of Five compliance; n6HRing: Heterocycle count. Number of 6-membered rings with heteroatoms.

Table S11. Classification and probability of classification for training set compounds in Model 11 (ANN- Mordred) trained with FRAC-WIPO data, focusing on Fungicide activity prediction.

CID	GATS3c	NssCH2	PEOE_VSA6	VSA_EState6	Class.	C.L.
**Active Group**						
penconazole	0.740	3	42.613	5.678	F	0.518
flutriafol	0.944	1	30.332	11.247	F	0.518
picoxystrobin	0.931	1	30.332	10.135	F	0.554
hymexazole	1.170	0	0.000	1.389	F	0.560
triflumizole	1.046	3	18.525	3.467	F	0.569
triticonazole	0.895	3	43.657	7.669	F	0.582
iodocarb	1.120	4	19.265	0.000	F	0.598
ametoctradin	0.926	8	45.952	0.000	F	0.601
dichlofluanid	0.993	0	41.401	7.965	F	0.611
amobam	0.841	2	24.436	0.000	F	0.614
mandestrobin	1.004	1	36.398	13.792	F	0.619
dimethirimol	1.079	3	13.345	0.000	F	0.621
diethofencarb	1.577	2	0.000	5.227	F	0.627
edifenphos	1.335	1	36.398	19.419	F	0.629
diclomezine	0.747	0	23.202	6.562	F	0.632
tolylfluanid	1.015	0	40.898	6.499	F	0.640
tricyclazole	0.753	0	23.470	6.289	F	0.659
benthiavalicarb	0.910	0	13.847	3.068	F	0.661
imibenconazole	0.929	2	46.936	12.996	F	0.665
cyazofamid	1.231	0	41.430	8.896	F	0.669
pyrimorph	1.089	4	56.638	12.024	F	0.674
tebuconazole	1.012	3	44.505	7.761	F	0.678
fluxapyroxad	1.033	0	18.199	7.491	F	0.679
mefentrifluconazole	1.117	1	17.667	9.659	F	0.693
binapacryl	0.992	1	19.421	2.017	F	0.702
thiazole	1.393	0	0.000	0.000	F	0.706
pyrifenox	0.769	1	40.490	9.147	F	0.708
pyrisoxazole	1.281	1	29.800	11.909	F	0.711
epoxiconazole	0.986	1	41.933	13.932	F	0.721
metrafenone	1.075	0	15.930	5.366	F	0.727
simeconazole	0.997	2	31.774	6.724	F	0.732
cymoxanil	1.487	1	5.156	0.799	F	0.736
azoxystrobin	1.357	0	30.332	17.197	F	0.744
thiophanate methyl	1.348	0	12.133	6.915	F	0.753
dimoxystrobin	1.059	1	41.554	13.568	F	0.759
pyridachlometyl	1.033	0	47.999	12.906	F	0.761
fthalide	0.803	1	46.404	0.000	F	0.762
phenamacril	1.316	1	30.332	10.608	F	0.770
pyraoxystrobin	1.055	1	47.999	16.737	F	0.777
pyrametostrobin	1.073	1	48.531	17.286	F	0.779
tolfenpyrad	1.251	2	48.354	15.496	F	0.780
carbendazim	1.446	0	12.133	7.507	F	0.782
fenfuram	1.454	0	18.199	10.989	F	0.792

metominostrobin	1.257	0	35.488	16.496	F	0.794
kresoxim-methyl	1.024	1	47.620	15.114	F	0.795
trifloxystrobin	0.875	1	46.710	11.545	F	0.801
fluopyram	0.937	2	23.734	4.958	F	0.803
isopyrazam	0.923	2	25.980	5.982	F	0.808
acibenzolar-s-methyl	1.062	0	22.316	5.490	F	0.816
bupirimate	1.602	4	13.345	0.000	F	0.823
thiophanate	1.496	2	12.133	6.966	F	0.824
spiroxamine	1.531	9	34.619	0.000	F	0.826
cyprodinil	1.612	2	18.199	12.122	F	0.834
fluquinconazole	1.271	0	23.202	8.483	F	0.838
benalaxyl-m	1.109	1	48.531	14.652	F	0.838
pyributicarb	1.280	0	38.970	13.470	F	0.839
mepronil	1.770	0	24.265	14.948	F	0.853
oxpoconazole	1.746	4	23.734	7.788	F	0.858
fluazinam	0.923	0	23.202	0.301	F	0.859
probenazole	1.345	1	24.788	6.578	F	0.860
carboxin	1.496	2	18.199	9.422	F	0.860
propiconazole	1.231	4	42.613	5.322	F	0.866
fluoroimide	1.198	0	23.202	4.863	F	0.870
ferimzone	1.493	0	24.265	10.097	F	0.876
azaconazole	1.182	3	29.268	5.246	F	0.876
silthiofam	1.159	1	25.717	0.000	F	0.878
chinomethionat	1.112	0	28.740	5.917	F	0.880
pyrimethanil	1.797	0	18.199	11.863	F	0.882
benomyl	1.422	3	25.477	6.833	F	0.882
procymidone	1.524	1	23.202	4.739	F	0.884
prothiocarb	0.352	4	18.686	0.000	F	0.889
tebufloquin	1.307	0	20.771	3.359	F	0.891
quinoxyfen	1.658	0	23.202	10.793	F	0.894
etridiazole	1.103	1	34.803	0.000	F	0.899
penflufen	1.363	1	38.970	7.689	F	0.901
validamycin	0.744	4	6.076	-1.935	F	0.901
triclopyricarb	1.022	1	53.002	8.420	F	0.910
tiadinil	1.525	0	22.156	5.386	F	0.912
triadimefon	1.277	0	32.372	6.831	F	0.916
pydiflumetofen	1.232	1	34.803	2.521	F	0.917
triforine	0.876	4	69.606	0.000	F	0.920
oxycarboxin	1.669	2	18.199	8.681	F	0.924
captafol	1.014	2	35.354	0.000	F	0.924
fenarimol	0.943	0	53.534	14.134	F	0.924
furametpyr	1.662	0	23.734	5.851	F	0.929
mepanipyrim_	1.800	0	24.120	11.701	F	0.930
dodin	0.925	11	64.711	0.000	F	0.934
imazalil	0.998	2	35.344	5.410	F	0.934
fosetyl-al	2.202	3	0.000	0.000	F	0.942
etaconazole	1.250	3	36.192	5.297	F	0.946

isoprothiolane	2.077	2	0.000	0.000	F	0.947
propamocarb	1.141	5	6.924	0.000	F	0.947
tecnazene	1.082	0	46.404	1.251	F	0.955
dodemorph	1.578	13	57.787	0.822	F	0.958
natamycin	1.127	5	48.608	-1.077	F	0.959
dimethachlone	1.552	2	23.202	4.654	F	0.961
dichlobentiazox	1.264	1	35.335	6.477	F	0.964
zoxamide	1.294	2	30.126	3.054	F	0.967
mattifine	0.732	2	84.948	25.566	F	0.968
tridemorph	1.528	14	71.132	0.000	F	0.984
carpropamid	1.479	1	37.581	7.304	F	0.984
captan	1.019	2	46.955	0.000	F	0.985
iprobenfos	1.847	1	30.332	9.897	F	0.997
terbinafine	1.005	2	60.381	15.073	F	1.000
zineb	0.166	2	8.641	0.000	F	1.000
bitertanol	1.148	0	63.236	17.981	F	1.000
diclocymet	1.528	0	50.039	6.892	F	1.000
febram	0.193	0	4.321	0.000	F	1.000
fenazaquin	1.221	2	57.169	16.673	F	1.000
iprovalicarb	1.368	0	43.676	7.240	F	1.000
picarbutrazox	1.634	1	41.554	14.654	F	1.000
propineb	0.176	1	8.641	0.168	F	1.000
quintozene	1.066	0	58.005	0.000	F	1.000
streptomycin	0.983	1	0.000	-3.953	F	1.000
tecloftalam	0.923	0	75.672	4.538	F	1.000
thiram	0.362	0	24.436	0.000	F	1.000
ziram	0.066	0	4.321	0.000	F	1.000
metalaxyl	0.932	1	18.199	5.011	NF	0.502
metalaxyl_m	0.932	1	18.199	5.011	NF	0.502
cyclobutrifluram	0.938	2	29.268	7.222	NF	0.508
fenhexamid	0.854	5	49.388	2.987	NF	0.512
octhilinone	0.911	7	50.560	1.641	NF	0.543
proquinazid	1.530	4	13.847	6.125	NF	0.557
ethirimol	1.198	4	13.345	0.000	NF	0.564
difenoconazole	1.424	2	23.202	12.535	NF	0.581
ofurace	1.027	3	18.199	5.185	NF	0.590
pencycuron	1.391	5	54.774	17.617	NF	0.597
fenpropidin	1.307	7	58.381	9.270	NF	0.625
thifluzamide	0.968	0	0.000	1.826	NF	0.647
thiabendazole	0.877	0	12.133	7.971	NF	0.655
cinnamaldehyde	0.687	0	36.408	9.702	NF	0.663
fenpiclonil	0.581	0	35.335	7.437	NF	0.673
blasticidin_s	1.094	3	6.076	-0.070	NF	0.675
amisulbrom	1.043	0	0.000	3.717	NF	0.677
methasulfocarb	1.169	0	0.000	6.208	NF	0.690
pyraclostrobin	0.983	1	29.800	16.283	NF	0.713
sedaxane	1.054	3	18.199	7.681	NF	0.732

pyraziflumid	0.877	0	24.265	9.141	NF	0.777
oxathiapiprolin	1.075	6	11.222	4.564	NF	0.829
myclobutanil	0.779	4	43.499	10.009	NF	0.850
tavaborole	0.885	1	6.066	4.241	NF	0.870
flutianil	0.826	2	23.895	11.431	NF	0.937
oxolinic_acid	0.963	2	0.000	3.243	NF	0.983
**Inactive Group**						
91268	1.046	0	0.000	0.000	F	0.509
11465618	1.517	4	47.999	17.889	F	0.526
93379	1.456	0	29.800	12.100	F	0.555
14343	1.136	0	35.488	9.098	F	0.562
10250769	1.053	2	30.332	7.765	F	0.566
10275	0.987	0	41.669	9.867	F	0.572
1858	1.074	2	11.601	3.759	F	0.572
126094	0.853	3	43.844	7.123	F	0.578
10358610	0.851	1	47.467	5.279	F	0.600
219069	0.766	1	41.933	13.141	F	0.614
92411	1.217	4	25.448	5.065	F	0.631
2315	1.021	1	30.332	9.573	F	0.641
160557	1.104	4	21.588	0.000	F	0.668
227260	1.085	2	37.256	9.620	F	0.668
127657	1.334	0	40.869	8.391	F	0.698
219104	0.689	1	24.641	0.000	F	0.699
2712	1.032	1	41.933	15.107	F	0.710
123920	0.834	3	29.268	3.929	F	0.714
13738	1.329	4	49.336	9.894	F	0.730
72154	0.844	0	47.096	14.404	F	0.751
121596244	0.895	0	17.892	2.523	F	0.752
11236633	1.029	1	23.202	2.053	F	0.786
94532	1.082	1	37.256	9.955	F	0.788
2451	1.386	1	29.800	9.858	F	0.809
11210478	1.283	0	36.398	16.629	F	0.811
13726	0.926	0	23.202	2.988	F	0.827
91739	0.907	0	29.268	4.848	F	0.853
91452	1.027	2	25.123	5.684	F	0.854
11984562	1.678	0	23.734	6.831	F	0.925
150315	0.726	1	24.265	5.355	NF	0.512
194680	0.926	3	23.202	5.460	NF	0.513
173250	1.030	1	6.579	0.097	NF	0.534
108089	1.215	3	17.696	7.472	NF	0.536
13770	0.771	4	66.256	17.615	NF	0.548
172975	1.418	3	12.133	7.774	NF	0.549
122067	1.076	0	0.000	1.948	NF	0.551
6	0.725	0	11.601	3.051	NF	0.555
216249	1.175	3	11.601	3.307	NF	0.564
1533	1.159	0	0.000	3.746	NF	0.568
134898	1.186	5	0.000	2.404	NF	0.574

15907	1.096	2	11.601	7.012	NF	0.585
16040217	1.457	2	0.000	0.000	NF	0.600
163797	1.398	2	0.000	5.645	NF	0.623
11405965	1.179	4	35.866	7.528	NF	0.632
10199199	0.964	4	44.002	9.059	NF	0.646
129228	0.937	1	11.280	3.555	NF	0.649
208947	1.145	3	18.199	8.197	NF	0.657
219095	1.271	1	17.667	12.887	NF	0.668
100413	0.792	1	17.667	5.446	NF	0.673
10130337	0.899	2	30.332	9.571	NF	0.677
10178705	0.981	7	18.022	1.132	NF	0.681
15459	1.156	2	6.066	6.026	NF	0.686
11683556	1.231	0	0.000	7.291	NF	0.689
460	0.768	0	12.133	6.838	NF	0.697
2040	1.168	1	18.199	12.165	NF	0.707
134391533	1.101	1	11.223	10.097	NF	0.709
10848	1.576	5	6.924	0.000	NF	0.716
194233	1.320	2	0.000	0.000	NF	0.728
187238	1.213	2	6.066	7.758	NF	0.735
11339	1.196	11	66.220	0.000	NF	0.736
187888	0.824	1	30.332	8.809	NF	0.742
15706387	1.156	4	36.753	10.217	NF	0.758
157839	0.777	2	18.199	7.782	NF	0.760
12056759	0.714	2	19.056	3.480	NF	0.768
10013505	1.219	5	6.924	0.000	NF	0.769
98994	0.806	1	30.332	6.879	NF	0.775
182137	1.007	4	36.221	7.616	NF	0.775
10419733	1.135	4	0.000	0.000	NF	0.793
108137	1.117	2	30.332	16.009	NF	0.796
92266	0.873	0	0.000	3.510	NF	0.812
119574	1.006	6	11.601	3.409	NF	0.812
11622909	1.279	4	30.332	10.087	NF	0.830
216468	0.813	0	23.734	9.627	NF	0.831
104901	0.891	0	24.778	11.899	NF	0.838
11316914	1.132	6	53.356	12.683	NF	0.845
92357	0.898	1	0.000	0.000	NF	0.846
150949	0.788	1	11.601	5.375	NF	0.848
102669	1.279	6	44.179	9.860	NF	0.856
127151	1.245	4	42.465	17.991	NF	0.862
11494412	0.976	3	36.398	14.227	NF	0.862
2790	0.898	4	35.496	7.643	NF	0.868
192706	1.144	2	25.123	15.260	NF	0.872
98895	0.935	0	0.000	6.286	NF	0.874
185236	1.101	5	0.000	3.337	NF	0.876
172309	1.163	3	6.066	4.660	NF	0.876
10783	1.118	2	0.000	0.000	NF	0.878
1018	0.602	0	6.066	4.755	NF	0.892

214356	1.009	2	6.066	2.600	NF	0.898
92965	1.128	6	43.173	7.927	NF	0.899
11105	1.078	0	0.000	9.962	NF	0.908
10718	0.966	0	18.199	12.429	NF	0.911
119198	0.876	2	6.924	5.743	NF	0.913
2200	1.100	4	48.531	21.077	NF	0.915
78569	0.744	1	12.133	7.693	NF	0.929
160883	1.123	6	43.676	10.285	NF	0.946
14987	0.732	3	18.209	6.922	NF	0.947
13320	0.492	2	42.465	17.795	NF	0.949
192737	0.948	2	0.000	0.000	NF	0.950
159598	1.174	3	0.000	3.513	NF	0.950
14454445	0.777	0	18.209	12.211	NF	0.956
72413	0.949	3	0.000	0.000	NF	0.959
16046068	0.937	3	30.332	14.067	NF	0.961
211207	2.032	0	0.000	0.000	NF	0.966
1883	0.580	1	0.000	-0.539	NF	0.967
72103	0.644	3	13.345	3.485	NF	0.968
92425	0.995	6	5.920	2.367	NF	0.969
11326715	1.100	1	0.000	11.963	NF	0.973
2583	1.256	4	6.066	5.594	NF	0.975
10770	1.180	6	37.462	9.757	NF	0.976
1684	0.811	3	10.313	5.228	NF	0.976
100580	0.799	6	12.990	3.768	NF	0.978
2683	0.795	15	90.040	6.315	NF	0.981
11427553	0.972	4	36.408	15.790	NF	0.981
159500	1.175	5	18.209	7.599	NF	0.982
122737	0.803	3	12.133	6.928	NF	0.983
14683796	1.034	6	6.066	3.936	NF	0.983
130305	0.911	2	0.000	2.465	NF	0.984
151178	0.351	0	0.000	2.207	NF	0.984
2169	0.891	3	24.265	12.058	NF	0.985
159947	0.138	15	97.821	0.000	NF	0.987
117947705	0.919	6	47.999	13.588	NF	0.988
80084	0.343	4	60.664	20.981	NF	0.988
72076	1.074	6	49.240	18.297	NF	0.993
192251	0.299	11	76.489	9.669	NF	0.993
2267	1.128	6	41.933	15.831	NF	0.996
164509	0.825	2	12.133	11.373	NF	0.996
101744	0.875	4	24.275	10.270	NF	0.996
152035	1.007	5	12.133	4.168	NF	0.997
122536283	0.875	3	6.066	7.784	NF	0.997
10939	0.206	3	0.000	0.000	NF	0.997
14129	0.679	1	12.133	10.465	NF	0.997
119114	1.221	5	24.265	12.510	NF	0.998
127873	0.699	2	0.000	3.295	NF	0.998
160154	0.609	6	25.123	8.625	NF	0.999

177358	0.041	0	6.066	5.856	NF	0.999
158789	0.841	7	31.395	6.439	NF	0.999
2369	1.223	7	25.980	8.414	NF	0.999
2562	1.175	10	57.021	10.083	NF	0.999
73341	0.422	3	12.990	6.105	NF	0.999
129791	0.970	3	12.133	12.794	NF	0.999
194066	0.539	6	12.990	3.858	NF	1.000
93154	1.371	7	36.398	18.794	NF	1.000
15387	1.050	7	6.924	5.832	NF	1.000
15938	0.092	0	0.000	8.481	NF	1.000
12717441	1.517	6	0.000	7.825	NF	1.000
91614	0.307	2	5.156	8.756	NF	1.000
10262683	1.073	0	20.681	0.000	NF	1.000
11623906	0.843	7	0.000	1.673	NF	1.000
119593	0.739	7	0.000	2.067	NF	1.000
11243969	0.911	8	0.000	1.722	NF	1.000
101673418	0.213	9	0.000	0.000	NF	1.000
10445549	0.680	1	0.000	-1.084	NF	1.000
121940	0.782	0	22.591	-0.670	NF	1.000
12302171	0.829	5	0.000	-3.291	NF	1.000
15965	0.731	0	34.803	0.000	NF	1.000
216327	0.910	0	54.597	20.164	NF	1.000
82143	1.074	0	36.398	18.908	NF	1.000
992	0.882	0	58.005	0.000	NF	1.000
134819291	0.144	9	0.000	0.000	NF	1.000
221227	0.867	0	36.398	21.419	NF	1.000
2391	1.292	0	30.332	20.485	NF	1.000

CID: compound ID; Class.: classification; C.L.: confidence level of the classification provided by the model. GATS3c: 2D Geary Autocorrelation Index. Charge similarity at 3-bond distance; NssCH2: Atom-Centered Fragment Count Index. Number of sp3 methylene groups; PEOE_VSA6: Partial Charge Surface Area Descriptor. Surface area with moderate positive charges; VSA_EState6: E-State/Van der Waals Surface Area Hybrid Descriptor. Surface area of intermediate E-State atoms.

Table S12. Classification and probability of classification for training set compounds in Model 12 (ANN- Mordred) trained with Pubchem-WIPO data, focusing on Fungicide activity prediction.

CID	EState_VSA4	nN	VSA_EState4	Class.	C.L.
**Active Group**					
3032581	0.000	2	0.000	F	0.584
24462	0.000	0	0.000	F	0.624
30154	0.000	0	0.000	F	0.624
6327657	0.000	0	0.000	F	0.624
15910	0.000	2	-0.085	F	0.640
86132	5.563	3	-0.059	F	0.642
1810180	0.000	3	0.000	F	0.660
9257	0.000	3	0.000	F	0.660
5455	8.641	2	0.000	F	0.667
91699	12.487	3	-1.108	F	0.673
66461	12.842	3	-0.404	F	0.682
3034285	0.000	3	1.016	F	0.688
3032792	0.000	4	1.097	F	0.698
17432	11.532	2	0.000	F	0.712
6720	0.000	1	-0.548	F	0.721
32518	13.090	1	0.000	F	0.722
11159	20.013	2	0.000	F	0.729
39676	4.900	1	-1.084	F	0.730
18771	22.674	2	-0.533	F	0.733
10788	16.866	2	0.000	F	0.735
50367	10.855	3	0.737	F	0.735
9578570	12.133	2	0.722	F	0.736
16682942	0.000	4	5.167	F	0.736
39385	0.000	3	-0.553	F	0.762
2730	0.000	1	0.000	NF	0.548
7430	12.133	2	5.353	NF	0.719
**Inactive Group**					
61948	0.000	2	0.000	F	0.584
82238	0.000	0	0.000	F	0.624
5479	6.197	3	0.000	F	0.650
15365	0.000	1	0.000	NF	0.548
5483	0.000	1	0.000	NF	0.548
25147683	49.030	2	0.206	NF	0.712
6917740	34.079	1	1.827	NF	0.716
65856	30.152	1	1.104	NF	0.716
3034005	17.103	2	1.090	NF	0.717
5359271	30.885	1	2.619	NF	0.718
636970	10.524	2	2.355	NF	0.718
7215	34.820	2	0.000	NF	0.718
657310	24.657	1	2.723	NF	0.718
120202	54.940	3	2.047	NF	0.718
64393	44.057	3	0.362	NF	0.719
177335	49.383	3	1.812	NF	0.719

8590	44.191	3	1.246	NF	0.719
5387	42.431	4	2.692	NF	0.719
7083	27.161	3	9.936	NF	0.719
9844338	30.583	2	6.724	NF	0.719
33557	0.000	3	13.500	NF	0.719
216236	12.966	3	6.464	NF	0.719
36324	11.375	3	6.823	NF	0.719
68889	11.275	3	4.931	NF	0.719
65701	25.328	3	2.412	NF	0.719
42510	13.090	3	3.800	NF	0.719
9813116	0.000	2	9.019	NF	0.719
9571001	19.510	2	1.237	NF	0.719
3058751	17.696	1	5.830	NF	0.719
66384	19.634	1	1.173	NF	0.719
21720	18.653	1	1.692	NF	0.719
36920	17.923	1	1.393	NF	0.719
66368	17.734	1	1.094	NF	0.719
23663941	0.000	1	7.267	NF	0.719
44158	0.000	1	6.058	NF	0.720
3036461	0.000	1	4.586	NF	0.721
9548842	18.718	1	0.000	NF	0.727
171450	18.228	0	0.000	NF	0.731

CID: compound ID; Class.: classification; C.L.: confidence level of the classification provided by the model; EState_VSA4: Electrotopological State (E-State) + Van der Waals Surface Area (VSA) Hybrid Descriptor. Portion of molecular Van der Waals surface area where atoms have E-State values in bin 4; VSA_EState4: absolute Van der Waals surface area ($Å^2$) of atoms with E-State values in bin 4; nN: Total number of nitrogen atoms in the molecule.

Table S13. Classification and probability of classification for internal test set compounds in Model 4 (ANN-AlvaDesc) trained with WIPO data, focusing on Fungicide activity prediction.

CID	nR06	SpMax8_Bh(m)	P_VSA_charge_6	CATS2D_01_AA	B01[C-X]	Class.	C.L.
**Active Group**							
28780	1	2.660	30.312	0	0	F	0.500
213032	3	2.693	75.810	0	0	F	0.508
1493	1	1.331	0.000	4	0	F	0.523
4921319	1	1.331	0.000	4	0	F	0.523
39385	1	2.499	65.624	1	0	F	0.558
6112114	2	2.689	85.734	1	0	F	0.564
6422843	2	2.749	67.577	2	0	F	0.572
66461	1	2.382	101.668	1	0	F	0.596
8606	1	2.561	136.398	0	0	F	0.614
9578570	2	2.838	105.133	2	0	F	0.618
11953884	1	3.541	131.365	0	0	F	0.646
1730	1	1.039	0.000	0	1	F	0.779
16684215	1	1.292	0.000	2	1	F	0.834
90545	1	1.331	0.000	2	1	F	0.835
16682924	1	1.039	0.000	3	1	F	0.854
7156993	0	0.826	0.000	0	0	NF	0.594
24462	0	0.000	0.000	0	0	NF	0.631
**Inactive Group**							
13985	2	3.151	74.519	0	0	F	0.546
110992	1	1.808	48.625	0	0	NF	0.518
2708	1	2.498	18.952	0	0	NF	0.518
123983	2	2.687	28.429	0	0	NF	0.520
14454445	3	2.916	28.429	0	0	NF	0.526
10047015	3	2.812	9.476	1	0	NF	0.528
92189	1	0.859	47.381	1	0	NF	0.533
115223	4	2.674	28.390	0	0	NF	0.551
1456	2	1.087	38.352	1	0	NF	0.557
10016922	4	2.581	9.476	0	0	NF	0.571
97663	0	1.192	0.000	0	0	NF	0.577
78165	1	1.596	0.000	0	0	NF	0.579
996	1	0.000	28.429	0	0	NF	0.622
10176082	0	0.000	0.000	0	0	NF	0.631
14777	0	0.000	0.000	0	0	NF	0.631
1018	1	0.155	9.476	0	0	NF	0.635

CID: compound ID; Class.: classification; C.L.: confidence level of the classification provided by the model; nR06: Topological descriptor (Count descriptor). Number of 6-membered rings in the molecule; SpMax8_Bh(m): The largest eigenvalue of a modified Burden matrix (B_h(m)) weighted by atomic masses (m); P_VSA_charge_6: The portion of the molecular Van der Waals surface area where the partial charge falls within a specific bin (bin 6); CATS2D_01_AA: A correlation vector descriptor that measures the occurrence of specific atom pairs (A-A) at a given topological distance (lag 01); B01[C-X]: Binary fingerprint. Presence of a carbon bonded to a heteroatom (N, O, S, etc.).

Table S14. Classification and probability of classification for internal test set compounds in Model 5 (ANN-AlvaDesc) trained with FRAC-WIPO data, focusing on Fungicide activity prediction.

CID	nStruct.	P_VSA_m_4	SsssN	NsCH3	CATS2D_04_DA	SHED_DL	Class.	C.L.
**Active Group**								
fluoxastrobin	1	39.149	0	1	0	0	1	0.502
pyridachlometyl	1	39.149	0	1	0	0	1	0.502
octhilinone	1	31.981	1.833	1	0	0	1	0.503
metconazole	1	39.149	0	2	1	5.084	1	0.504
phenamacril	1	0	0	1	3	4.757	1	0.508
epoxiconazole	1	39.149	0	0	0	0	1	0.509
benodanil	1	0	0	0	0	3.649	1	0.51
quinofumelin	1	0	0	2	0	0	1	0.526
cyclobutrifluram	1	0	0	0	0	2.828	1	0.539
bitertanol	1	0	0	0	0	2	1	0.553
tavaborole	1	0	0	0	0	2	1	0.553
cymoxanil	1	0	0	2	3	2	1	0.555
pyrazophos	1	0	0	0	0	0	1	0.559
cyprodinil	1	0	0	1	0	3.789	1	0.569
chinomethionat	1	64.439	0	1	0	0	1	0.612
mepronil	1	0	0	3	1	4.594	1	0.618
thifluzamide	1	31.278	0	1	1	1.569	1	0.635
penflufen	1	0	0	5	2	4.594	1	0.658
quinoxyfen	1	78.297	0	0	0	0	1	0.658
tebuconazole	1	39.149	0	3	1	5.345	1	0.659
pyraoxystrobin	1	39.149	0	3	0	0	1	0.672
triadimefon	1	39.149	0	3	0	0	1	0.672
fenazaquin	1	0	0	3	0	0	1	0.685
kresoxim-methyl	1	0	0	3	0	0	1	0.685
cyazofamid	1	44.718	1.004	3	0	0	1	0.695
furalaxyl	1	0	1.426	4	0	0	1	0.715
mandestrobin	1	0	0	4	0	6.586	1	0.719
pyrazophos	1	52.819	0	4	0	0	1	0.725
polyoxin	1	0	0.607	0	7	3.789	1	0.726
isofetamid	1	31.278	0	6	0	4.826	1	0.727
silthiofam	1	31.278	0	5	0	3.864	1	0.728
triforine	1	234.892	3.572	0	0	2	1	0.728
imibenconazole	1	149.665	0	0	0	0	1	0.73
prochloraz	1	117.446	1.68	1	0	0	1	0.73
pyriofenone	1	39.149	0	6	0	0	1	0.731
zineb	2	182.598	0	0	0	0	1	0.731
fludioxonil	1	0	0	0	1	4.762	2	0.533
fluopyram	1	39.149	0	0	1	4.745	2	0.547
pencycuron	1	39.149	1.962	0	0	5.427	2	0.731
**Inactive Group**								
92266	1	32.22	0	0	1	3.789	1	0.51
2391	1	0	0	2	0	0	1	0.526

100580	1	0	0	2	0	0	1	0.526
15939	1	0	0	2	0	0	1	0.526
82143	1	0	0	1	0	0	1	0.545
1533	1	0	0	1	0	2.828	1	0.588
76962	1	0	0	3	0	1.755	1	0.639
10783	1	70.426	0	1	0	0	1	0.639
15938	3	105.003	0	2	0	0	1	0.707
134391533	1	0	0	4	0	8.151	1	0.724
187888	1	0	1.065	1	0	0	2	0.501
216327	1	0	0	0	0	3.953	2	0.506
150315	1	0	0	0	0	4.409	2	0.535
15907	1	39.149	0	0	0	2.872	2	0.564
10124	1	37.324	0	0	0	3.92	2	0.583
182137	1	39.149	0	1	0	3.596	2	0.584
11683556	1	0	0	2	0	5.299	2	0.594
164867	1	44.956	0	0	0	4.353	2	0.602
98994	1	32.22	0	2	2	9.829	2	0.627
12717441	1	0	2.32	1	0	0	2	0.651
208947	1	0	0	2	0	6.928	2	0.653
13268	1	39.149	1.511	1	1	2.828	2	0.667
72413	1	0	1.057	0	2	4	2	0.68
94532	1	0	2.137	3	0	3.747	2	0.707
15851	2	83.617	0	1	0	3.596	2	0.71
185236	1	0	3.803	1	2	6.107	2	0.711
2267	1	39.149	4.091	1	0	0	2	0.722
2315	1	11.615	0	0	2	7.941	2	0.723
72103	1	0	0	1	3	8.818	2	0.725
119198	1	0	4.735	3	0	3.864	2	0.726
216239	1	39.149	0	1	0	8.837	2	0.726
1930	1	0	0	1	0	17	2	0.731
80084	2	0	0	3	0	0	2	0.731
91452	2	44.468	0	2	0	4.586	2	0.731
119593	1	0	0	0	1	11.863	2	0.731
161240	1	0	0	0	0	11.283	2	0.731
10939	2	0	0	3	0	0	2	0.731
11105	2	44.468	2.05	3	1	4.711	2	0.731
13320	2	52.501	0	2	0	10.961	2	0.731
10178705	1	39.149	3.574	0	2	8.094	2	0.731
11291932	1	0	1.69	0	0	10.429	2	0.731
12056759	2	5.807	1.21	3	0	0	2	0.731

CID: compound ID; nStruct.: number of structures; Class.: classification; C.L.: confidence level of the classification provided by the model; P_VSA_m_4: Mass-weighted VSA. VdW surface area where atomic mass falls in bin 4; SsssN: E-State descriptor. E-State sum for quaternary nitrogens (N with 4 single bonds); NsCH3: Atom-centered fragment. Count of methyl groups (–CH_3) attached to sulfur (S); CATS2D_04_DA: CATS2D correlation vector. Donor-acceptor atom pairs separated by 4 bonds; SHED_DL: Shannon entropy descriptor. Entropy of drug-like atomic property distributions.

Table S15. Classification and probability of classification for internal test set compounds in Model 6 (ANN-AlvaDesc) trained with Pubchem-WIPO data, focusing on Fungicide activity prediction.

CID	MATS2p	SaasC	SsCl	NaaN	Class.	C.L.
**Active Group**						
5430	-0.290	1.749	0.000	2	F	0.547
3032581	0.103	0.000	0.000	0	F	0.593
7430	0.092	0.173	11.099	0	F	0.721
17581	0.495	2.058	11.633	0	F	0.730
6720	0.194	-1.669	27.934	0	F	0.730
9257	0.076	0.000	0.000	2	F	0.730
33557	0.333	0.000	0.000	0	F	0.730
**Inactive Group**						
8590	-0.031	2.792	0.000	2	NF	0.513
6917865	-0.004	0.809	0.000	0	NF	0.565
16351	0.218	5.358	6.278	0	NF	0.682
36920	0.040	3.554	0.000	0	NF	0.708
36324	0.266	6.823	0.000	0	NF	0.708
16367	-0.007	0.404	0.000	1	NF	0.727
5479	-0.102	0.266	0.000	1	NF	0.737
6371	-0.666	0.000	8.802	0	NF	1.000

CID: compound ID; Class.: classification; C.L.: confidence level of the classification provided by the model; MATS2p: Moran Autocorrelation (2D) descriptor (weighted by atomic polarizabilities); SaasC: E-State Atom-Type Descriptor (Electrotopological State). Sum of E-State values for carbon atoms (C) in the topological environment "aas"; SsCl: E-State Atom-Type Descriptor. Sum of E-State values for chlorine atoms (Cl) in the topological environment "s"; NaaN: Atom-Centered Fragment (ACF) Descriptor. Count of nitrogen atoms (N) in the topological environment "aaN".

Table S16. Classification and probability of classification for internal test set compounds in Model 10 (ANN- Mordred) trained with WIPO data, focusing on Fungicide activity prediction.

CID	ZMIC2	Lipinski	n6HRing	Class.	C.L.
**Active Group**					
7744	59.1298	0	0	F	0.512
39385	28.9502	1	0	F	0.560
4921319	30.1400	1	0	F	0.591
9492	30.3267	1	0	F	0.596
7430	30.7500	1	0	F	0.605
135083	36.7220	1	1	F	0.606
39676	31.6658	1	0	F	0.621
8607	50.3225	1	0	F	0.628
14994	32.1241	1	0	F	0.628
6327657	58.7280	1	0	F	0.636
7511	44.1730	1	0	F	0.636
90545	42.6085	1	0	F	0.640
7627	42.3759	1	0	F	0.641
86173	33.4362	1	0	F	0.643
213032	41.8104	1	0	F	0.643
54682462	41.5526	1	0	F	0.643
17432	40.9237	1	0	F	0.645
11664966	36.1952	1	0	F	0.654
92200	43.0458	1	2	F	0.742
7156993	16.3477	1	0	NF	0.647
**Inactive Group**					
170317	29.7081	1	0	F	0.581
2708	30.4854	1	0	F	0.599
6	31.8585	1	0	F	0.624
168884	41.6226	1	1	F	0.709
10350985	100.6048	0	0	NF	0.522
73343	23.6878	1	0	NF	0.609
78782	22.8585	1	0	NF	0.628
154257	36.6170	0	0	NF	0.646
92189	21.4941	1	0	NF	0.651
216239	38.6134	0	1	NF	0.657
97663	18.5519	1	0	NF	0.665
457	17.3849	1	1	NF	0.686
130229	28.9088	1	1	NF	0.688
1456	23.8816	1	1	NF	0.745

CID: compound ID; Class.: classification; C.L.: confidence level of the classification provided by the model; ZMIC2: Zagreb Index (modified). Topological index emphasizing heteroatoms/bonds.; Lipinski: Drug-likeness rule. Binary flag for Rule of Five compliance. n6HRing: Heterocycle count; Number of 6-membered rings with heteroatoms.

Table S17. Classification and probability of classification for internal test set compounds in Model 11 (ANN- Mordred) trained with FRAC-WIPO data, focusing on Fungicide activity prediction.

CID	GATS3c	NssCH2	PEOE_VSA6	VSA_EState6	Class.	C.L.
**Active Group**						
tolprocarb	0.846	2	31.544	6.365	F	0.503
furalaxyl	0.914	0	18.199	8.157	F	0.563
isotianil	1.135	0	35.335	8.659	F	0.569
fluopicolide	1.030	1	40.869	5.311	F	0.623
pyrazophos	1.645	3	0.000	1.612	F	0.626
ipflufenoquin	1.448	0	6.066	8.089	F	0.648
isofetamid	1.640	0	0.000	7.307	F	0.663
oxytetracycline	0.948	0	12.133	2.537	F	0.666
fluoxastrobin	1.202	2	41.022	13.324	F	0.692
diniconazole	0.814	0	50.039	5.218	F	0.694
benodanil	1.266	0	30.332	16.962	F	0.704
phosphorous acid	1.574	0	0.000	0.000	F	0.717
fluindapyr	1.014	1	20.771	2.799	F	0.717
penthiopyrad	1.004	1	20.771	1.721	F	0.744
flusulfamide	0.838	0	23.202	4.993	F	0.747
pyriofenone	1.100	0	11.601	1.717	F	0.765
inpyrfluxam	1.008	1	32.904	5.793	F	0.820
laminarin	0.809	3	0.000	0.000	F	0.856
quinofumelin	1.250	0	42.465	16.287	F	0.865
chlozolinate	1.406	1	23.202	4.177	F	0.865
orysastrobin	1.037	1	44.888	7.154	F	0.876
fenpicoxamid	1.187	3	58.027	9.032	F	0.881
chloroneb	1.196	0	23.202	3.228	F	0.888
triadimenol	1.176	0	32.372	6.973	F	0.895
fenpyrazamine	1.262	1	42.103	7.332	F	0.900
valifenalate	1.252	1	37.581	5.356	F	0.930
vinclozoline	1.418	0	29.781	4.425	F	0.933
prochloraz	1.277	4	41.727	2.969	F	0.955
aldimorph	1.540	13	64.711	0.000	F	0.994
boscalid	1.193	0	53.534	18.295	F	1.000
fenamidone	1.203	0	60.293	19.291	F	1.000
fludioxonil	0.680	0	12.133	6.512	NF	0.758
cyproconazole	0.926	3	30.657	7.446	NF	0.771
metconazole	0.909	4	37.581	7.911	NF	0.824
mandipropamid	0.881	4	41.641	12.417	NF	0.904
prothioconazole	0.989	4	29.800	7.482	NF	0.914
flumorph	0.923	4	18.199	11.510	NF	1.000
biphenyl	0.911	0	60.664	20.781	NF	1.000
polyoxin	0.964	0	0.000	-1.257	NF	1.000
**Inactive Group**						
164867	1.031	0	11.601	5.812	F	0.577
2155	1.238	0	0.000	0.000	F	0.642

10245201	1.384	4	25.980	6.555	F	0.728
166553	1.239	2	18.525	6.825	F	0.811
91741	1.130	0	29.268	2.923	F	0.912
2081	1.526	1	23.734	6.883	F	0.960
10090	1.058	3	67.587	20.399	F	1.000
83813	1.031	2	60.132	17.208	F	1.000
91683	0.922	0	41.933	12.588	NF	0.519
10124	0.937	0	11.337	5.996	NF	0.521
216239	1.246	0	11.601	11.597	NF	0.526
196968	0.983	0	38.113	12.487	NF	0.554
10331863	0.935	0	11.987	8.037	NF	0.609
76962	0.888	1	6.924	0.000	NF	0.652
2048	1.095	0	30.332	14.332	NF	0.659
123619	1.114	0	23.734	12.378	NF	0.758
153103	0.993	2	6.924	0.000	NF	0.796
15851	0.932	4	36.221	7.616	NF	0.830
12447	0.828	0	30.341	15.680	NF	0.857
10313100	1.960	0	0.000	0.000	NF	0.865
126970	1.133	4	17.667	6.603	NF	0.877
159599	1.169	3	0.000	3.513	NF	0.952
13268	1.183	4	0.000	0.000	NF	0.963
15939	0.806	0	0.000	8.481	NF	0.969
11960529	0.933	3	6.066	5.436	NF	0.983
115239	0.849	0	0.000	11.091	NF	0.985
132260161	0.908	2	0.000	3.286	NF	0.989
1930	0.805	14	70.442	0.000	NF	0.991
10547	0.428	4	0.000	0.000	NF	0.993
100516	0.823	4	12.133	5.350	NF	0.996
11689883	1.113	5	12.133	7.861	NF	0.999
161240	0.770	5	18.199	7.041	NF	1.000
107896	0.880	3	0.000	10.921	NF	1.000
11291932	1.027	6	24.265	11.798	NF	1.000
2448	1.140	7	28.063	13.582	NF	1.000
2293	1.118	10	24.265	12.671	NF	1.000

CID: compound ID; Class.: classification; C.L.: confidence level of the classification provided by the model; GATS3c: 2D Geary Autocorrelation Index. Charge similarity at 3-bond distance; NssCH2: Atom-Centered Fragment Count Index. Number of sp3 methylene groups; PEOE_VSA6: Partial Charge Surface Area Descriptor. Surface area with moderate positive charges; VSA_EState6: E-State/Van der Waals Surface Area Hybrid Descriptor. Surface area of intermediate E-State atoms.

Table S18. Classification and probability of classification for internal test set compounds in Model 12 (ANN- Mordred) trained with Pubchem-WIPO data, focusing on Fungicide activity prediction.

CID	EState_VSA4	nN	VSA_EState4	Class.	C.L.
**Active Group**					
17581	0.000	0	0.000	1	0.624
86173	0.000	3	0.715	1	0.651
8607	4.305	1	0.656	1	0.682
41368	0.000	3	-0.358	1	0.722
11292824	5.563	1	1.654	1	0.727
25429	11.033	3	1.679	1	0.736
5430	22.552	3	4.750	2	0.719
**Inactive Group**					
6371	0.000	0	0.000	1	0.624
7568320	6.545	1	0.000	2	0.555
10461	23.266	0	0.000	2	0.561
9838802	31.371	1	0.065	2	0.706
6917865	29.433	1	0.809	2	0.716
16351	48.288	3	2.182	2	0.719
16367	0.000	4	8.001	2	0.719
2955	0.000	2	12.092	2	0.719

CID: compound ID; Class.: classification; C.L.: confidence level of the classification provided by the model; EState_VSA4: Electrotopological State (E-State) + Van der Waals Surface Area (VSA) Hybrid Descriptor. Portion of molecular Van der Waals surface area where atoms have E-State values in bin 4; VSA_EState4: absolute Van der Waals surface area (Å²) of atoms with E-State values in bin 4; nN: Total number of nitrogen atoms in the molecule.

Table S19. Classification and probability of classification for training set compounds in Model 13 (LDA-AlvaDesc), focusing on Acid Phosphatase Inhibitory activity prediction.

CID	MATS2m	MATS4i	nOHp	SsOH	DF	Class.	P.C.
**Active Group**							
diethyl(dodecylsulfonamido(4-methoxyphenyl)methyl)phosphonate	0.041	-0.105	0	0.000	-2.650	I	0.934
bromoenol lactone	-0.056	-0.149	0	0.000	-1.382	I	0.799
d-erythro-sphingosine	-0.191	0.014	1	18.313	0.021	API	0.506
gossypol AcA	0.265	-0.077	0	73.042	0.632	API	0.655
p-tolyl thiazole carboxylic acid	-0.042	0.035	0	8.811	2.039	API	0.885
trimyristin	-0.128	0.000	0	0.000	2.351	API	0.913
desipramine	-0.035	0.137	0	0.000	2.565	API	0.929
d-propanolol	-0.017	0.105	0	9.844	2.696	API	0.937
glutathione	-0.068	-0.002	0	16.950	2.828	API	0.944
l-(+)-tartaric acid	-0.047	-0.048	0	32.526	3.279	API	0.964
tunicamycin	-0.022	0.010	1	86.336	3.398	API	0.968
(1-naphthylmethyl)phosphonic acid	-0.217	-0.154	0	17.883	3.598	API	0.973
okadaic acid	0.098	0.030	0	54.012	3.705	API	0.976
d-myoinositol-hexasulphate	-0.187	-0.171	0	53.539	6.346	API	0.998
**Inactive Group**							
400769	0.242	-0.053	0	0.000	-5.914	I	0.997
5370221	0.154	-0.108	0	0.000	-4.984	I	0.993
3035284	0.095	-0.196	0	10.100	-4.109	I	0.984
9903786	0.142	-0.054	0	0.000	-3.925	I	0.981
21124830	0.090	-0.138	0	10.535	-3.083	I	0.956
24937227	0.106	0.000	0	0.000	-2.374	I	0.915
11915	-0.045	-0.248	0	8.286	-2.262	I	0.906
5490068	0.053	-0.177	0	21.214	-1.863	I	0.865
11541511	0.095	-0.123	0	23.033	-1.700	I	0.845
446440	-0.059	0.021	1	28.183	-1.538	I	0.823
20179	0.017	-0.062	0	0.000	-1.523	I	0.821
4671	-0.191	0.001	1	8.585	-1.171	I	0.763
4908	0.024	-0.029	0	0.000	-1.157	I	0.761
5601	0.030	-0.018	0	0.000	-1.120	I	0.754
11500504	0.070	-0.083	0	24.412	-0.452	I	0.610
6918295	0.154	0.133	0	13.278	0.033	API	0.509
60916	-0.042	-0.053	0	14.986	1.310	API	0.788

CID: compound ID; API: acid phosphatase inhibitor; I: inactive. DF: discriminant function; P.C.: probability of classification; MATS2m: Moran autocorrelation at lag 2 weighted by atomic mass; MATS4i: Moran autocorrelation at lag 4 weighted by ionization potential; nOHp: Number of primary hydroxyl groups (-CH_2OH); SsOH: Sum of E-State values for hydroxyl groups connected by single bonds.

Table S20. Classification and probability of classification for training set compounds in Model 14 (ANN-AlvaDesc), focusing on Acid Phosphatase Inhibitory activity prediction.

CID	MATS2m	MATS4i	nOHp	SsOH	Class.	C.L.
**Active Group**						
diethyl(dodecylsulfonamido(4-methoxyphenyl)methyl)phosphonate	0.041	-0.105	0	0.000	API	0.557
(1-naphthylmethyl)phosphonic acid	-0.217	-0.154	0	17.883	API	0.985
p-tolyl thiazole carboxylic acid	-0.042	0.035	0	8.811	API	0.998
bromoenol lactone	-0.056	-0.149	0	0.000	API	0.999
glutathione	-0.068	-0.002	0	16.950	API	1.000
trimyristin	-0.128	0.000	0	0.000	API	1.000
desipramine	-0.035	0.137	0	0.000	API	1.000
d-erythro-sphingosine	-0.191	0.014	1	18.313	API	1.000
d-propanolol	-0.017	0.105	0	9.844	API	1.000
okadaic acid	0.098	0.030	0	54.012	API	1.000
gossypol AcA	0.265	-0.077	0	73.042	API	1.000
tunicamycin	-0.022	0.010	1	86.336	API	1.000
l-(+)-tartaric acid	-0.047	-0.048	0	32.526	API	1.000
d-myoinositol-hexasulphate	-0.187	-0.171	0	53.539	API	1.000
**Inactive Group**						
11915	-0.045	-0.248	0	8.286	I	0.706
20179	0.017	-0.062	0	0.000	I	0.728
4908	0.024	-0.029	0	0.000	I	0.978
5601	0.030	-0.018	0	0.000	I	0.994
5370221	0.154	-0.108	0	0.000	I	0.998
60916	-0.042	-0.053	0	14.986	I	1.000
9903786	0.142	-0.054	0	0.000	I	1.000
3035284	0.095	-0.196	0	10.100	I	1.000
24937227	0.106	0.000	0	0.000	I	1.000
21124830	0.090	-0.138	0	10.535	I	1.000
400769	0.242	-0.053	0	0.000	I	1.000
5490068	0.053	-0.177	0	21.214	I	1.000
4671	-0.191	0.001	1	8.585	I	1.000
6918295	0.154	0.133	0	13.278	I	1.000
446440	-0.059	0.021	1	28.183	I	1.000
11541511	0.095	-0.123	0	23.033	I	1.000
11500504	0.070	-0.083	0	24.412	I	1.000

CID: compound ID; Class.: classification; C.L.: confidence level of the classification provided by the model; API: Acid phosphatase inhibitor; I: inactive; MATS2m: Moran autocorrelation at lag 2 weighted by atomic mass; MATS4i: Moran autocorrelation at lag 4 weighted by ionization potential; nOHp: Number of primary hydroxyl groups (-CH_2OH); SsOH: Sum of E-State values for hydroxyl groups connected by single bonds.

Table S21. Classification and probability of classification for training set compounds in Model 15 (ANN80-AlvaDesc), focusing on Acid Phosphatase Inhibitory activity prediction.

CID	MATS2m	MATS4i	nOHp	SsOH	Class.	C.L.
**Active Group**						
diethyl(dodecylsulfonamido(4-methoxyphenyl)methyl)phosphonate	0.041	-0.105	0	0.000	API	0.519
p-tolyl thiazole carboxylic acid	-0.042	0.035	0	8.811	API	0.644
bromoenol lactone	-0.056	-0.149	0	0.000	API	0.683
gossypol AcA	0.265	-0.077	0	73.042	API	0.691
tunicamycin	-0.022	0.010	1	86.336	API	0.708
(1-naphthylmethyl)phosphonic acid	-0.217	-0.154	0	17.883	API	0.712
d-myoinositol-hexasulphate	-0.187	-0.171	0	53.539	API	0.719
desipramine	-0.035	0.137	0	0.000	API	0.732
glutathione	-0.068	-0.002	0	16.950	API	0.732
okadaic acid	0.098	0.030	0	54.012	API	0.735
d-propanolol	-0.017	0.105	0	9.844	API	0.742
l-(+)-tartaric acid	-0.047	-0.048	0	32.526	API	0.749
**Inactive Group**						
9903786	0.142	-0.054	0	0.000	I	0.560
21124830	0.090	-0.138	0	10.535	I	0.605
20179	0.017	-0.062	0	0.000	I	0.631
11915	-0.045	-0.248	0	8.286	I	0.678
6918295	0.154	0.133	0	13.278	I	0.691
60916	-0.042	-0.053	0	14.986	I	0.693
400769	0.242	-0.053	0	0.000	I	0.723
446440	-0.059	0.021	1	28.183	I	0.727
5370221	0.154	-0.108	0	0.000	I	0.728
5490068	0.053	-0.177	0	21.214	I	0.733
4671	-0.191	0.001	1	8.585	I	0.735
24937227	0.106	0.000	0	0.000	I	0.750
3035284	0.095	-0.196	0	10.100	I	0.756

CID: compound ID; Class.: classification; C.L.: confidence level of the classification provided by the model; API: Acid phosphatase inhibitor; I: inactive; MATS2m: Moran autocorrelation at lag 2 weighted by atomic mass; MATS4i: Moran autocorrelation at lag 4 weighted by ionization potential; nOHp: Number of primary hydroxyl groups (-CH_2OH); SsOH: Sum of E-State values for hydroxyl groups connected by single bonds.

Table S22. Classification and probability of classification for training set compounds in Model 16 (LDA-Mordred), focusing on Acid Phosphatase Inhibitory activity prediction.

CID	NdssC	ATSC3c	DF	Class.	P.A.
**Active Group**					
(1-naphthylmethyl)phosphonic_acid	0	-0.311	-0.044	I	0.511
trimyristin	3	0.290	-0.375	I	0.592
bromoenol_lactone	2	-0.032	-0.771	I	0.684
d-myoinositol-hexasulphate	0	-0.548	-1.028	I	0.737
pa inhibitor	1	-0.044	0.122	API	0.530
fluopyram	1	0.028	0.419	API	0.603
glutathione	4	0.764	0.646	API	0.656
l-(+)-tartaric_acid	2	0.385	0.961	API	0.723
desipramine	0	0.042	1.418	API	0.805
gossypol_aca	1	0.333	1.687	API	0.844
okadaic_acid	3	0.795	1.719	API	0.848
diethyl(dodecylsulfonamido(4-methoxyphenyl)methyl)phosphonate	0	0.120	1.745	API	0.851
d-propanolol	0	0.223	2.172	API	0.898
d-erythro-sphingosine	0	0.239	2.238	API	0.904
tunicamycin	2	0.794	2.658	API	0.935
**Inactive Group**					
11915	2	0.175	0.089	API	0.522
24937227	1	0.000	0.304	API	0.576
446440	2	0.315	0.671	API	0.662
4671	1	0.097	0.706	API	0.670
4908	0	-0.042	1.073	API	0.745
5490068	0	0.128	1.776	API	0.855
5601	2	0.108	-0.189	I	0.547
20179	1	-0.134	-0.251	I	0.562
3035284	2	-0.030	-0.762	I	0.682
6918295	3	0.020	-1.498	I	0.817
11541511	3	-0.104	-2.012	I	0.882
60916	4	0.082	-2.184	I	0.899
21124830	5	0.217	-2.562	I	0.928
9903786	2	-0.538	-2.871	I	0.946
11500504	4	-0.123	-3.032	I	0.954
400769	5	0.088	-3.100	I	0.957
5370221	5	-0.153	-4.098	I	0.984

CID: compound ID; Class.: classification; API: acid phosphatase inhibitor; I: inactive; P.A.: probability of activity; NdssC: number of carbon atoms with exactly two single bonds and one double bond (sp^2 hybridized) in a specific environment; ATSC3c: Centered Broto-Moreau autocorrelation at lag 3 weighted by atomic charges.

Table S23. Classification and probability of classification for training set compounds in Model 17 (ANN-Mordred), focusing on Acid Phosphatase Inhibitory activity prediction.

CID	Class.	C.L.
**Active Group**		
Bromoenol_lactone	API	0.548
Diethyl(dodecylsulfonamido(4-methoxyphenyl)methyl)phosphonate	API	0.582
fluopyram	API	0.723
PA inhibitor	API	0.825
desipramine	API	0.988
~~desipramine~~	~~API~~	~~0.988~~
(1-Naphthylmethyl)phosphonic_acid	API	0.994
gossypol_AcA	API	0.998
D-Propanolol	API	1.000
D-myoinositol-hexasulphate	API	1.000
D-erythro-Sphingosine	API	1.000
trimyristin	API	1.000
glutathione	API	1.000
L-(+)-Tartaric_acid	API	1.000
okadaic_acid	API	1.000
tunicamycin	API	1.000
**Inactive Group**		
5490068	I	0.517
3035284	I	0.608
24937227	I	0.773
4671	I	0.891
4908	I	0.933
9903786	I	0.985
20179	I	1.000
446440	I	1.000
11500504	I	1.000
11541511	I	1.000
11915	I	1.000
21124830	I	1.000
400769	I	1.000
5370221	I	1.000
5601	I	1.000
60916	I	1.000
6918295	I	1.000

CID: compound ID; Class.: classification; C.L.: confidence level of the classification provided by the model; API: Acid phosphatase inhibitor; I: inactive.

Table S24. Classification and probability of classification for training set compounds in Model 18 (ANN80-Mordred), focusing on Acid Phosphatase Inhibitory activity prediction.

CID	NdssC	ATSC3c	Class.	C.L.
**Active Group**				
l-(+)-tartaric_acid	2	0.385	API	0.959
(1-naphthylmethyl)phosphonic_acid	0	-0.311	API	1.000
desipramine	0	0.042	API	1.000
d-erythro-sphingosine	0	0.239	API	1.000
diethyl(dodecylsulfonamido(4-methoxyphenyl)methyl)phosphonate	0	0.120	API	1.000
d-myoinositol-hexasulphate	0	-0.548	API	1.000
d-propanolol	0	0.223	API	1.000
d-erythro-sphingosine	0	0.239	API	1.000
desipramine	0	0.042	API	1.000
diethyl(dodecylsulfonamido(4-methoxyphenyl)methyl)phosphonate	0	0.120	API	1.000
glutathione	4	0.764	API	1.000
okadaic_acid	3	0.795	API	1.000
trimyristin	3	0.290	API	1.000
tunicamycin	2	0.794	API	1.000
bromoenol_lactone	2	-0.032	I	0.613
pa inhibitor	1	-0.044	I	0.637
fluopyram	1	0.028	I	0.654
**Inactive group**				
20179	1	-0.134	I	0.580
4671	1	0.097	I	0.638
24937227	1	0.000	I	0.651
9903786	2	-0.538	I	0.878
11915	2	0.175	I	0.975
6918295	3	0.020	I	1.000
11500504	4	-0.123	I	1.000
21124830	5	0.217	I	1.000
400769	5	0.088	I	1.000
5370221	5	-0.153	I	1.000
60916	4	0.082	I	1.000

CID: compound ID; Class.: classification; API: acid phosphatase inhibitor; I: inactive; C.L.: confidence level of the classification provided by the model; NdssC: Number of carbon atoms with exactly two single bonds and one double bond (sp^2 hybridized) in a specific environment; ATSC3c: Centered Broto-Moreau autocorrelation at lag 3 weighted by atomic charges.

Table S25. Classification and probability of classification for internal test set compounds in Model 15 (ANN80-AlvaDesc), focusing on Acid Phosphatase Inhibitory activity prediction.

CID	MATS2m	MATS4i	nOHp	SsOH	Class.	C.L.
**Active Group**						
trimyristin	-0.128	0.000	0	0.000	API	0.587
**Inactive Group**						
4908	0.024	-0.029	0	0.000	I	0.828
5601	0.030	-0.018	0	0.000	I	0.847
11541511	0.095	-0.123	0	23.033	I	0.868
11500504	0.070	-0.083	0	24.412	I	0.872

CID: compound ID; Class.: classification; C.L.: confidence level of the classification provided by the model; API: acid phosphatase inhibitor; I: inactive. MATS2m: Moran autocorrelation at lag 2 weighted by atomic mass; MATS4i: Moran autocorrelation at lag 4 weighted by ionization potential; nOHp: Number of primary hydroxyl groups (-CH_2OH); SsOH: Sum of E-State values for hydroxyl groups connected by single bonds.

Table S26. Classification and probability of classification for internal test set compounds in Model 18 (ANN80-Mordred), focusing on Acid Phosphatase Inhibitory activity prediction.

CID	NdssC	ATSC3c	Class.	C.L.
**Active Group**				
gossypol_aca	1	0.333	API	0.827
**Inactive Group**				
4908	0	-0.042	API	1.000
5490068	0	0.128	API	1.000
3035284	2	-0.030	I	0.622
446440	2	0.315	I	0.854
5601	2	0.108	I	0.947
11541511	3	-0.104	I	1.000

CID: compound ID; C.L.: confidence level of the classification provided by the model; API: acid phosphatase inhibitor; I: inactive; NdssC: Number of carbon atoms with exactly two single bonds and one double bond (sp^2 hybridized) in a specific environment; ATSC3c: Centered Broto-Moreau autocorrelation at lag 3 weighted by atomic charges.

Table S27. First virtual screening selection of potential **F**ungicides targeting **A**cid **P**hosphatase **I**nhibition) (**FAPI**).

New code	IUPAC name	Chemical structure
FAPI-I-01	Triphenyl phosphate	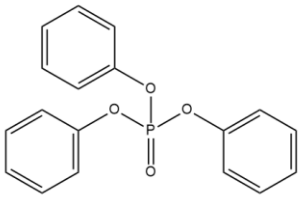
FAPI-I-02	2,2-bis(hydroxymethyl)propane-1,3-diol	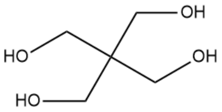
FAPI-I-03	6-oxa-3-thiabicyclo[3.1.0]hexane 3,3-dioxide	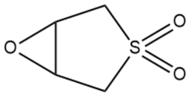
FAPI-I-04	Triphenyl phosphite	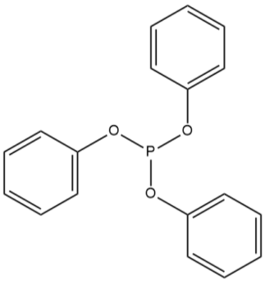
FAPI-I-05	2,3,4,6,7,8-hexahydropyrrolo[1,2-a]pyrimidine	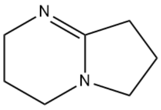
FAPI-I-06	2-methyl-1,3-dithiane	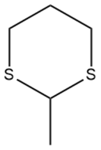
FAPI-I-07	Morpholin-4-amine	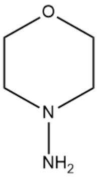
FAPI-I-08	(1r,3r,5r,7r)-adamantan-2-one	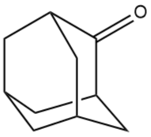

FAPI-I-09	2-methylhexahydro-1H-4,7-epoxyisoindole-1,3(2H)-dione	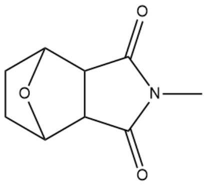
FAPI-I-10	1,4,4a,5,8,8a,9a,10a-octahydro-1,4:5,8-dimethanoanthracene-9,10-dione	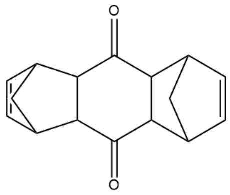
FAPI-I-11	Morpholine-4-carbaldehyde	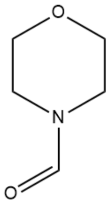
FAPI-I-12	4-(2-chloroethyl)morpholine	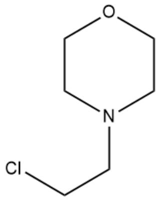
FAPI-I-13	Cyclohexane-1,2,3,4,5,6-hexayl hexakis(dihydrogen phosphate)	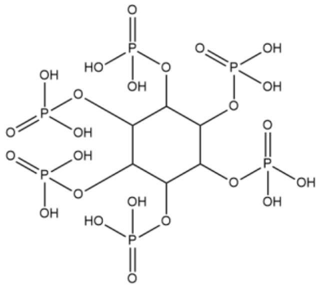
FAPI-I-14	(4R,7S,7aR)-2-bromo-3-hydroxy-3a,4,7,7a-tetrahydro-1H-4,7-methanoinden-1-one	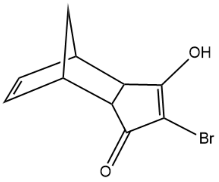
FAPI-I-15	6,7-dihydroimidazo[1,2-a]pyridin-8(5H)-one	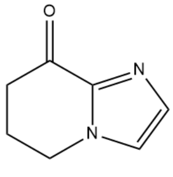

FAPI-I-16	(3aR,4S,7R,7aS)-3a,7a-dimethylhexahydro-4,7-epoxyisobenzofuran-1,3-dione	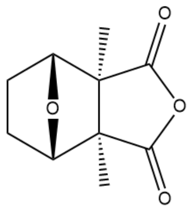
FAPI-I-17	Diphenyl (4-bromophenyl)phosphoramidate	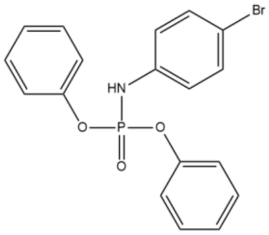
FAPI-I-18	6,7-dihydroimidazo[1,2-a]pyridin-8(5H)-one	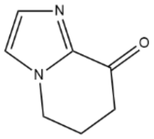
FAPI-I-19	(5S,6S,9R,10S)-2,3,6,7-tetrahydro-5H-6,9-epoxy-5,10-methanothiazolo[2,3-e][1,4,6]oxadiazonin-10(9H)-ol	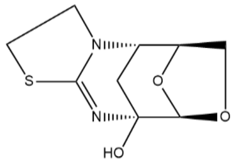
FAPI-I-20	(5S,6R,9S,10S)-9,10-dihydro-4H,8H-6,9-epoxy-5,10-methanotetrazolo[5,1-e][1,4,6]oxadiazonin-5(6H)-ol	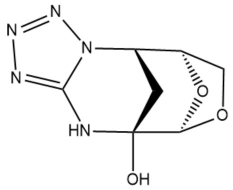

Table S28. Classification and Probability of Classification for the First Virtual Screening Focused on Fungicide Activity Prediction as Part of the *In Silico* Strategy to Identify Novel FAPI Compounds Using AlvaDesc QSAR Models.

Compound	Model 1 LDA			Model 2 ANN		Model 3 LDA			Model 4 ANN		Model 5 LDA			Model 6 ANN	
	DF_1	Cl.	P.A.	Cl.	C.L.	DF_3	Cl.	P.A.	Cl.	C.L.	DF_5	Cl.	P.A.	Cl.	C.L.
fluopyram	-0.991	NF	0.275	F	0.505	-0.349	NF	0.410	NF	0.547	3.054	F	0.955	F	0.730
FAPI-I-01	-1.493	NF	0.188	F	0.517	0.341	F	0.583	F	0.513	-1.549	NF	0.175	NF	0.620
FAPI-I-02	-0.841	NF	0.301	NF	0.565	1.767	F	0.854	F	0.713	0.160	F	0.540	NF	0.664
FAPI-I-03	-3.275	NF	0.036	NF	0.648	0.194	F	0.548	F	0.539	-1.990	NF	0.120	F	0.955
FAPI-I-04	-1.493	NF	0.188	F	0.517	0.520	F	0.624	F	0.501	-2.135	NF	0.106	NF	0.708
FAPI-I-05	-2.736	NF	0.061	NF	0.619	-1.331	NF	0.209	NF	0.696	-1.111	NF	0.248	NF	0.655
FAPI-I-06	-3.275	NF	0.036	NF	0.648	1.487	F	0.812	F	0.612	-0.906	NF	0.288	NF	0.736
FAPI-I-07	-3.275	NF	0.036	NF	0.648	-0.620	NF	0.350	NF	0.629	-0.923	NF	0.284	NF	0.733
FAPI-I-08	-6.275	NF	0.002	NF	0.645	0.109	F	0.527	F	0.559	-1.105	NF	0.249	NF	0.658
FAPI-I-09	-2.176	NF	0.102	NF	0.588	-0.169	NF	0.458	NF	0.549	-0.509	NF	0.375	F	0.835
FAPI-I-10	-3.015	NF	0.047	NF	0.544	0.109	F	0.527	F	0.559	-1.195	NF	0.232	NF	0.626
FAPI-I-11	-3.010	NF	0.047	NF	0.634	-0.910	NF	0.287	NF	0.692	-0.384	NF	0.405	F	0.783
FAPI-I-12	-2.146	NF	0.105	NF	0.586	-1.982	NF	0.118	F	0.548	-0.379	NF	0.406	NF	0.727
FAPI-I-13	-0.911	NF	0.287	NF	0.518	0.373	F	0.590	F	0.509	-1.064	NF	0.256	NF	0.675
FAPI-I-14	-1.545	NF	0.177	NF	0.561	-0.551	NF	0.366	F	0.569	-1.034	NF	0.262	NF	0.689
FAPI-I-15	-2.953	NF	0.050	NF	0.631	0.109	F	0.527	F	0.559	-0.068	NF	0.483	NF	0.760
FAPI-I-16	-2.136	NF	0.106	NF	0.585	1.061	F	0.743	F	0.526	0.174	F	0.543	NF	0.668
FAPI-I-17	-1.037	NF	0.268	F	0.534	-0.985	NF	0.271	NF	0.572	-2.174	NF	0.102	NF	0.708
FAPI-I-18	-2.953	NF	0.050	NF	0.631	0.109	F	0.527	F	0.559	-0.068	NF	0.483	NF	0.760
FAPI-I-19	-2.103	NF	0.110	NF	0.545	-1.021	NF	0.262	NF	0.702	-1.091	NF	0.251	NF	0.664
FAPI-I-20	-0.130	NF	0.469	F	0.511	0.475	F	0.616	F	0.681	2.875	F	0.947	F	1.000

D.F.: discriminant function; P.A.: probability of being active as fungicide by LDA model; Cl.: Classification; C.L.: confidence level of the ANN classification; F: fungicide; NF: no fungicide.

Table S29. Classification and Probability of Classification for the First Virtual Screening Focused on Fungicide Activity Prediction as Part of the *In Silico* Strategy to Identify Novel FAPI Compounds Using Mordred QSAR Models.

Compound	Model 7 LDA			Model 8 ANN		Model 9 LDA			Model 10 ANN		Model 11 LDA			Model 12 ANN	
	DF_7	Class.	P.A.	Class.	C.L.	DF_9	Class.	PA	Class.	C.L.	DF_{11}	Class.	P.A.	Class.	C.L.
fluopyram	0.962	F	0.727	F	0.752	0.074	F	0.518	F	0.803	1.956	F	0.876	F	0.800
FAPI-I-01	0.260	F	0.570	F	0.518	0.617	F	0.649	NF	1.000	0.601	F	0.646	F	0.624
FAPI-I-02	0.525	F	0.631	F	0.637	-1.939	NF	0.126	NF	0.834	1.258	F	0.779	F	0.825
FAPI-I-03	-0.208	NF	0.450	NF	0.556	-0.043	NF	0.489	NF	0.796	0.601	F	0.646	F	0.624
FAPI-I-04	0.210	F	0.557	F	0.515	0.293	F	0.573	NF	1.000	0.601	F	0.646	F	0.624
FAPI-I-05	-1.928	NF	0.128	NF	0.696	-1.283	NF	0.217	NF	0.557	1.910	F	0.871	F	0.621
FAPI-I-06	-1.280	NF	0.219	NF	0.744	-0.171	NF	0.457	NF	0.628	0.024	F	0.506	NF	0.783
FAPI-I-07	-1.864	NF	0.135	NF	0.706	-1.030	NF	0.263	F	0.781	-0.579	NF	0.360	NF	0.719
FAPI-I-08	-0.022	NF	0.497	F	0.508	-1.878	NF	0.133	NF	0.925	-0.890	NF	0.291	NF	0.863
FAPI-I-09	-0.036	NF	0.493	F	0.503	0.143	F	0.536	NF	0.720	0.050	F	0.513	F	0.720
FAPI-I-10	1.492	F	0.819	F	0.640	1.140	F	0.758	F	0.745	-1.017	NF	0.266	NF	0.861
FAPI-I-11	-1.746	NF	0.149	NF	0.721	-1.524	NF	0.179	F	0.662	-0.789	NF	0.313	NF	0.724
FAPI-I-12	-1.242	NF	0.226	NF	0.743	-1.773	NF	0.145	NF	0.959	-3.212	NF	0.039	NF	0.629
FAPI-I-13	7.401	F	0.999	NF	0.665	0.105	F	0.526	NF	0.559	0.601	F	0.646	F	0.624
FAPI-I-14	0.089	F	0.525	F	0.544	0.240	F	0.560	NF	0.539	-0.208	NF	0.448	NF	0.748
FAPI-I-15	-1.989	NF	0.121	NF	0.686	-1.334	NF	0.208	F	0.901	1.101	F	0.751	F	0.724
FAPI-I-16	0.213	F	0.556	F	0.580	-0.030	NF	0.492	NF	0.791	-0.139	NF	0.466	F	0.699
FAPI-I-17	-0.989	NF	0.274	NF	0.578	0.883	F	0.707	NF	1.000	0.730	F	0.675	F	0.681
FAPI-I-18	-1.989	NF	0.121	NF	0.686	-1.334	NF	0.208	F	0.901	1.101	F	0.751	F	0.724
FAPI-I-19	-2.525	NF	0.075	NF	0.676	-0.592	NF	0.356	NF	0.888	1.207	F	0.770	F	0.646
FAPI-I-20	-2.575	NF	0.071	NF	0.671	-0.233	NF	0.442	NF	0.848	6.649	F	0.999	F	0.850

DF: discriminant function; P.A.: probability of being active as fungicide by LDA model; Cl.: Classification. C.L.: confidence level of the ANN classification. F: fungicide; NF: no fungicide.

Table S30. Classification and Probability of Classification for the First Virtual Screening Focused on Acid Phosphatase Inhibitory activity prediction as Part of the *In Silico* Strategy to Identify Novel FAPI Compounds Using AlvaDesc and Mordred QSAR Models.

Compound	AlvaDesc							Mordred						
	Model 13 LDA			Model 14 ANN (100)		Model 15 ANN		Model 16 LDA			Model 17 ANN		Model 18 ANN	
	DF_{13}	Class.	P.A.	Class	C.L.	Class.	C.L.	DF_{16}	Class.	P.A.	Class.	C.L.	Class.	C.L.
FAPI-I-01	-4.389	I	0.012	API	0.998	I	0.790	-0.663	I	0.340	API	1.000	API	1.000
FAPI-I-02	-14.520	I	0.000	I	1.000	API	0.726	1.089	API	0.748	I	0.869	API	1.000
FAPI-I-03	13.852	API	1.000	API	1.000	API	0.703	0.330	API	0.582	I	1.000	API	1.000
FAPI-I-04	-2.354	I	0.087	API	0.971	API	0.528	-0.338	I	0.416	API	1.000	API	1.000
FAPI-I-05	1.922	API	0.872	API	1.000	API	0.624	0.079	API	0.520	API	0.534	I	0.632
FAPI-I-06	-5.674	I	0.003	API	1.000	I	0.853	1.162	API	0.762	API	0.725	API	1.000
FAPI-I-07	1.309	API	0.787	API	1.000	I	0.778	1.032	API	0.737	I	0.990	API	1.000
FAPI-I-08	0.719	API	0.672	API	0.961	I	0.816	0.401	API	0.599	API	0.640	I	0.654
FAPI-I-09	-2.470	I	0.078	I	0.989	API	0.519	-2.176	I	0.102	API	0.914	API	0.827
FAPI-I-10	1.845	API	0.864	API	1.000	API	0.548	-1.086	I	0.253	API	1.000	API	0.672
FAPI-I-11	-0.692	I	0.334	API	1.000	I	0.506	1.586	API	0.830	API	0.941	API	1.000
FAPI-I-12	-1.747	I	0.148	API	1.000	I	0.713	1.381	API	0.799	API	0.989	API	1.000
FAPI-I-13	14.248	API	1.000	API	1.000	API	0.703	-6.670	I	0.001	API	1.000	API	0.996
FAPI-I-14	1.162	API	0.762	I	1.000	I	0.600	-2.123	I	0.107	I	1.000	I	1.000
FAPI-I-15	-0.551	I	0.366	I	0.941	I	0.863	1.050	API	0.741	I	1.000	I	0.558
FAPI-I-16	-8.137	I	0.000	I	0.620	I	0.851	-2.086	I	0.111	API	1.000	API	0.852
FAPI-I-17	-4.064	I	0.017	I	1.000	I	0.810	-1.052	I	0.259	API	1.000	API	1.000
FAPI-I-18	-0.551	I	0.366	I	0.941	I	0.863	1.050	API	0.741	I	1.000	I	0.558
FAPI-I-19	1.574	API	0.829	I	0.957	API	0.531	1.459	API	0.811	I	1.000	API	0.666
FAPI-I-20	-3.378	I	0.033	I	0.997	I	0.765	2.208	API	0.901	API	1.000	API	1.000
L-(+)-tartaric acid	3.279	API	0.964	API	1	API	0.749	0.961	API	0.723	API	1.000	API	0.959

D.F.: discriminant function; P.A.: probability of being active as fungicide by LDA model; Cl.: Classification; C.L.: confidence level of the ANN classification; F: fungicide; NF: no fungicide.

Table S31. Classification and probability of classification for training set compounds in Model 19 (LDA-AlvaDesc) trained with experimental fungicide activity data, focusing on Fungicide activity prediction.

FAPI	GATS3m	MATS6m	mindssC	DF	Class.	C.L.
**Active Group**						
FAPI-I-17	1.005	-0.018	0.000	-0.964	NF	0.724
FAPI-I-13	1.024	0.257	0.000	0.550	F	0.634
FAPI-I-14	0.615	-0.862	0.097	3.162	F	0.959
FAPI-I-12	0.655	-0.177	0.000	7.792	F	1.000
FAPI-I-16	0.896	0.475	-0.405	8.607	F	1.000
FAPI-I-15	0.947	1.216	0.177	8.673	F	1.000
FAPI-I-18	0.947	1.216	0.177	8.673	F	1.000
FAPI-I-02	0.853	0.720	0.000	8.867	F	1.000
**Inactive Group**						
FAPI-I-06	1.500	0.467	0.000	-11.390	NF	1.000
FAPI-I-07	1.292	0.000	0.000	-8.959	NF	1.000
FAPI-I-05	1.104	0.743	1.376	-7.593	NF	0.999
FAPI-I-19	0.940	-0.177	0.970	-7.013	NF	0.999
FAPI-I-10	1.069	-0.113	0.429	-6.446	NF	0.998
FAPI-I-08	1.120	0.309	0.622	-6.099	NF	0.998
FAPI-I-09	1.048	-0.486	-0.040	-5.380	NF	0.995
FAPI-I-04	1.013	-0.379	0.000	-3.863	NF	0.979
FAPI-I-01	0.975	-0.424	0.000	-3.149	NF	0.959
FAPI-I-11	1.085	0.000	0.000	-3.085	NF	0.956
FAPI-I-03	1.076	0.000	0.000	-2.846	NF	0.945
FAPI-I-20	1.089	0.135	0.000	-2.207	NF	0.901

FAPI: fungal acid phosphatase inhibitor; DF: discriminant function; C.L.: confidence level of the classification provided by the model; Class.: classification; GATS3m: Geary's autocorrelation at topological lag 3 weighted by atomic masses; MATS6m: Moran's autocorrelation at lag 6 weighted by atomic masses; mindssC: Minimum E-State value for doubly bonded sp^2 carbons (=C<).

Table S32. Classification and probability of classification for training set compounds in Model 20 (ANN-AlvaDesc) trained with experimental fungicide activity data, focusing on Fungicide activity prediction.

Compound	MATS6m	GATS3m	mindssC	Class.	C.L.
**Active Group**					
FAPI-I-17	-0.018	1.005	0.000	F	0.885
FAPI-I-13	0.257	1.024	0.000	F	0.999
FAPI-I-14	-0.862	0.615	0.097	F	1.000
FAPI-I-12	-0.177	0.655	0.000	F	1.000
FAPI-I-15	1.216	0.947	0.177	F	1.000
FAPI-I-2	0.720	0.853	0.000	F	1.000
FAPI-I-16	0.475	0.896	-0.405	F	1.000
**Inactive Group**					
FAPI-I-20	0.135	1.089	0.000	NF	0.761
FAPI-I-03	0.000	1.076	0.000	NF	0.965
FAPI-I-1	-0.424	0.975	0.000	NF	0.995
FAPI-I-04	-0.379	1.013	0.000	NF	0.999
FAPI-I-09	-0.486	1.048	-0.040	NF	1.000
FAPI-I-10	-0.113	1.069	0.429	NF	1.000
FAPI-I-19	-0.177	0.940	0.970	NF	1.000
FAPI-I-06	0.467	1.500	0.000	NF	1.000
FAPI-I-07	0.743	1.104	1.376	NF	1.000

FAPI: fungal acid phosphatase inhibitor; Class.: classification. C.L.: confidence level of the ANN classification; GATS3m: Geary's autocorrelation at topological lag 3 weighted by atomic masses; MATS6m: Moran's autocorrelation at lag 6 weighted by atomic masses; mindssC: Minimum E-State value for doubly bonded sp^2 carbons (=C<).

Table S33. Classification and probability of classification for training set compounds in Model 21 (LDA-Mordred) trained with experimental fungicide activity data, focusing on Fungicide activity prediction.

Compound	ATSC6p	MATS3se	n6ARing	DF	Class.	C.L.
**Active Group**						
FAPI-I-12	-0.507	-0.041	1	-1.950	I	0.876
FAPI-I-13	3.643	-0.288	1	-0.466	I	0.614
FAPI-I-14	-2.574	0.135	0	0.638	F	0.654
FAPI-I-02	0.420	0.024	0	3.065	F	0.955
FAPI-I-15	1.192	0.133	1	4.219	F	0.985
FAPI-I-18	1.192	0.133	1	4.219	F	0.985
FAPI-I-16	2.426	-0.065	0	4.416	F	0.988
FAPI-I-17	4.622	-0.179	0	5.541	F	0.996
**Inactive Group**						
FAPI-I-03	0.000	-0.397	0	-6.116	I	0.998
FAPI-I-08	1.544	-0.008	4	-4.856	I	0.992
FAPI-I-07	0.000	-0.175	1	-3.886	I	0.980
FAPI-I-06	1.867	-0.313	1	-3.739	I	0.977
FAPI-I-19	-0.799	0.072	2	-2.380	I	0.915
FAPI-I-09	-0.626	-0.157	0	-2.227	I	0.903
FAPI-I-11	0.000	-0.064	1	-1.632	I	0.836
FAPI-I-20	0.843	-0.015	2	-1.576	I	0.829
FAPI-I-04	-0.817	-0.099	0	-1.366	I	0.797
FAPI-I-10	-0.216	-0.021	1	-1.103	I	0.751
FAPI-I-05	2.258	-0.205	1	-0.959	I	0.723
FAPI-I-01	0.232	-0.097	0	0.323	F	0.580

FAPI: fungal acid phosphatase inhibitor; DF: discriminant function; C.L.: confidence level; Class.: Classification; ATSC6p: Centered autocorrelation of topological structure at lag 6 weighted by atomic polarizability; MATS3se: Moran autocorrelation at lag 3 weighted by Sanderson electronegativity; n6ARing: Number of 6-membered aromatic rings.

Table S34. Classification and probability of classification for training set compounds in Model 22 (ANN-Mordred) trained with experimental fungicide activity data, focusing on Fungicide activity prediction.

Compound	ATSC6p	MATS3se	n6ARing	Class.	C.L.
**Active Group**					
FAPI-I-13	3.643	-0.288	1	1	0.522
FAPI-I-14	-2.574	0.135	0	1	0.747
FAPI-I-02	0.420	0.024	0	1	0.978
FAPI-I-15	1.192	0.133	1	1	0.986
FAPI-I-16	2.426	-0.065	0	1	0.995
FAPI-I-12	-0.507	-0.041	1	2	0.865
**Inactive Group**					
FAPI-I-01	0.232	-0.097	0	1	0.758
FAPI-I-05	2.258	-0.205	1	2	0.636
FAPI-I-10	-0.216	-0.021	1	2	0.733
FAPI-I-09	-0.626	-0.157	0	2	0.804
FAPI-I-20	0.843	-0.015	2	2	0.887
FAPI-I-19	-0.799	0.072	2	2	0.954
FAPI-I-06	1.867	-0.313	1	2	0.963
FAPI-I-07	0.000	-0.175	1	2	0.975
FAPI-I-03	0.000	-0.397	0	2	0.994
FAPI-I-08	1.544	-0.008	4	2	0.999

FAPI: fungal acid phosphatase inhibitor; Class.: classification. C.L.: confidence level of the ANN classification; ATSC6p: Centered autocorrelation of topological structure at lag 6 weighted by atomic polarizability; MATS3se: Moran autocorrelation at lag 3 weighted by Sanderson electronegativity; n6ARing: Number of 6-membered aromatic rings.

Table S35. Classification and probability of classification for internal test set compounds in Model 20 (ANN-AlvaDesc) trained with experimental fungicide activity data, focusing on Fungicide activity prediction.

Compound	MATS6m	GATS3m	mindssC	Class.	C.L.
**Active Group**					
FAPI-I-18	1.216	0.947	0.177	F	1.000
**Inactive Group**					
FAPI-I-11	0.000	1.085	0.000	NF	0.982
FAPI-I-08	0.309	1.120	0.622	NF	1.000
FAPI-I-07	0.000	1.292	0.000	NF	1.000

FAPI: fungal acid phosphatase inhibitor; Class.: classification; C.L.: confidence level of the ANN classification; GATS3m: Geary's autocorrelation at topological lag 3 weighted by atomic masses; MATS6m: Moran's autocorrelation at lag 6 weighted by atomic masses; mindssC: Minimum E-State value for doubly bonded sp^2 carbons (=C<).

Table S36. Classification and probability of classification for internal test set compounds in Model 22 (ANN-Mordred) Trained with experimental fungicide activity data, focusing on Fungicide activity prediction.

Compound	ATSC6p	MATS3se	n6ARing	Class.	C.L.
**Active Group**					
FAPI-I-17	4.622	-0.179	0	1	0.999
FAPI-I-18	1.192	0.133	1	1	0.986
**Inactive Group**					
FAPI-I-04	-0.817	-0.099	0	2	0.648
FAPI-I-11	0.000	-0.064	1	2	0.816

FAPI: fungal acid phosphatase inhibitor; Class.: classification; C.L.: confidence level of the ANN classification; ATSC6p: Centered autocorrelation of topological structure at lag 6 weighted by atomic polarizability; MATS3se: Moran autocorrelation at lag 3 weighted by Sanderson electronegativity; n6ARing: Number of 6-membered aromatic rings.

Table S37. Second screening selection of potential **F**ungicides targeting **A**cid **P**hosphatase **I**nhibition (FAPI-II).

Compound	IUPAC name	Chemical structure
FAPI-II-01	2-((4R,7S)-1,3-dioxo-1,3,3a,4,7,7a-hexahydro-2H-4,7-methanoisoindol-2-yl)-1,1,3,3-tetramethylisouronium	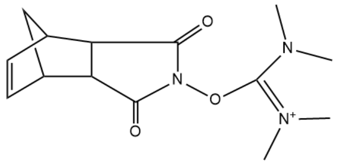
FAPI-II-02	2-(chloromethyl)morpholine	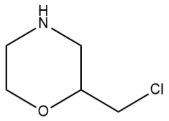
FAPI-II-03	2,6-dichloro-4-(chloroimino)cyclohexa-2,5-dien-1-one	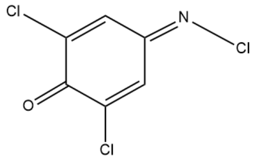
FAPI-II-04	N-(4-chloro-2-(trifluoromethyl)phenyl)-2-(o-tolyloxy)propanamide	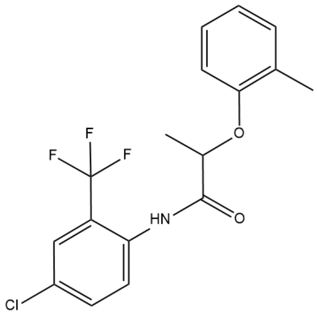
FAPI-II-05	N-(2-chloro-5-nitrophenyl)-2-nitrobenzenesulfonamide	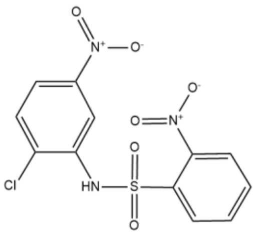
FAPI-II-06	(2R,3R,4S,5S)-2-((4-chloro-3-nitrophenyl)amino)tetrahydro-2H-pyran-3,4,5-triol	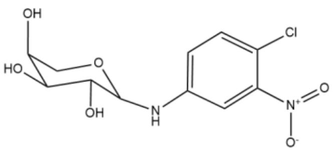

FAPI-II-07	N-(4-chloro-3-nitrophenyl)-2-fluorobenzenesulfonamide	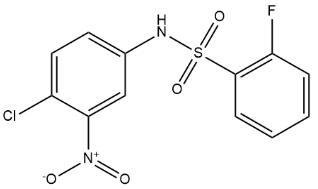
FAPI-II-08	5-butyl-2-thioxodihydropyrimidine-4,6(1H,5H)-dione	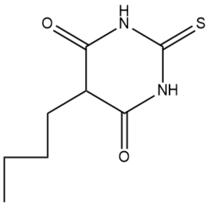
FAPI-II-09	ethyl 3-oxooctahydro-1H-cyclopenta[c]pyridine-4-carboxylate	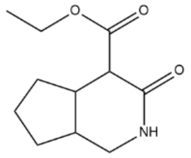
FAPI-II-10	4-(1,3-dioxo-1,3,3a,4,7,7a-hexahydro-2H-4,7-methanoisoindol-2-yl)butanoic acid	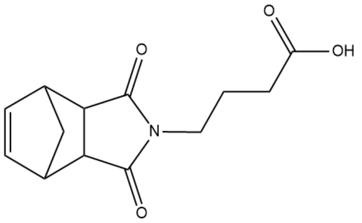
FAPI-II-11	Ethyl 2-(tetrazolo[1,5-a]quinoxalin-4-ylthio)acetate	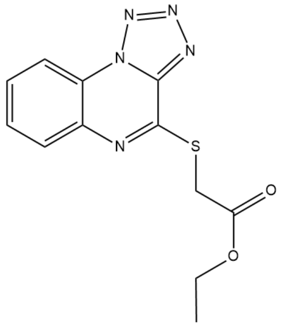
FAPI-II-12	N-(5-chloro-2-methoxyphenyl)-2-(5-(4-fluorophenyl)-4,6-dioxo-4,5,6,6a-tetrahydropyrrolo[3,4-d][1,2,3]triazol-1(3aH)-yl)acetamide	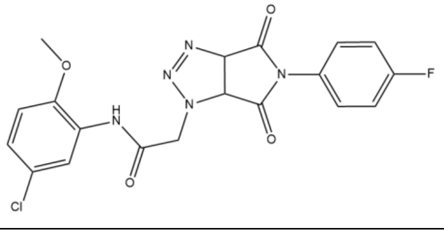
FAPI-II-13	1-(2-chloro-6-fluorobenzyl)-5-(4-methoxyphenyl)-3a,6a-dihydropyrrolo[3,4-d][1,2,3]triazole-4,6(1H,5H)-dione	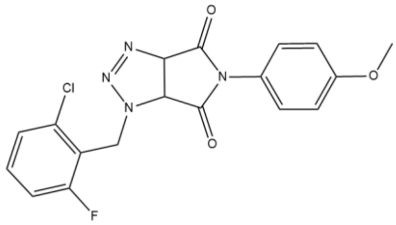

FAPI-II-14	N-(2-(4-oxobenzo[d][1,2,3]triazin-3(4H)-yl)ethyl)ethanesulfonamide	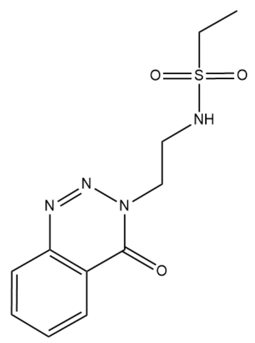

Table S38. Classification and Probability of Classification for the Second Virtual Screening Focused on Fungicide Activity Prediction as Part of the *In Silico* Strategy to Identify Novel FAPI Compounds Using AlvaDesc QSAR Models.

Compound	Model 1 LDA			Model 2 ANN		Model 3 LDA			Model 4 ANN		Model 5 LDA			Model6 ANN	
	DF_1	Cl.	P.A.	Cl.	C.L.	DF_3	Cl.	P.A.	Cl.	C.L.	DF_5	Cl.	P.A.	Cl.	C.L.
fluopyram	-0.991	NF	0.275	F	0.505	-0.349	NF	0.410	NF	0.547	3.054	F	0.955	F	0.730
FAPI-II-1	-0.457	NF	0.388	NF	0.514	-1.481	NF	0.185	NF	0.731	0.650	F	0.657	F	0.577
FAPI-II-2	-2.334	NF	0.088	NF	0.596	-0.884	NF	0.285	NF	0.604	-0.952	NF	0.278	NF	0.764
FAPI-II-3	-0.829	NF	0.309	NF	0.540	1.748	F	0.846	F	0.726	0.841	F	0.697	NF	0.726
FAPI-II-4	-0.654	NF	0.347	F	0.519	0.296	F	0.569	NF	0.674	0.149	F	0.536	NF	0.726
FAPI-II-5	2.273	F	0.908	F	0.612	0.402	F	0.595	F	0.589	0.602	F	0.645	NF	0.726
FAPI-II-6	-0.055	NF	0.489	F	0.524	-0.082	NF	0.475	NF	0.657	-0.422	NF	0.396	NF	0.727
FAPI-II-7	0.601	F	0.650	F	0.553	-0.059	NF	0.481	NF	0.516	0.532	F	0.629	NF	0.726
FAPI-II-8	-1.559	NF	0.174	NF	0.559	0.464	F	0.609	F	0.621	-1.630	NF	0.164	NF	0.573
FAPI-II-9	-1.603	NF	0.168	NF	0.557	0.149	F	0.537	F	0.633	-1.030	NF	0.263	NF	0.691
FAPI-II-10	-0.920	NF	0.286	NF	0.526	-1.938	NF	0.126	NF	0.730	-1.037	NF	0.262	NF	0.688
FAPI-II-11	1.065	F	0.747	F	0.565	1.036	F	0.736	NF	0.512	4.189	F	0.985	F	1.000
FAPI-II-12	0.823	F	0.699	F	0.564	-1.115	NF	0.244	NF	0.727	-0.496	NF	0.378	NF	0.728
FAPI-II-13	1.037	F	0.742	F	0.573	-0.281	NF	0.426	NF	0.627	-0.260	NF	0.435	NF	0.726
FAPI-II-14	0.165	F	0.545	F	0.534	-1.030	NF	0.262	NF	0.724	-1.697	NF	0.155	NF	0.579

D.F.: discriminant function; P.A.: probability of being active as fungicide by LDA model; Cl.: classification; C.L.: confidence level of the ANN classification; F: fungicide; NF: no fungicide.

Table S39. Classification and Probability of Classification for the Second Virtual Screening Focused on Fungicide Activity Prediction as Part of the *In Silico* Strategy to Identify Novel FAPI Compounds Using Mordred QSAR Models.

Compound	Model 7 LDA			Model 8 ANN		Model 9 LDA			Model 10 ANN		Model 11 LDA			Model 12 ANN	
	DF_7	Cl.	P.A.	Cl.	C.L.	DF_9	Cl.	P.A.	Cl.	C.L.	DF_{10}	Cl.	PA	Cl.	C.L.
fluopyram	0.962	F	0.727	F	0.752	0.074	F	0.518	1	0.803	1.956	F	0.876	F	0.8
FAPI-II-1	1.481	F	0.817	F	0.641	0.025	F	0.506	2	0.608	2.355	F	0.914	F	0.712
FAPI-II-2	-1.642	NF	0.163	NF	0.732	-0.94	NF	0.281	2	0.924	-0.814	NF	0.308	NF	0.724
FAPI-II-3	0.982	F	0.73	F	0.654	1.586	F	0.83	2	1	1.446	F	0.809	NF	0.792
FAPI-II-4	-2.435	NF	0.081	NF	0.65	0.963	F	0.724	2	0.509	-0.235	NF	0.442	F	0.714
FAPI-II-5	1.19	F	0.769	F	0.65	0.559	F	0.636	1	0.779	0.722	F	0.674	F	0.736
FAPI-II-6	-0.775	NF	0.318	NF	0.667	-0.204	NF	0.449	2	0.661	2.729	F	0.939	F	0.575
FAPI-II-7	0.56	F	0.639	F	0.64	0.727	F	0.674	1	0.814	0.762	F	0.682	F	0.725
FAPI-II-8	-1.065	NF	0.258	NF	0.728	1.028	F	0.736	1	0.902	1.117	F	0.754	F	0.723
FAPI-II-9	-1.557	NF	0.175	NF	0.738	-0.776	NF	0.315	1	0.871	-1.584	NF	0.171	NF	0.716
FAPI-II-10	0.082	F	0.523	F	0.542	-0.705	NF	0.331	2	0.922	0.859	F	0.703	NF	0.567
FAPI-II-11	-1.051	NF	0.261	NF	0.727	0.014	F	0.503	1	0.82	3.279	F	0.964	F	0.736
FAPI-II-12	0.546	F	0.636	F	0.639	-0.122	NF	0.47	2	0.53	2.818	F	0.944	F	0.736
FAPI-II-13	0.262	F	0.568	F	0.592	0.315	F	0.578	1	0.907	3.89	F	0.98	F	0.702
FAPI-II-14	-0.979	NF	0.275	NF	0.716	-0.584	NF	0.358	2	0.604	4.132	F	0.984	F	0.662

D.F.: discriminant function; P.A.: probability of being active as fungicide by LDA model; Cl.: classification; C.L.: confidence level of the ANN classification; F: fungicide; NF: no fungicide.

Table S40. Classification and Probability of Classification for the Second Virtual Screening Focused on Acid Phosphatase Inhibitory activity prediction as Part of the *In Silico* Strategy to Identify Novel FAPI Compounds Using AlvaDesc and Mordred QSAR Models.

Compound	AlvaDesc							Mordred						
	Model 13 LDA			Model 14 ANN		Model 15 ANN		Model 16 LDA			Model 17 ANN		Model 18 ANN	
	DF_{17}	Cl.	P.A.	Cl.	C.L.	Cl.	C.L.	DF_{20}	Cl.	P.A.	Cl.	C.L.	Cl.	C.L.
FAPI-II-1	-4.214	I	0.015	I	1.000	I	0.538	-0.141	I	0.465	API	1.000	API	1.000
FAPI-II-2	1.763	API	0.854	API	1.000	I	0.712	1.537	API	0.823	API	0.969	API	1.000
FAPI-II-3	-1.024	I	0.264	API	0.786	I	0.724	-2.523	I	0.074	I	1.000	I	1.000
FAPI-II-4	-3.673	I	0.025	I	1.000	I	0.537	1.162	API	0.762	I	1.000	I	0.512
FAPI-II-5	-1.057	I	0.258	API	0.994	API	0.586	0.174	API	0.543	I	1.000	API	1.000
FAPI-II-6	4.085	API	0.984	API	1.000	API	0.742	2.344	API	0.913	API	1.000	API	1.000
FAPI-II-7	1.110	API	0.752	API	0.996	I	0.727	-0.115	I	0.471	API	1.000	API	1.000
FAPI-II-8	-2.462	I	0.079	I	1.000	I	0.803	-3.910	I	0.020	API	1.000	I	1.000
FAPI-II-9	-1.256	I	0.222	API	0.569	I	0.681	-2.447	I	0.080	I	1.000	API	0.659
FAPI-II-10	-0.057	I	0.486	I	1.000	I	0.865	-2.795	I	0.058	API	0.896	I	1.000
FAPI-II-11	-3.271	I	0.037	API	0.945	I	0.638	0.954	API	0.722	I	1.000	I	0.589
FAPI-II-12	-1.254	I	0.222	I	0.997	I	0.840	-1.378	I	0.201	I	1.000	API	0.962
FAPI-II-13	-0.964	I	0.276	I	0.999	I	0.864	-1.943	I	0.125	API	1.000	API	0.873
FAPI-II-14	-0.253	I	0.437	API	0.925	I	0.848	1.127	API	0.755	I	0.572	API	1.000
L-(+)-tartaric acid	3.279	API	0.964	API	1	API	0.749	0.961	API	0.723	API	1.000	API	0.959

DF: discriminant function; P.A.: probability of being active as fungicide by LDA model; C.L.: confidence level of the ANN classification; API: acid phosphatase inhibitor; I: inactive

Table S41. Classification and Probability of Classification for the Second Virtual Screening Focused on experimental fungicide activity prediction as Part of the *In Silico* Strategy to Identify Novel FAPI Compounds Using AlvaDesc and Mordred QSAR Models.

**Compound**	**AlvaDesc**					**Mordred**				
	**Model 19 LDA**			**Model 20 ANN**		**Model 21 LDA**			**Model 22 ANN**	
	**DF_{19}**	**Cl.**	**P.A.**	**Cl.**	**C.L.**	**DF_{21}**	**Cl.**	**P.A.**	**Cl.**	**C.L.**
fluopyram	12.031	F	1.000	F	1.000	0.841	F	0.699	F	0.823
FAPI-II-1	7.348	F	0.999	F	1.000	10.214	F	1.000	F	1.000
FAPI-II-2	9.65	F	1.000	F	1.000	-0.304	NF	0.424	NF	0.561
FAPI-II-3	-1.756	NF	0.147	F	0.967	-3.973	NF	0.018	NF	0.978
FAPI-II-4	5.613	F	0.996	F	1.000	-4.402	NF	0.012	NF	0.979
FAPI-II-5	-1.207	NF	0.230	F	0.805	-0.521	NF	0.372	F	0.582
FAPI-II-6	2.236	F	0.903	F	1.000	-0.521	NF	0.372	NF	0.625
FAPI-II-7	-0.485	NF	0.381	F	0.975	0.142	F	0.535	F	0.739
FAPI-II-8	3.29	F	0.964	F	1.000	-4.218	NF	0.015	NF	0.984
FAPI-II-9	-0.182	NF	0.455	F	0.998	0.179	F	0.545	F	0.596
FAPI-II-10	4.574	F	0.990	F	1.000	-1.987	NF	0.120	NF	0.785
FAPI-II-11	4.592	F	0.990	F	1.000	-4.817	NF	0.008	NF	0.985
FAPI-II-12	2.73	F	0.939	F	1.000	1.204	F	0.769	F	0.898
FAPI-II-13	4.784	F	0.992	F	1.000	0.793	F	0.688	F	0.846
FAPI-II-14	-2.076	NF	0.111	F	0.867	-1.703	NF	0.154	NF	0.702

D.F.: discriminant function; P.A.: probability of being active as fungicide by LDA model; Cl.: classification; C.L.: confidence level of the ANN classification; F: fungicide; NF: no fungicide.

Table S42. Docking score values (kcal/mol) and amino acid interactions between acid phosphatase from *Aspergillus niger* (PDB: 1QFX) and *Podosphaera xanthii* (homology model 15569).

**Compound**	**1QFX**		**15569**	
	**Docking score (kcal/mol)**	**AA Interact.**	**Docking score (kcal/mol)**	**AA Interact.**
**Phytic Acid**	-2.356	Hb:<br>Ser69, **Arg66** (2 interactions), Asn275, **Arg156** (2 interactions), **Asp139,** Tyr154<br>Sb:<br>**Arg66, Arg156**	-6.561	Hb:<br>**Arg162, Arg55** (3 interactions), **Asp63,** Gln18, Lys301 (2 interactions), **Glu305, Arg226** (3 interactions)<br>Sb:<br>Lys214, Lys301 (2 interactions)<br>AHb:<br>Tyr302, **His363**
**1st Virtual Screening**				
**FAPI-I-02**	-3.094	Hb:<br>**Arg66** (2 interactions), **Asp319**	-2.796	Hb:<br>Arg66, **Glu305,** Tyr302
**FAPI-I-12**	-3.498	Xb:<br>**Arg156**	-3.887	Hb:<br>**Arg226,** Lys214<br>Xb:<br>Lys301
**FAPI-I-13**	-4.718	Hb:<br>Ser71, Ser69 (2 interactions), **Arg66** (3 interactions), **Arg156** (2 interactions), **Asp319,** Thr320, Asn275<br>Sb:<br>**Arg62, Arg156** (2 interactions), **Arg66** (2 interactions)	-6.566	Hb:<br>Asp63, Lys301 (2 interactions), **Arg55** (2 interactions), **Arg226** (3 interactions), Lys214, Asp364, Glu201, Gln160, **Arg162**<br>Sb:<br>Lys301(2 interactions), Arg226 (2 interactions), Lys214, **Arg55,** Arg51, **Arg162** (2 interactions),<br>AHb:<br>His52
**FAPI-I-14**	-3.407	Hb:<br>Asn321	-4.219	Hb:<br>Leu62, **Arg226**<br>Sb:<br>Lys301, Arg226, Lys214
**FAPI-I-15**	-3.638	AHb:<br>Tyr154, Thr320<br>Pi-pi:<br>**His318**	-4.713	Hb:<br>Leu62<br>Pi-C+:<br>Lys301
**FAPI-I-16**	-3.527	Hb:<br>**Arg66**<br>AHb:<br>Phe246	-4.112	Hb:<br>**Arg226,** Lys214
**FAPI-I-17**	-2.969	Hb:<br>**Arg66, Asp319**<br>AHb:<br>**Glu272, His318,** Asn33, **Asp319,** Tyr276	-4.283	Hb:<br>**Glu305**<br>AHb:<br>**Glu305**<br>Pi-C+:

		Pi-pi:<br>**His318**<br>Pi-C+:<br>**Arg156**		Lys301, Lys230
**FAPI-I-18**	-3.497	AHb:<br>**Asp319,** Thr320<br>Pi-pi:<br>**His318**	-4.717	Hb:<br>Leu62<br>Pi-C+:<br>Lys301
**2nd virtual screening***				
**FAPI-II-02**	-5.173	Hb:<br>Asp457, Glu184<br>Sb:<br>Asp457, Glu184	-4.168	Hb:<br>Glu201<br>Xb:<br>Asn366<br>Sb:<br>Glu201
**FAPI-II-05**	-2.55	Hb:<br>Asn112, Ser69, **Arg66**<br>Sb:<br>**Arg66, Arg156**<br>Pi-pi:<br>Tyr154<br>AHb:<br>Asp139	-3.107	Hb:<br>**Arg226, Arg55** (2 interactions), His52<br>Sb:<br>Arg226 (2 interactions), Lys214, Arg55, Arg162<br>Xb:<br>Lys301<br>AroHb:<br>Gln160, His363, His52
**FAPI-II-09**	-4.375	Hb:<br>Asn321 (2 interactions)	-4.477	Hb:<br>Gln61, Glu305
**FAPI-II-13**	-2.672	Hb:<br>Asn275<br>AHb:<br>**Glu272, Asp319**<br>Pi-C+:<br>**Arg156**	-3.601	Hb:<br>Leu62, Lys301<br>Pi-C+:<br>Lys301, **Arg226**<br>AroHb:<br>Glu305

Hb: hydrogen bond; AHb: aromatic hydrogen bond; Sb: salt bridge (ionic bond); pi-C*: pi- cation interaction; pi-pi: pi-pi stacking; Xb: halogen bonds; Bold AA: active site from 1QFX (catalytic function) and potential active site of 15569; Underscore AA: substrate specificity site function.

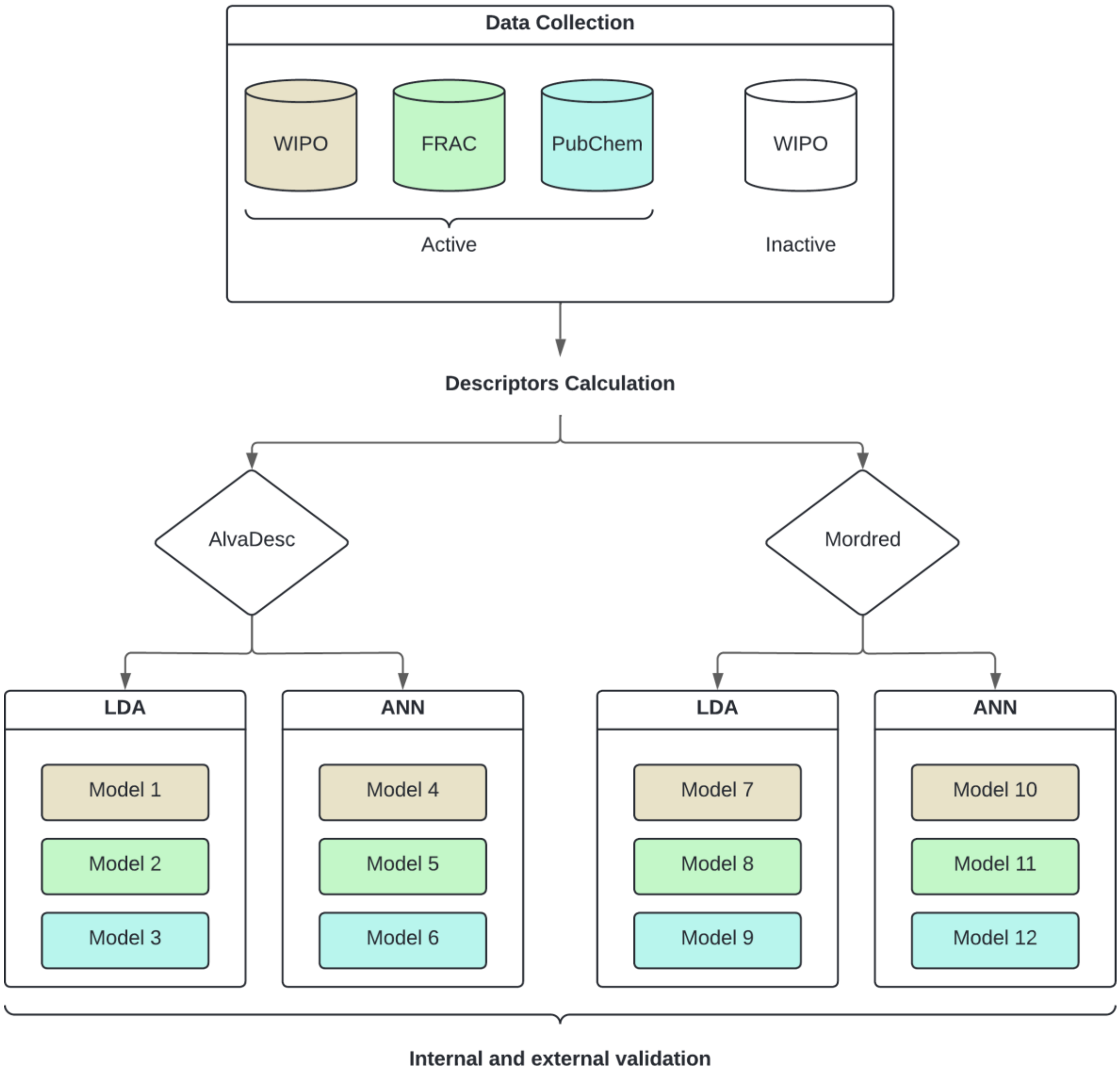

Figure S1. Machine Learning and AI-Driven QSAR Modeling strategy for identifying novel compounds with fungicide activity.

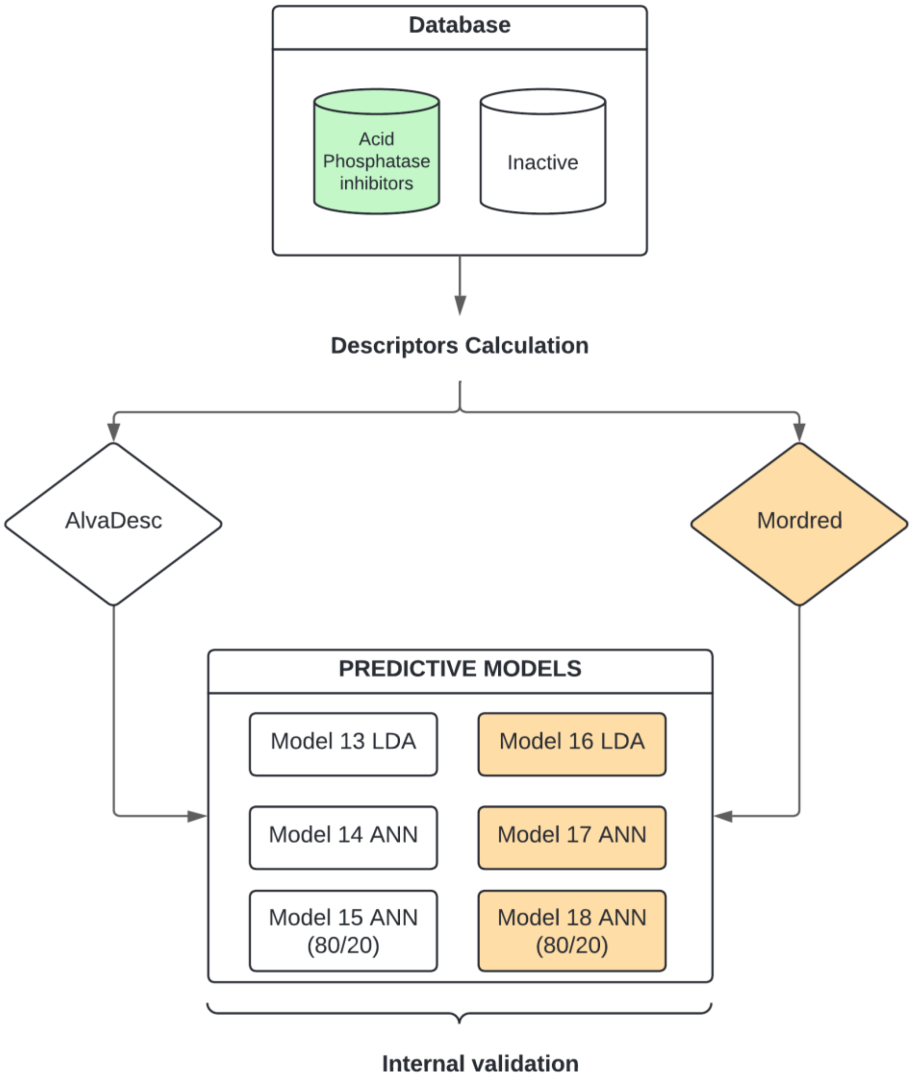

Figure S2. Machine Learning and AI-Driven QSAR Modeling strategy for the discovery of novel acid phosphatase inhibitors.

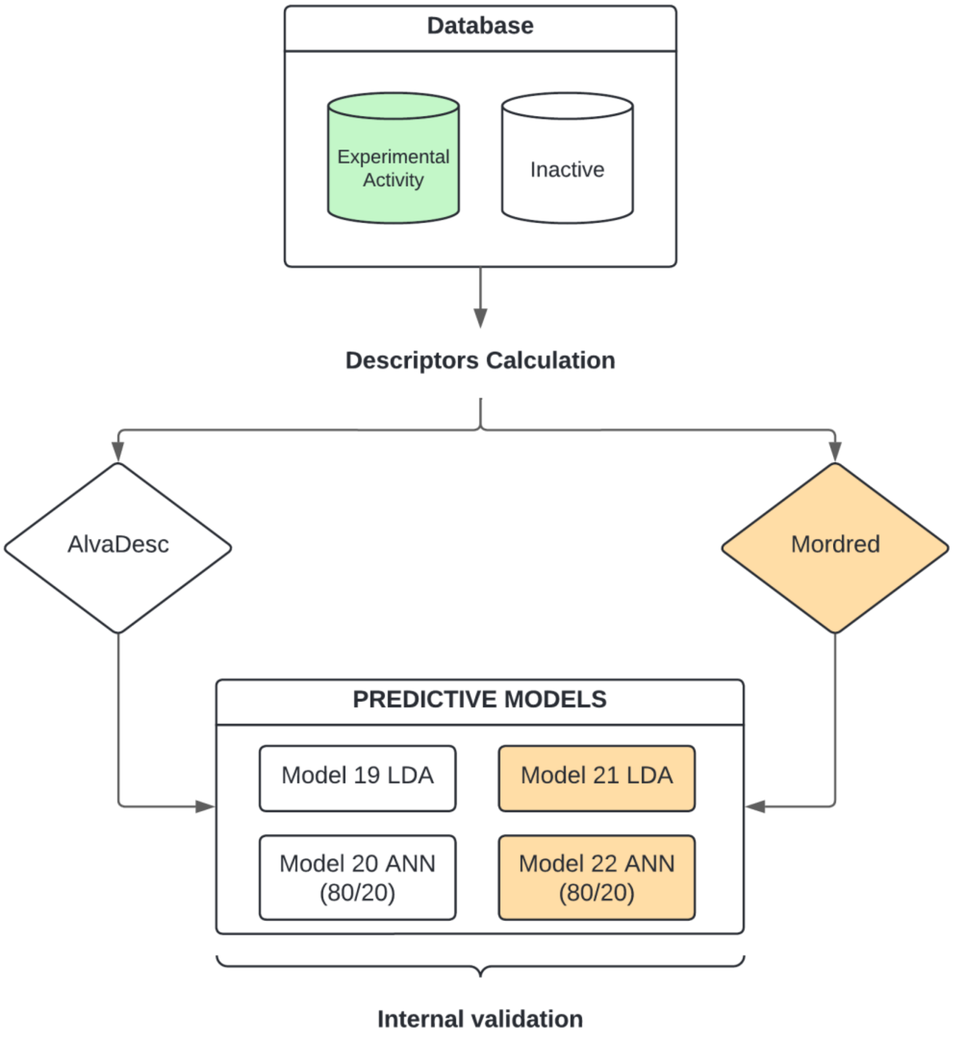

Figure S3. Machine Learning and AI-Driven QSAR Modeling strategy for identifying novel compounds with fungicide activity based on UMA's experimental results.

Molecular docking

As a methodology to validate the predicted mechanism of action of the newly **FAPI (F**ungicide targeting **A**cid **P**hosphatase **I**nhibition) compounds, a molecular docking study was conducted to analyze the potential interactions between the identified fungicides and the acid phosphatase enzyme. This proposed novel mechanism of action could lead to the introduction of fungicides targeting a new pathway—acid phosphatase inhibition.

To achieve this, the interactions of the enzyme's natural ligand, phytate or phytic acid, with acid phosphatase were first examined. These interactions, critical for catalytic activity, were then compared with those established by the novel fungicides. The comparison was based on docking scores and amino acid interactions, providing insight into the potential inhibitory mechanism of the new compounds.

To this end, as detailed in the Materials and Methods section, two acid phosphatase models were selected, each corresponding to a different fungal species: 1QFX (PDB code) from *Aspergillus niger*, and 15569, a structure predicted through homology modeling for *Podosphaera xanthii*.

The docking simulation grid box for 1QFX was configured with coordinates (23.89, 72.33, 69.28) for the x, y, and z axes, respectively. For the 15569 acid phosphatase model, the grid box coordinates were set at (-0.72, 1.11, 12.43).

The results of the molecular docking study using the crystallized structure of acid phosphatase from *Aspergillus niger* (PDB: 1QFX) are analyzed. As shown in Table S42, the docking score for the enzyme's natural substrate, phytic acid, is -2.356 kcal/mol. The FAPIs that exhibited higher experimental fungicidal activity were analyzed through molecular docking studies, and their interactions with the enzyme were evaluated. The results indicate that these compounds achieved more favorable docking scores compared to the substrate.

The interactions between the substrate and the enzyme were analyzed. As shown in Figure 5 and Table S42, phytic acid forms various interactions with key amino acids, including Arg66, Arg156, and Asp139, which are critical for the enzyme's catalytic activity (*Aspergillus niger* acid phosphatase, PDB: 1QFX) (47-49).

Analysis of the interactions between the identified potential fungicides (FAPI) and *Aspergillus niger* acid phosphatase (Table S42) indicates that all compounds, except for three (FAPI-I-14, FAPI-II-2, and FAPI-II-9), establish interactions with key active site residues (Arg62, His63, Arg66, Asp75, Arg156, Glu272, His318, and Asp319). Figure 5 highlights the bond interactions of the FAPI-I-13 compound, which achieved the most favorable docking score (FAPI-I-13, -4.718 kcal/mol). This potential fungicide, identified using an *in silico* methodology based on ML and Ai driven-QSAR models, interacts with the catalytic site amino acids Arg62, Arg66, Arg156, and Asp319.

In addition, two FAPI compounds — FAPI-17 and FAPI-II-13 — interact with one of the two substrate specificity residues of 1QFX, namely Asp75 and Glu272 (Table S42). This interaction is particularly relevant, as these residues play a key role in recognizing and positioning the substrate within the active site.

A parallel molecular docking study was performed using the hypothetical structure of acid phosphatase from *Podosphaera xanthii* (model ID:15569). In this analysis (Table S42), the enzyme's substrate, phytic acid, demonstrated a highly favorable docking score of -6.561 kcal/mol. Among the evaluated compounds, FAPI-I-13 achieved a comparable docking score, slightly exceeding that of the substrate. Although the specific amino acids critical for the catalytic activity of this enzyme, modeled through homology, remain unidentified, it can be inferred, based on analogy with the key residues identified in *Aspergillus niger*, that the following residues might play a crucial role in the catalytic mechanism of *P. xanthii* acid phosphatase:

- **Arg55 and Arg226**: These residues are proposed to play a crucial role in catalytic activity, as the substrate (Phytic Acid) forms three hydrogen bonds with them. Several tested compounds, including FAPI-I-12, FAPI-I-13, FAPI-I-14, FAPI-I-16, FAPI-II-5, and FAPI-II-13, also interact with these residues. Notably, FAPI-I-13, which demonstrates the most favorable docking score (-6.555 kcal/mol), replicates the same interactions with the three arginine residues identified as potentially key for catalytic activity: Arg55, Arg126, and Arg226.
- **Asp63**: This residue is also suggested to be essential for the enzyme's catalytic function, based on its interaction with the substrate. FAPI-I-13, the only compound with a docking score more favorable than that of the substrate, forms a significant interaction with this residue.
- **Glu305**: Hydrogen bond interactions between this residue and the enzyme's substrate support its proposed functional importance. Identified potential fungicides such as FAPI-I-02, FAPI-I-17, and FAPI-II-9 also establish hydrogen bonds with Glu305, further highlighting its relevance. This residue could potentially function as a substrate specificity site, analogous to Glu272 in the *Aspergillus niger* acid phosphatase (PDB ID: 1QFX), which is known to play a critical role in substrate recognition.
- **His363**: Phytic acid forms a bond with this residue, supporting its role in catalytic activity, similar to the actions of His63 and His318 in *Aspergillus niger* acid phosphatase (PDB ID: 1QFX). Similarly, FAPI-II-5 interacts with His363, reinforcing its potential mechanism of action as a fungicide targeting acid phosphatase.

These findings suggest that the identified FAPI compounds, particularly FAPI-I-13, may effectively exhibit fungicidal activity by inhibiting the acid phosphatase of *Podosphaera xanthii*, potentially by replicating or enhancing the interactions formed by the natural substrate.

In addition to the amino acids that, by analogy with acid phosphatases from other fungal species such as *Aspergillus niger*, are presumed to be critical for catalytic activity, several residues appear to be particularly relevant for the catalytic function of the *P. xanthii* acid phosphatase. These include:

- **Lys301**: The enzyme’s substrate, phytic acid, forms two hydrogen bonds with this residue. Several compounds, including FAPI-I-12, FAPI-I-13 (with two interactions, mirroring the substrate), FAPI-I-14, FAPI-I-15, FAPI-I-17, FAPI-I-18, FAPI-II-5, and FAPI-II-13, also interact with Lys301.
- **Lys214**: This amino acid interacts with the substrate, and several fungicidal compounds, including FAPI-I-12, FAPI-I-13, FAPI-I-14, FAPI-I-16, and FAPI-II-5, establish bonds with it as well.
- **Tyr302**: Both the substrate and the compound FAPI-I-02 interact with this residue.

These observations strengthen the hypothesis that interactions with these specific amino acids could be essential for the catalytic activity of *P. xanthii* acid phosphatase and may play a critical role in the development of fungicidal activity.
